# Supplementary figures and images for: Identification of a highly efficient chloroplast-targeting peptide for plastid engineering
Source: PLoS Biol. 2024 Sep 19;22(9):e3002785. doi: 10.1371/journal.pbio.3002785 (PMC11444414; doi:10.1371/journal.pbio.3002785)

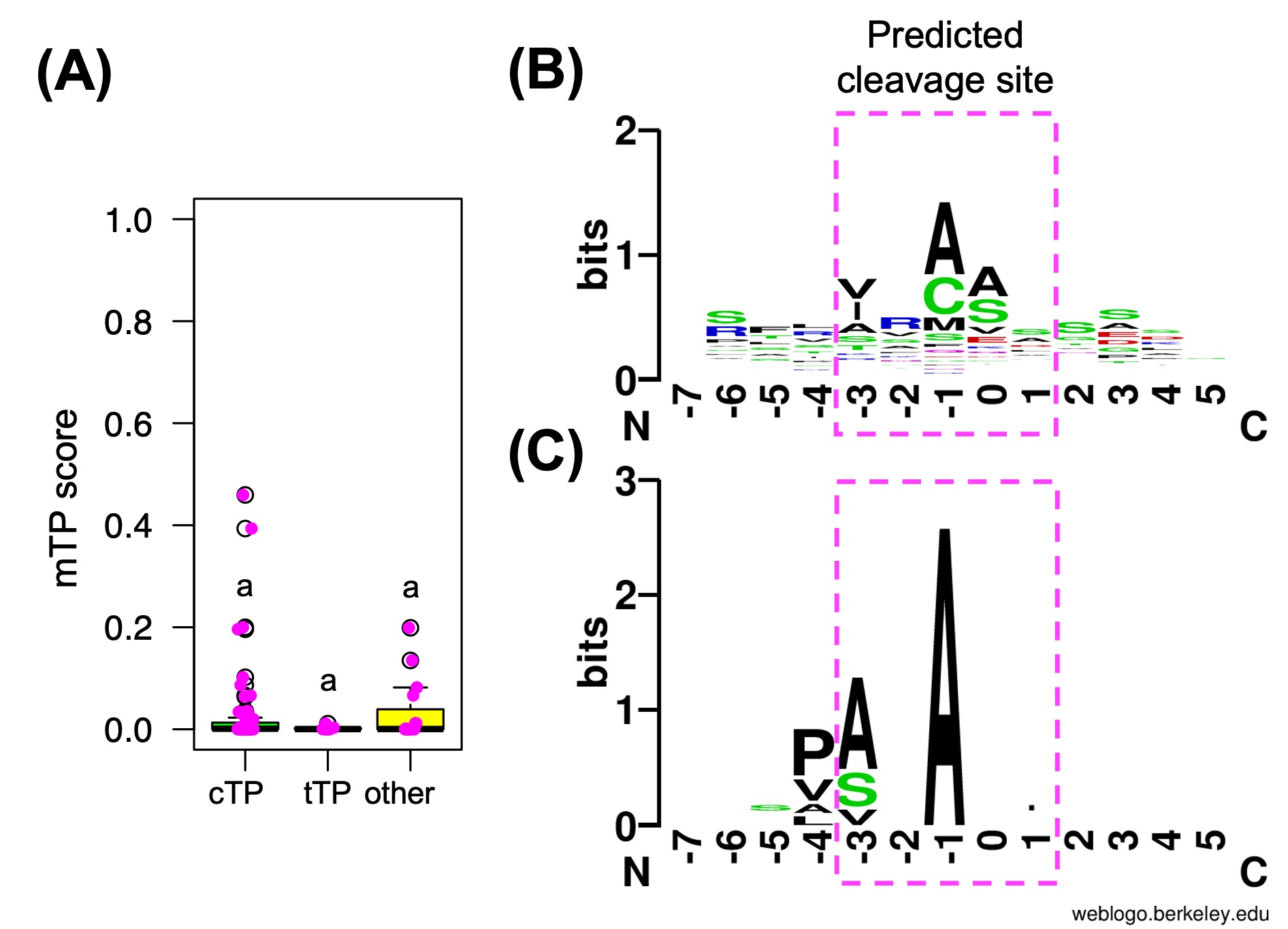

Supplement: S1 Fig — (A) Distribution of mTP scores in 89 polypeptides predicted by TargetP-2.0. The mTP score of each transit peptide (as presented in S3 Data) is shown as a box plot. Magenta dots represent the mTP scores of each peptide predicted to target the chloroplast (cTP), thylakoid lumen (tTP), and other organelles in plant cells (other). Significant differences are indicated by different letters in the boxplot (one-way ANOVA with Tukey’s HSD test at p = 0.05). (B) Amino acid distribution in 13 amino acids, which encompassed the predicted cleavage sites of each transit peptides (cTPs). (C) Alignment of the predicted cleavage sites of 8 tTPs. Multiple sequence alignments were performed using WebLogo. Five amino acids positioned within the magenta box at −3 to 1 represent the predicted cleavage site of the cTPs or tTPs with an extension of 4 amino acids applied to both its N- (−7 to −4) and C-termini (2 to 5). Amino acid at position 0 is the cleaved amino acid, which is the last amino acid attached to each transit peptide after cleavage. (JPG) [file pbio.3002785.s001.jpg]

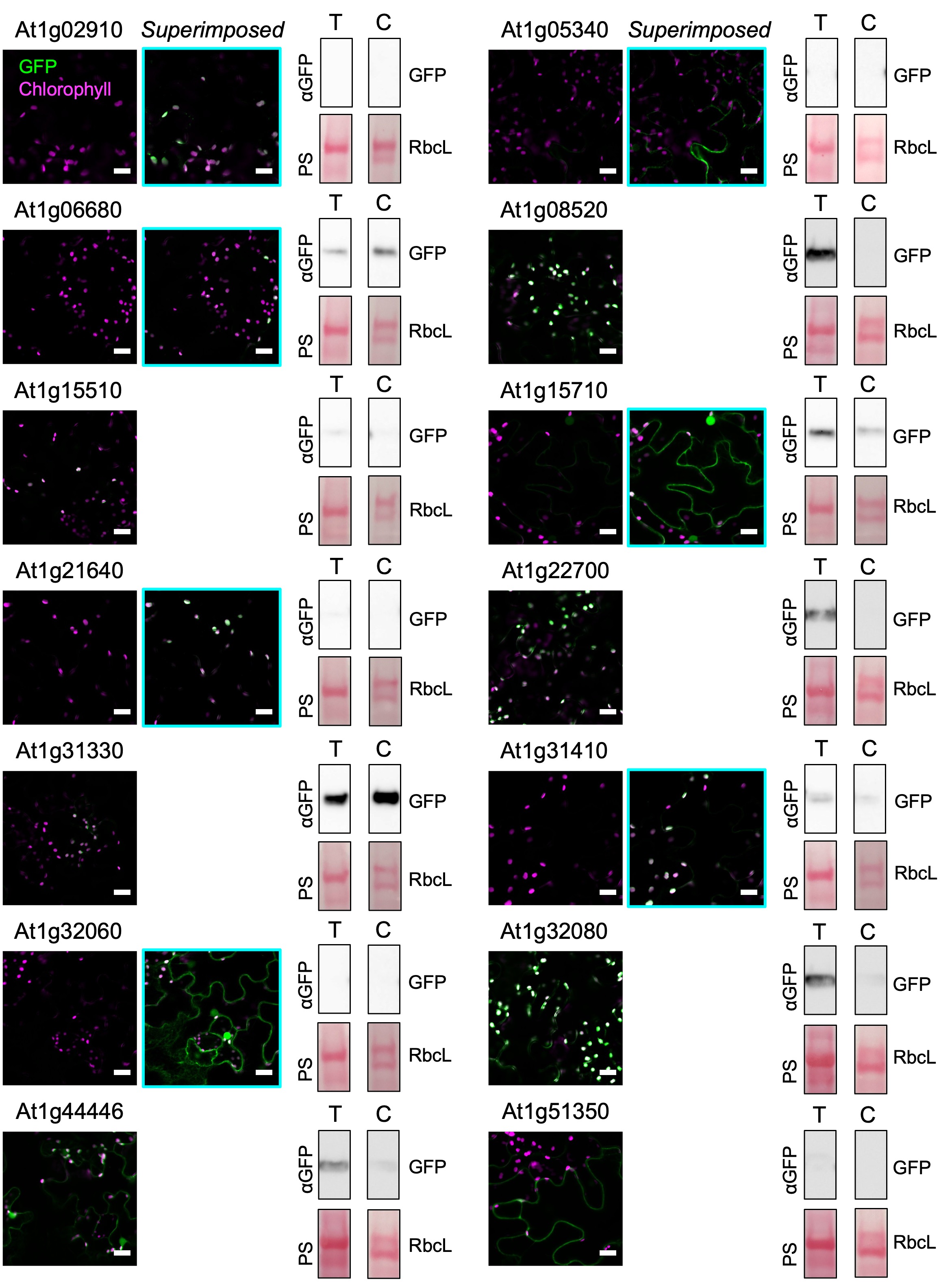

Supplement: S2 Fig — Subcellular localizations of different cTP-GFP(S65T) proteins in tobacco leaf cells at 4 days post agroinfiltration were observed under a confocal laser-scanning microscope (LSM 700). Scale bars = 20 μm. Immunoblot analysis of total leaf proteins (T) and isolated chloroplast proteins (C) showing the abundances of cTP-GFP(S65T) proteins in the cytosol and chloroplasts after translocation. αGFP = immunoblotted membranes probed by anti-GFP antibody. PS = Ponceau S-stained membrane to show the equal loading of protein samples. (JPG) [file pbio.3002785.s002.jpg]

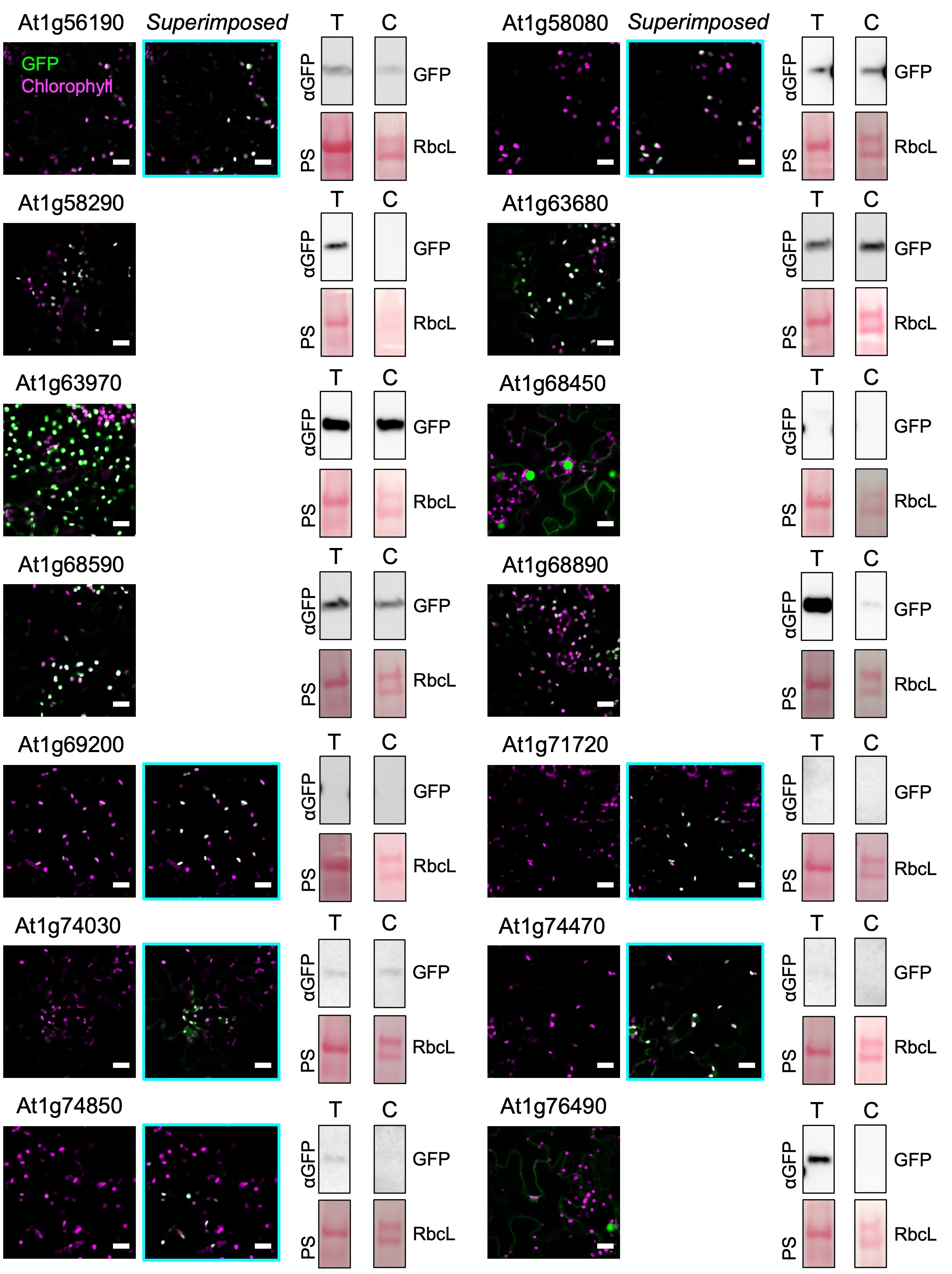

Supplement: S3 Fig — Subcellular localizations of different cTP-GFP(S65T) proteins in tobacco leaf cells at 4 days post agroinfiltration were observed under a confocal laser-scanning microscope (LSM 700). Scale bars = 20 μm. Immunoblot analysis of total leaf proteins (T) and isolated chloroplast proteins (C) showing the abundances of cTP-GFP(S65T) proteins in the cytosol and chloroplasts after translocation. αGFP = immunoblotted membranes probed by anti-GFP antibody. PS = Ponceau S-stained membrane to show the equal loading of protein samples. (JPG) [file pbio.3002785.s003.jpg]

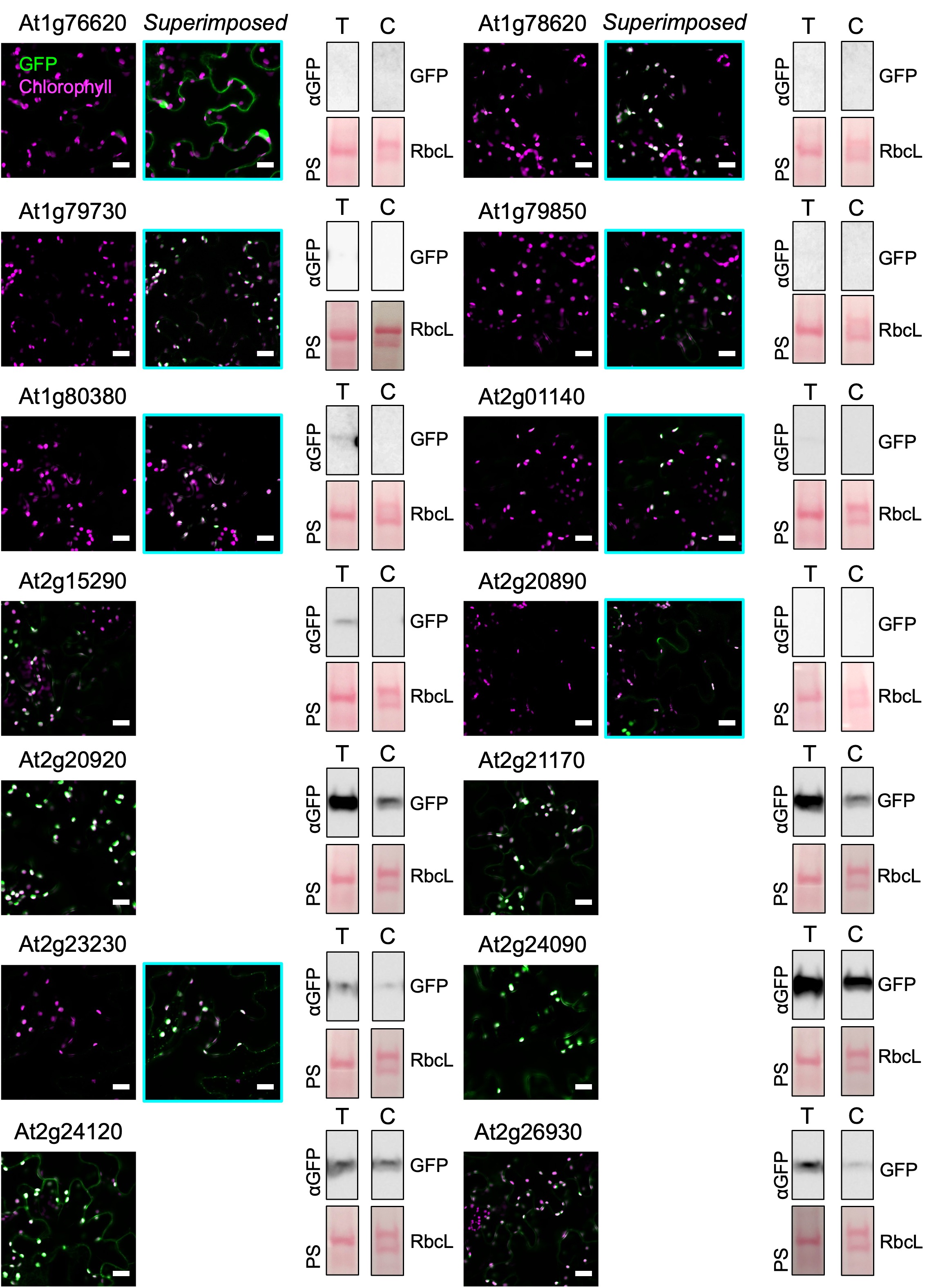

Supplement: S4 Fig — Subcellular localizations of different cTP-GFP(S65T) proteins in tobacco leaf cells at 4 days post agroinfiltration were observed under a confocal laser-scanning microscope (LSM 700). Scale bars = 20 μm. Immunoblot analysis of total leaf proteins (T) and isolated chloroplast proteins (C) showing the abundances of cTP-GFP(S65T) proteins in the cytosol and chloroplasts after translocation. αGFP = immunoblotted membranes probed by anti-GFP antibody. PS = Ponceau S-stained membrane to show the equal loading of protein samples. (JPG) [file pbio.3002785.s004.jpg]

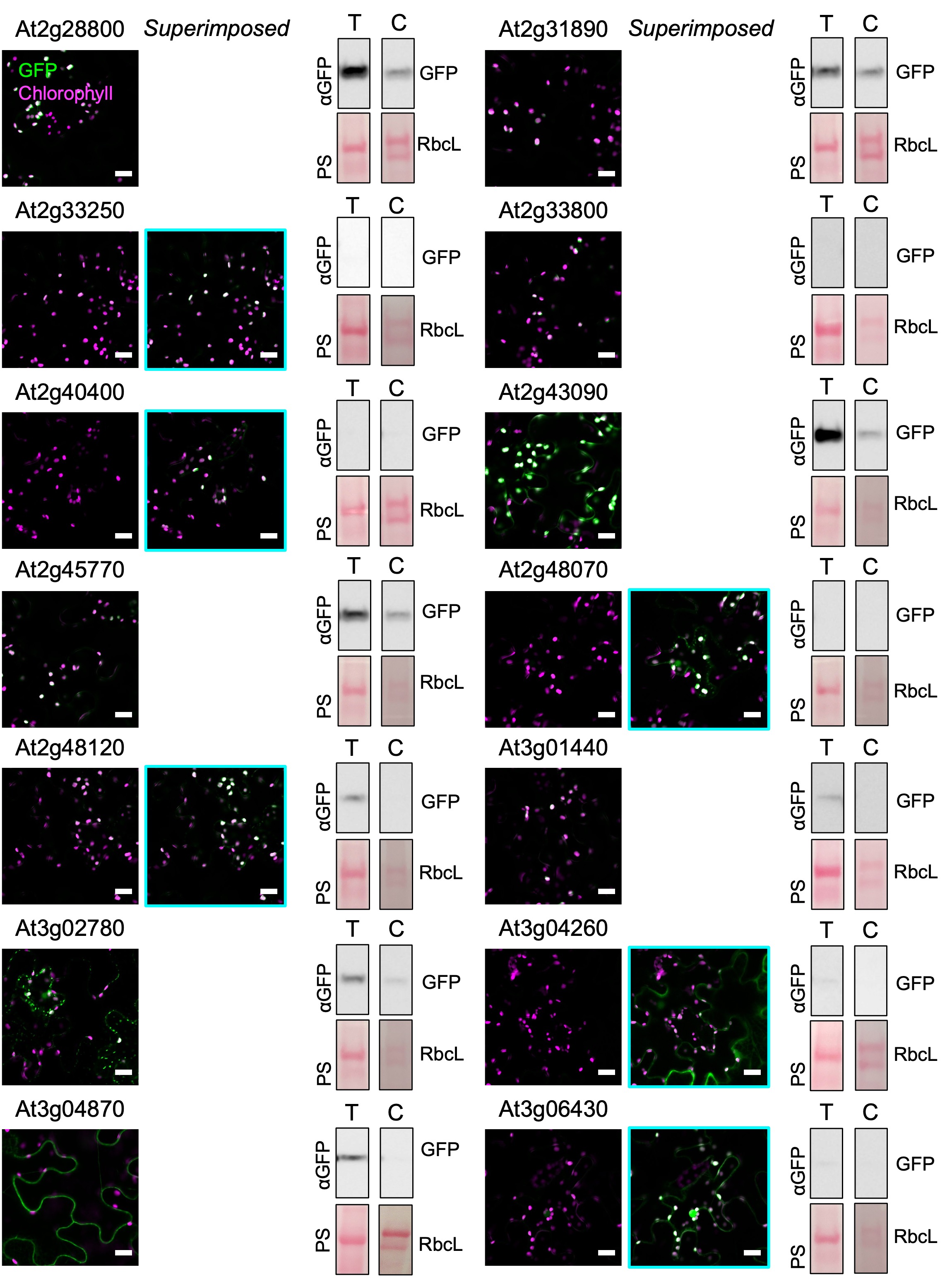

Supplement: S5 Fig — Subcellular localizations of different cTP-GFP(S65T) proteins in tobacco leaf cells at 4 days post agroinfiltration were observed under a confocal laser-scanning microscope (LSM 700). Scale bars = 20 μm. Immunoblot analysis of total leaf proteins (T) and isolated chloroplast proteins (C) showing the abundances of cTP-GFP(S65T) proteins in the cytosol and chloroplasts after translocation. αGFP = immunoblotted membranes probed by anti-GFP antibody. PS = Ponceau S-stained membrane to show the equal loading of protein samples. (JPG) [file pbio.3002785.s005.jpg]

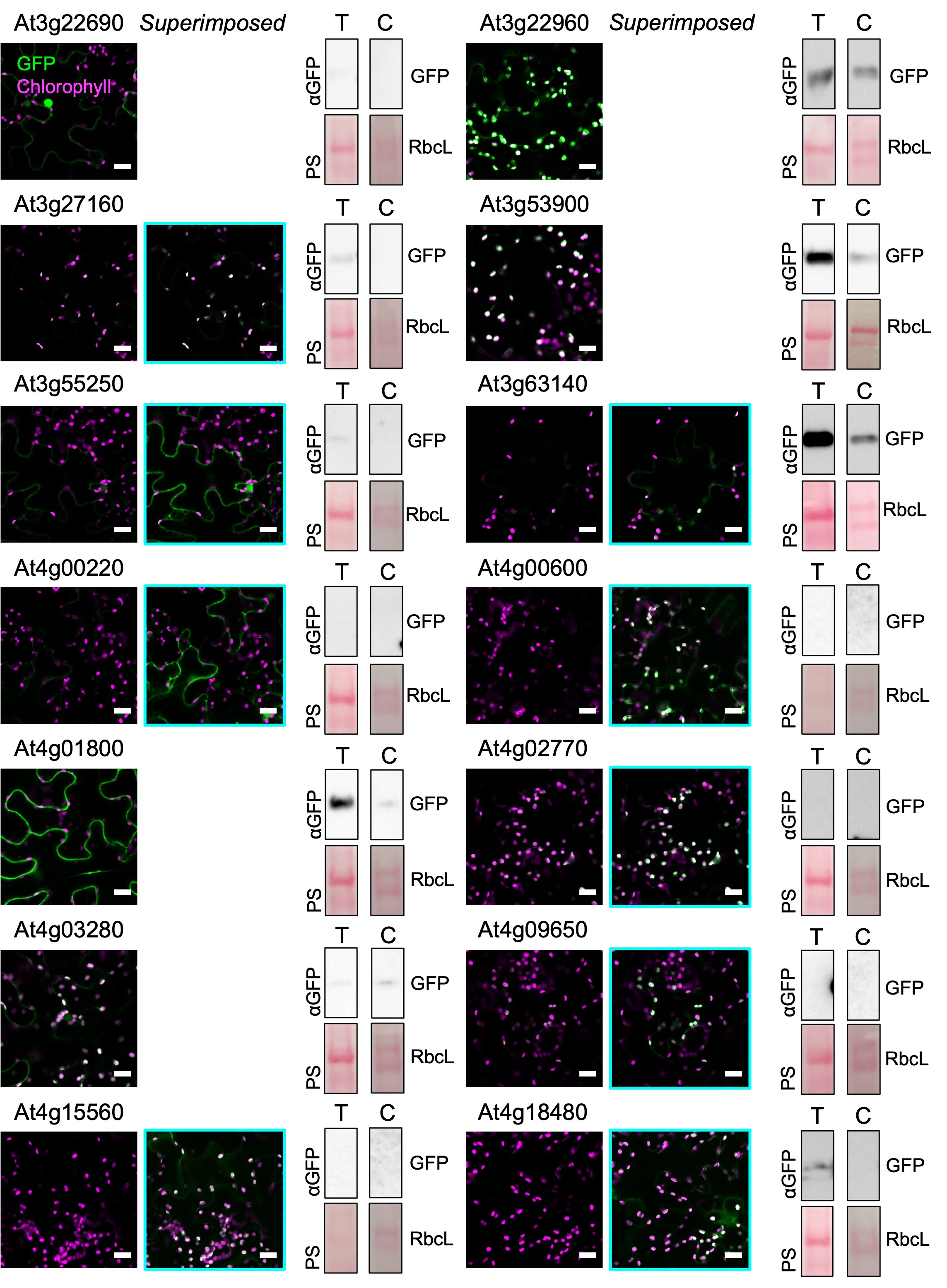

Supplement: S6 Fig — Subcellular localizations of different cTP-GFP(S65T) proteins in tobacco leaf cells at 4 days post agroinfiltration were observed under a confocal laser-scanning microscope (LSM 700). Scale bars = 20 μm. Immunoblot analysis of total leaf proteins (T) and isolated chloroplast proteins (C) showing the abundances of cTP-GFP(S65T) proteins in the cytosol and chloroplasts after translocation. αGFP = immunoblotted membranes probed by anti-GFP antibody. PS = Ponceau S-stained membrane to show the equal loading of protein samples. (JPG) [file pbio.3002785.s006.jpg]

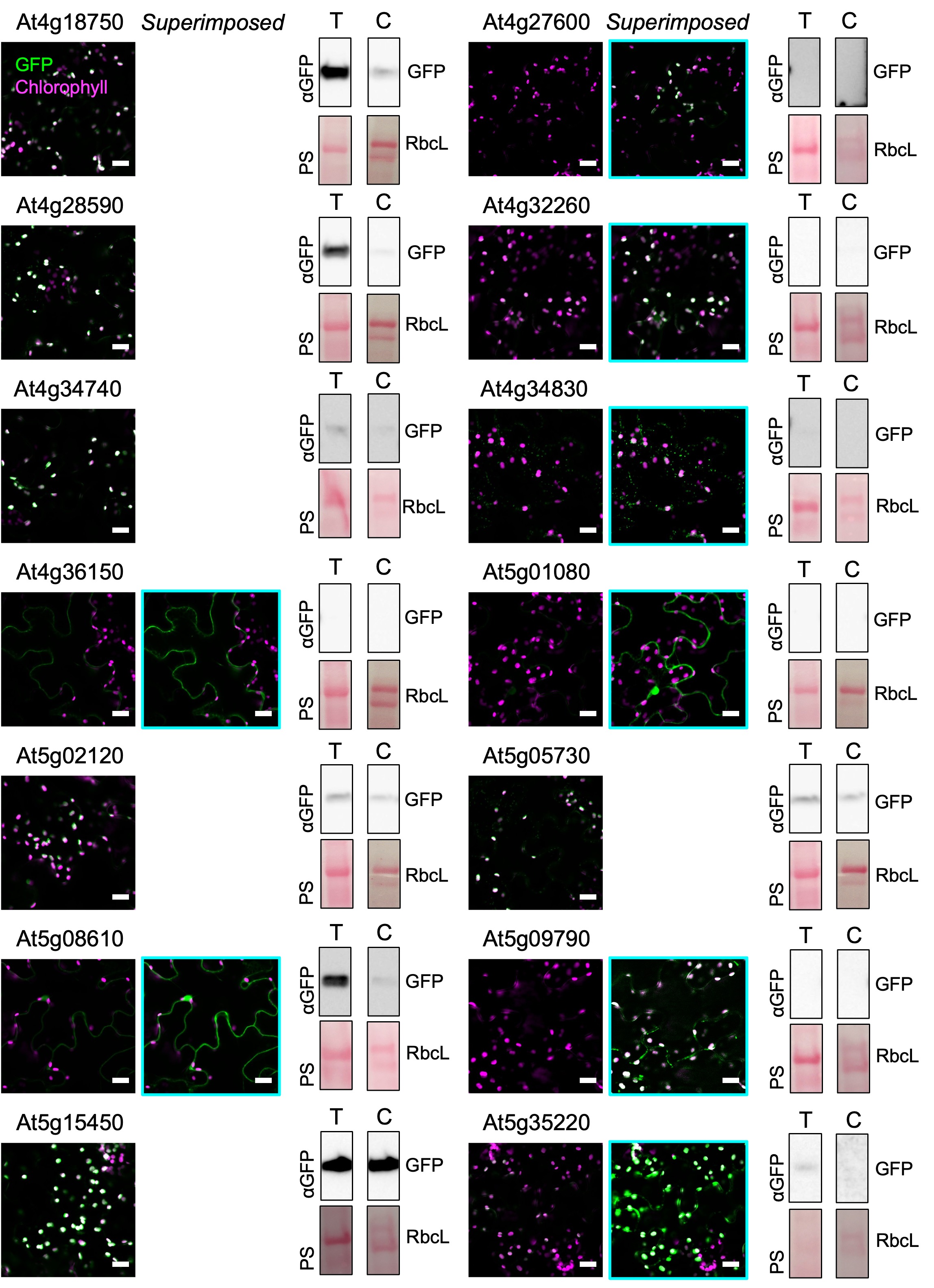

Supplement: S7 Fig — Subcellular localizations of different cTP-GFP(S65T) proteins in tobacco leaf cells at 4 days post agroinfiltration were observed under a confocal laser-scanning microscope (LSM 700). Scale bars = 20 μm. Immunoblot analysis of total leaf proteins (T) and isolated chloroplast proteins (C) showing the abundances of cTP-GFP(S65T) proteins in the cytosol and chloroplasts after translocation. αGFP = immunoblotted membranes probed by anti-GFP antibody. PS = Ponceau S-stained membrane to show the equal loading of protein samples. (JPG) [file pbio.3002785.s007.jpg]

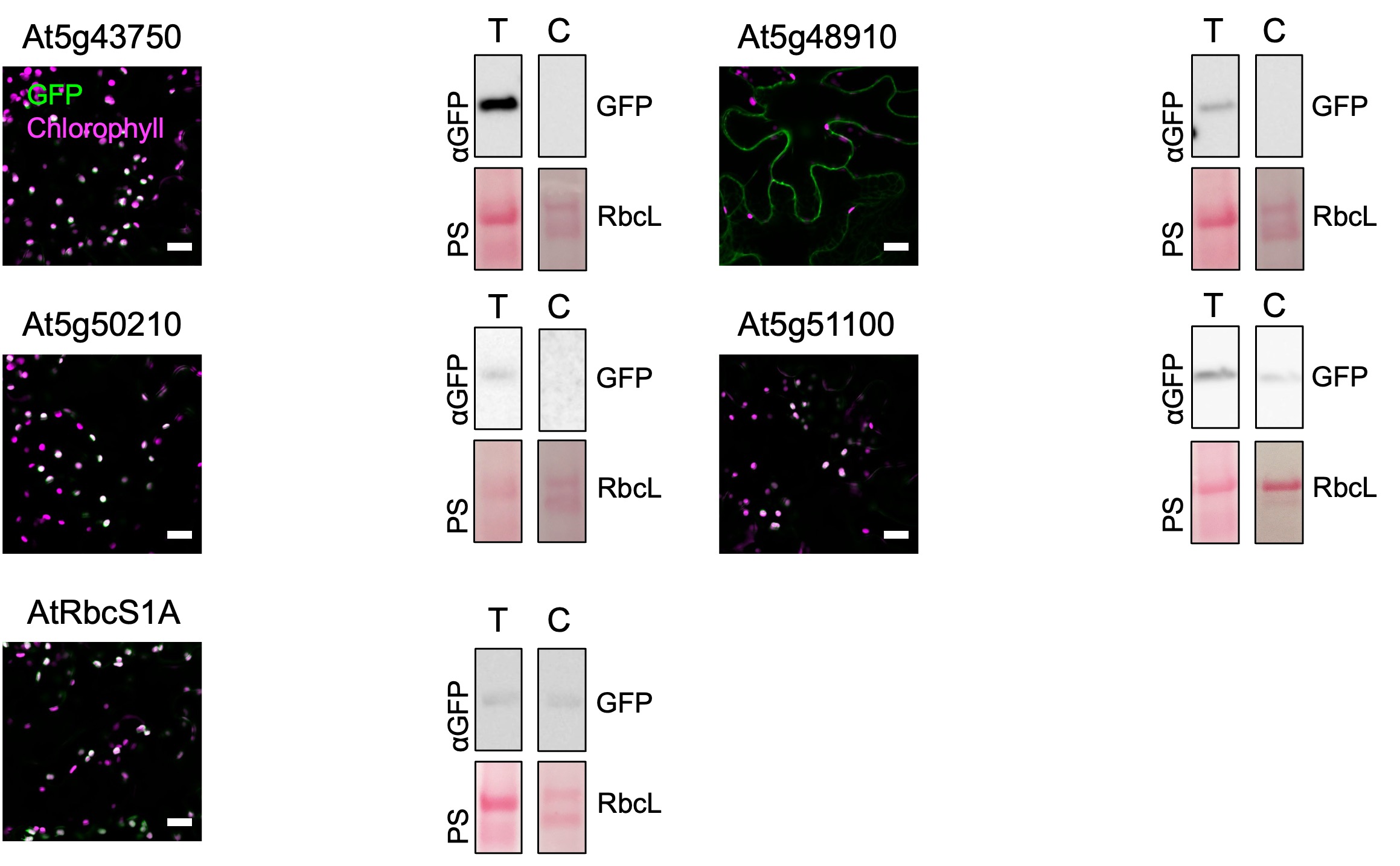

Supplement: S8 Fig — Subcellular localizations of different cTP-GFP(S65T) proteins in tobacco leaf cells at 4 days post agroinfiltration were observed under a confocal laser-scanning microscope (LSM 700). Scale bars = 20 μm. Immunoblot analysis of total leaf proteins (T) and isolated chloroplast proteins (C) showing the abundances of cTP-GFP(S65T) proteins in the cytosol and chloroplasts after translocation. αGFP = immunoblotted membranes probed by anti-GFP antibody. PS = Ponceau S-stained membrane to show the equal loading of protein samples. (JPG) [file pbio.3002785.s008.jpg]

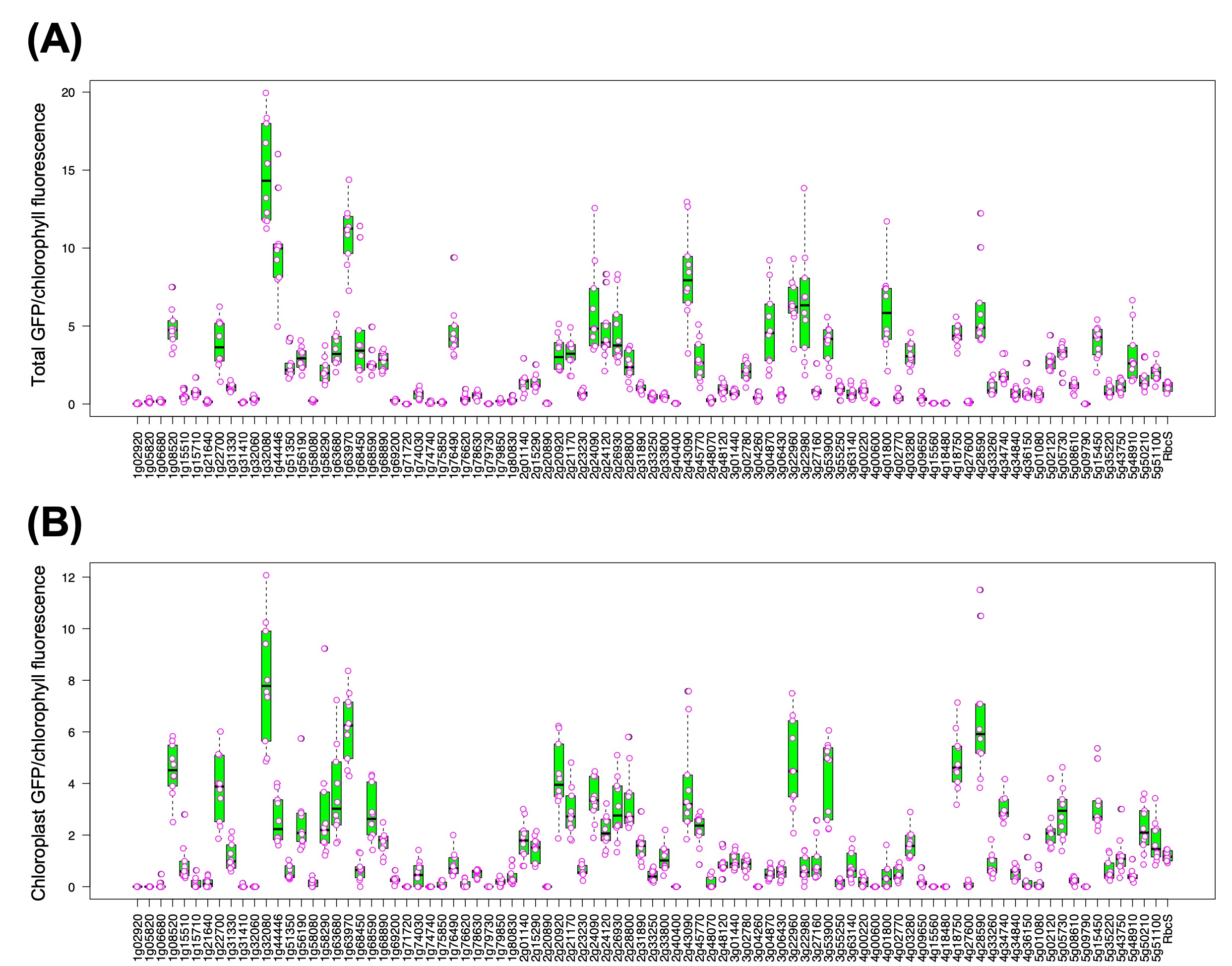

Supplement: S9 Fig — (A) Distribution of normalized GFP fluorescence in plant cells transiently expressing recombinant fluorescent protein. (B) Normalized GFP fluorescence in chloroplasts of plant cells transformed with 89 different cTP-GFP expression cassettes. Ten independent CLSM images (n = 10) were taken from 2 tobacco leaves from 2 independent experiments. GFP fluorescence in plant cells and chloroplasts and chlorophyll autofluorescence were quantified by Fiji ImageJ. Normalized fluorescence values in each treatment are presented as box plots. Magenta circles represent the distribution of data in the box plot. Black bars are medians. Fluorescence values in S9A and S9B Fig can be also found in S6 Data. (JPG) [file pbio.3002785.s009.jpg]

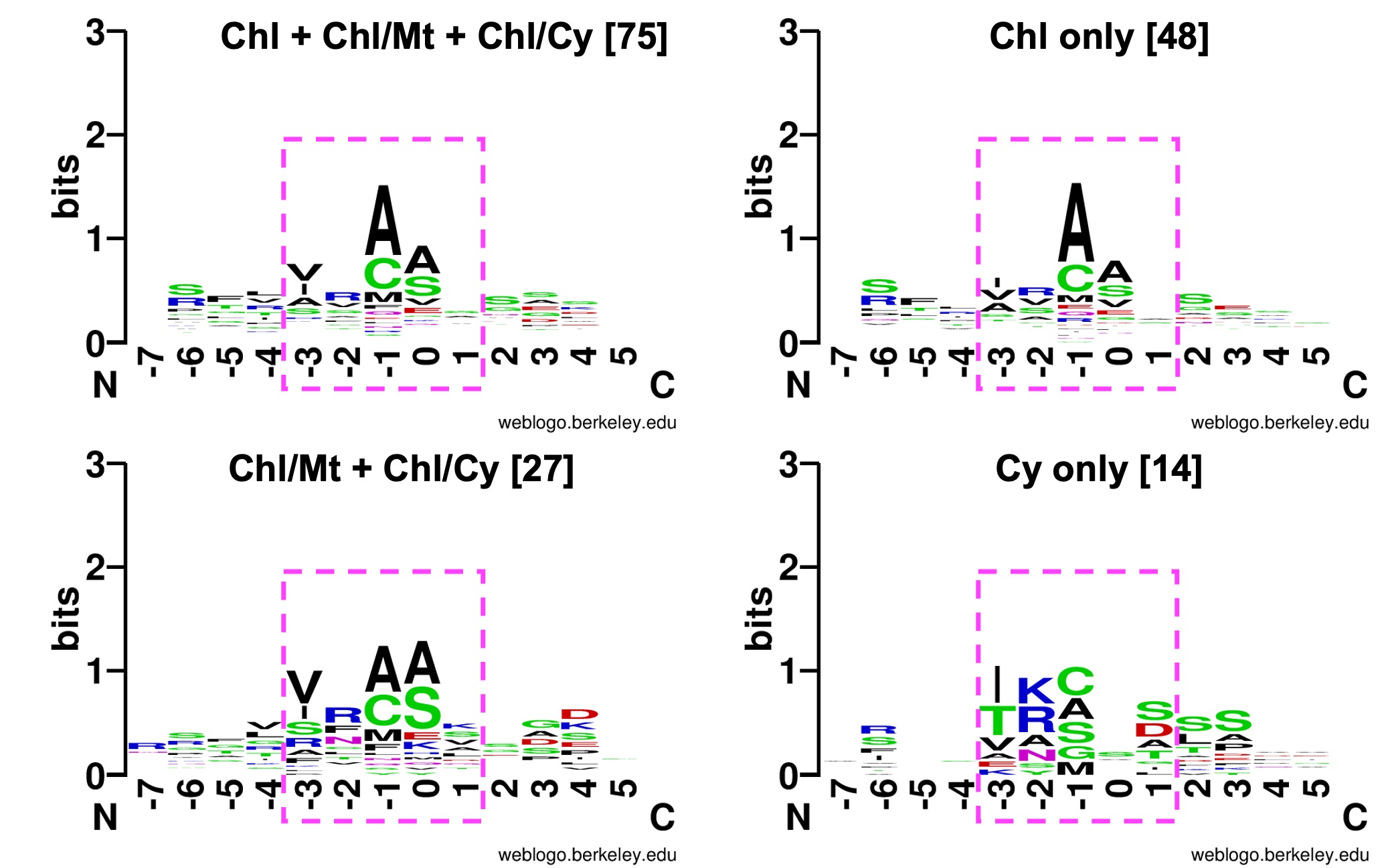

Supplement: S10 Fig — The alignment encompasses the cleavage sites of cTPs demonstrating chloroplast-specific targeting of GFP (Chl), dual-targeting cTPs that transport GFP to both chloroplasts and mitochondria (Chl/Mt) or to the cytosol (Chl/Cy), and the predicted cTPs that mislocalize GFP to the cytosol instead of the chloroplast (Cy). Multiple sequence alignments were conducted using WebLogo. The 5 amino acids within the magenta box at positions −3 to 1 represent the predicted cleavage site of the cTPs or tTPs, along with an additional 4 amino acids on both the N-terminus (−7 to −4) and C-terminus (2 to 5). The amino acid at position 0 corresponds to the cleaved residue, which constitutes the final amino acid attached to each transit peptide after cleavage. The number in the brackets denotes the count of cleavage sequences of the predicted cTPs in each group. (JPG) [file pbio.3002785.s010.jpg]

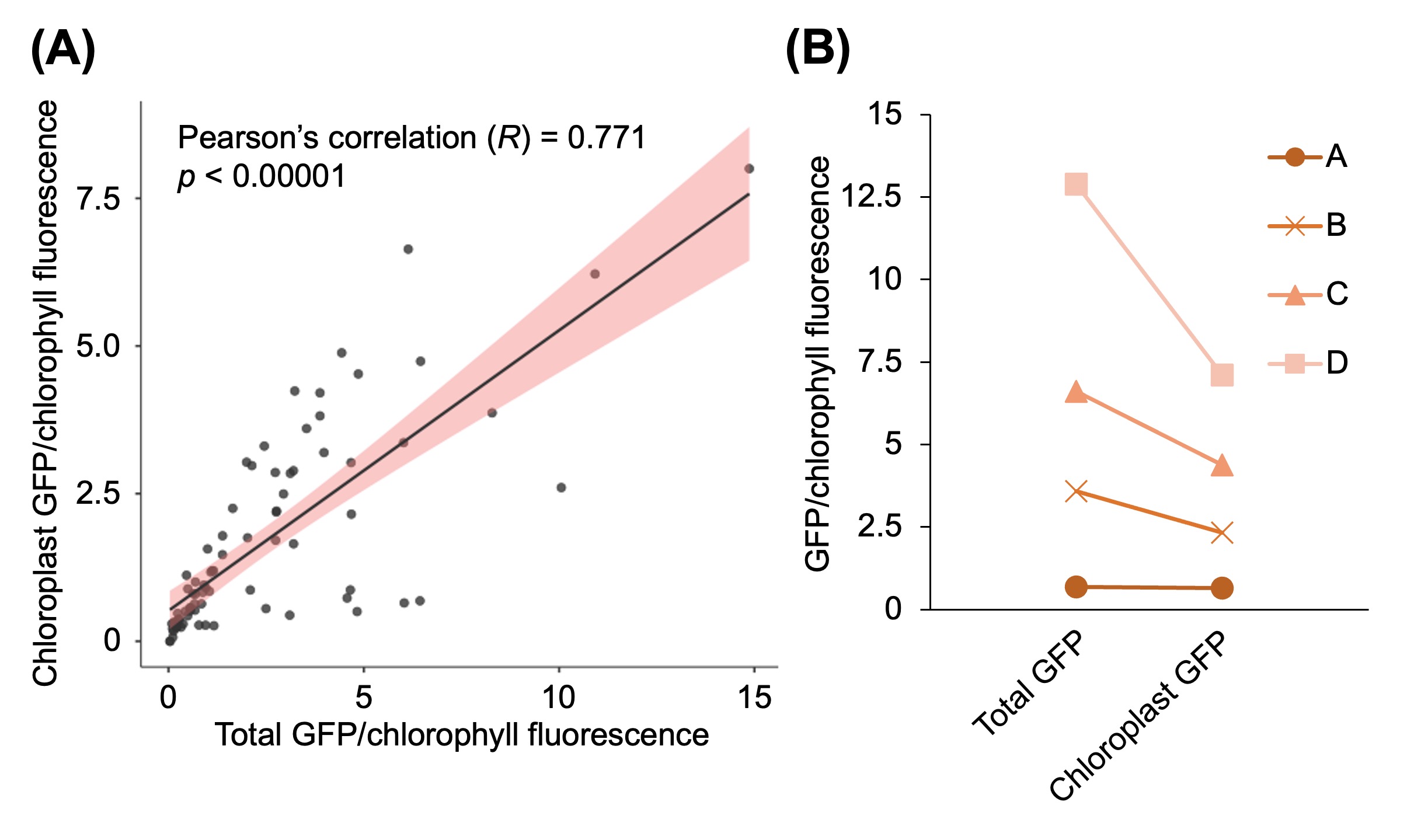

Supplement: S11 Fig — Recombinant cTP-GFP was transiently expressed in tobacco leaf cells by Agroinfiltration. CLSM imaging and fluorescence measurement were performed at 3 DAI. (A) Correlation between total GFP fluorescence in CLSM images of leaf cells and GFP signals inside the chloroplasts. GFP fluorescence in plant cells and chloroplasts was normalized by the respective chlorophyll autofluorescence in the image. (B) Eighty-nine cTP-GFP proteins were classified into 4 different clusters based on the correlation between total GFP in plant cells and GFP fluorescence in chloroplasts. Fluorescence values and classification of different cTP-GFPs are presented in S7 Data. (JPG) [file pbio.3002785.s011.jpg]

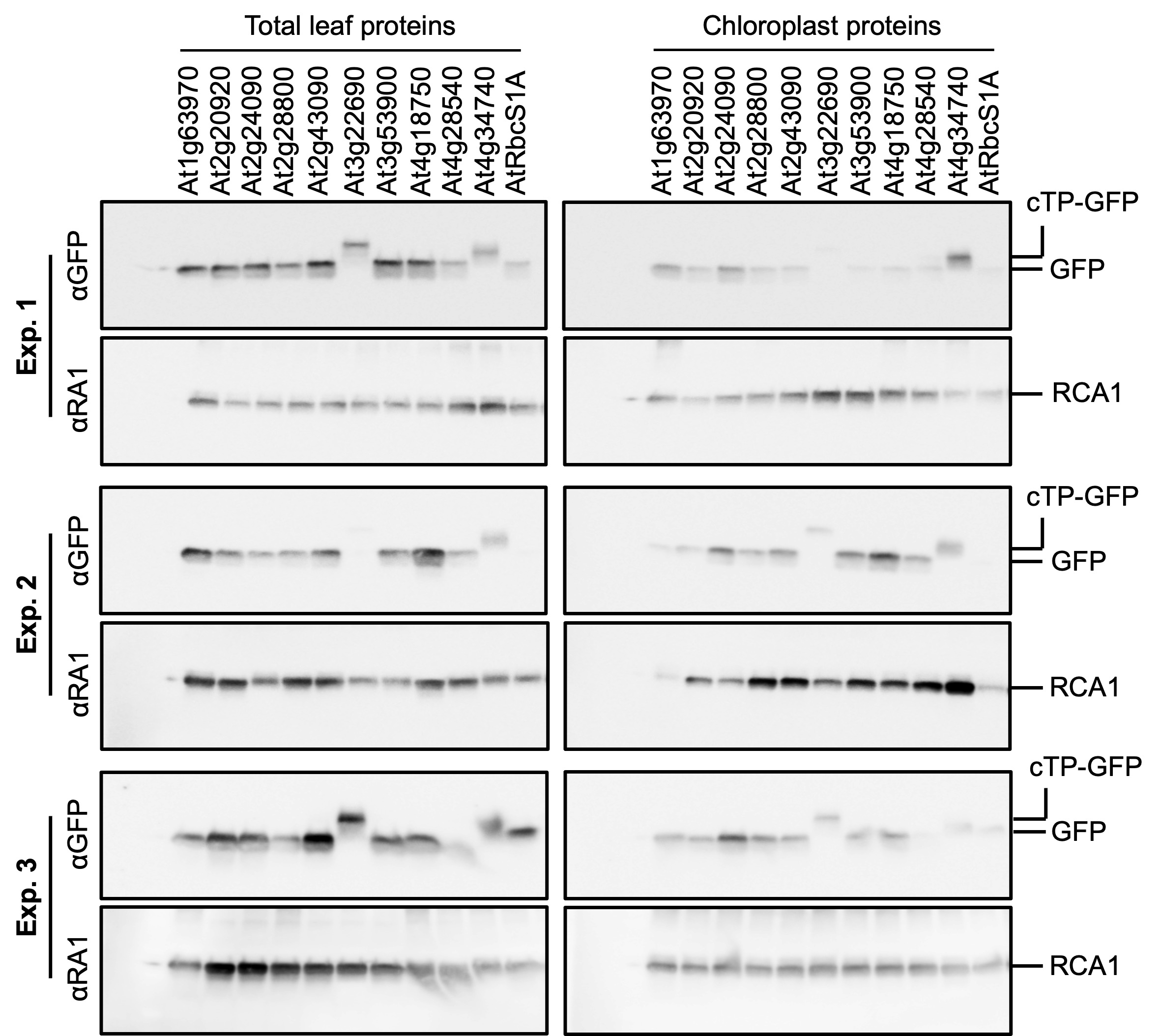

Supplement: S12 Fig — Differential accumulation of total leaf proteins and isolated chloroplast proteins in agroinfiltrated leaves was analyzed by SDS-PAGE and immunoblot analysis using anti-GFP (αGFP) and anti-RA1 (αRA1) antibodies. Three independent experiments were conducted. Band intensities of the target proteins of the respective sizes were quantified by Fiji ImageJ. Quantitative immunoblot results and representative images are shown in Fig 2. (JPG) [file pbio.3002785.s012.jpg]

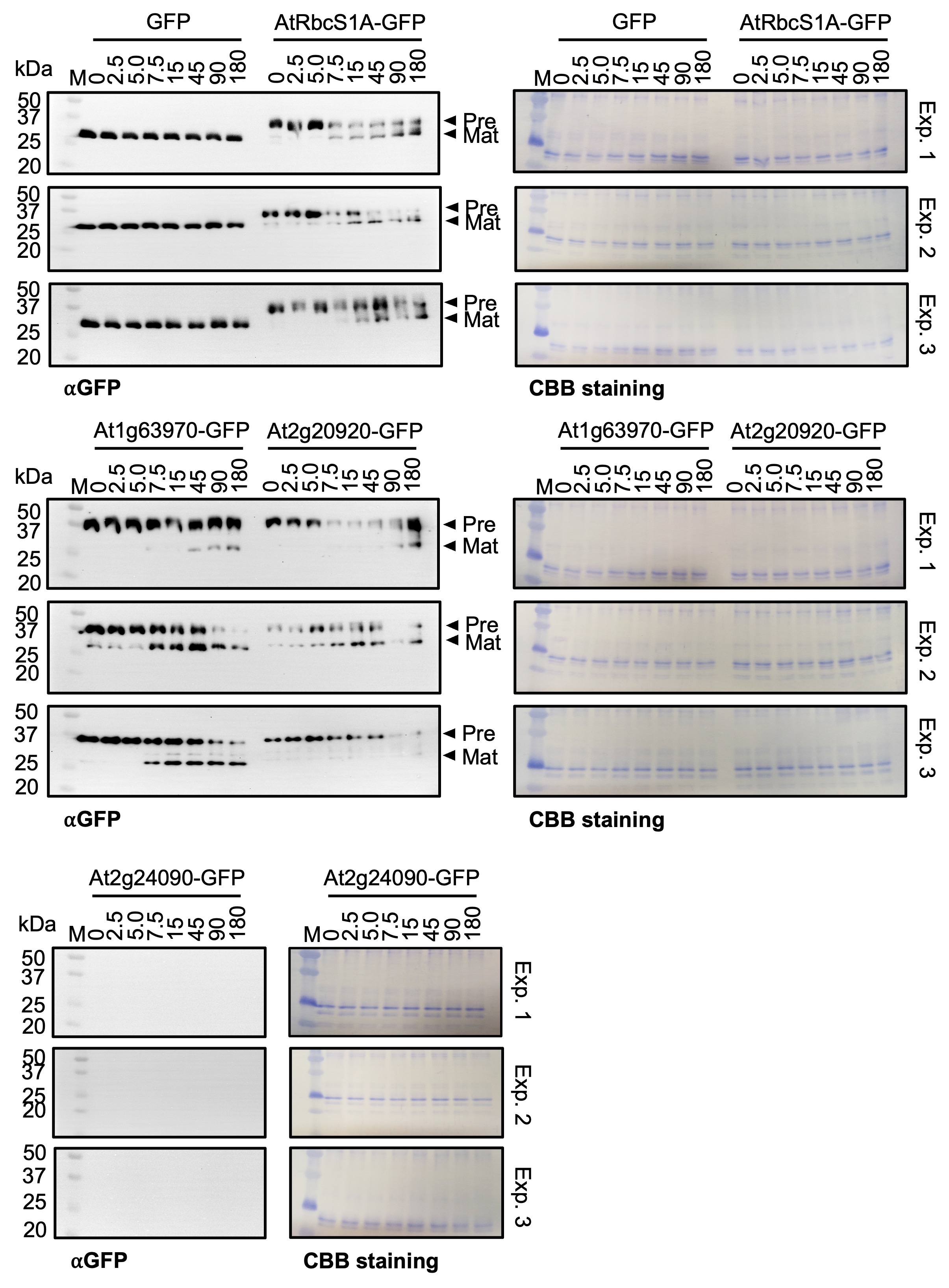

Supplement: S13 Fig — Purified recombinant cTP-GFPs (obtained from the E. coli expression system) were incubated with isolated tobacco chloroplasts. Import assay reactions were collected at various time points following incubation, and total proteins in the import reactions were separated using 6 M urea cracking solution. Subsequently, 2 μg protein samples were subjected to immunoblotting with anti-GFP antibody. After immunoblotting, the membranes were stained with CBB. The band intensities of the GFP-specific signal on the membrane were quantified using Fiji ImageJ. To ensure statistical validity, 3 independent import experiments were conducted. (JPG) [file pbio.3002785.s013.jpg]

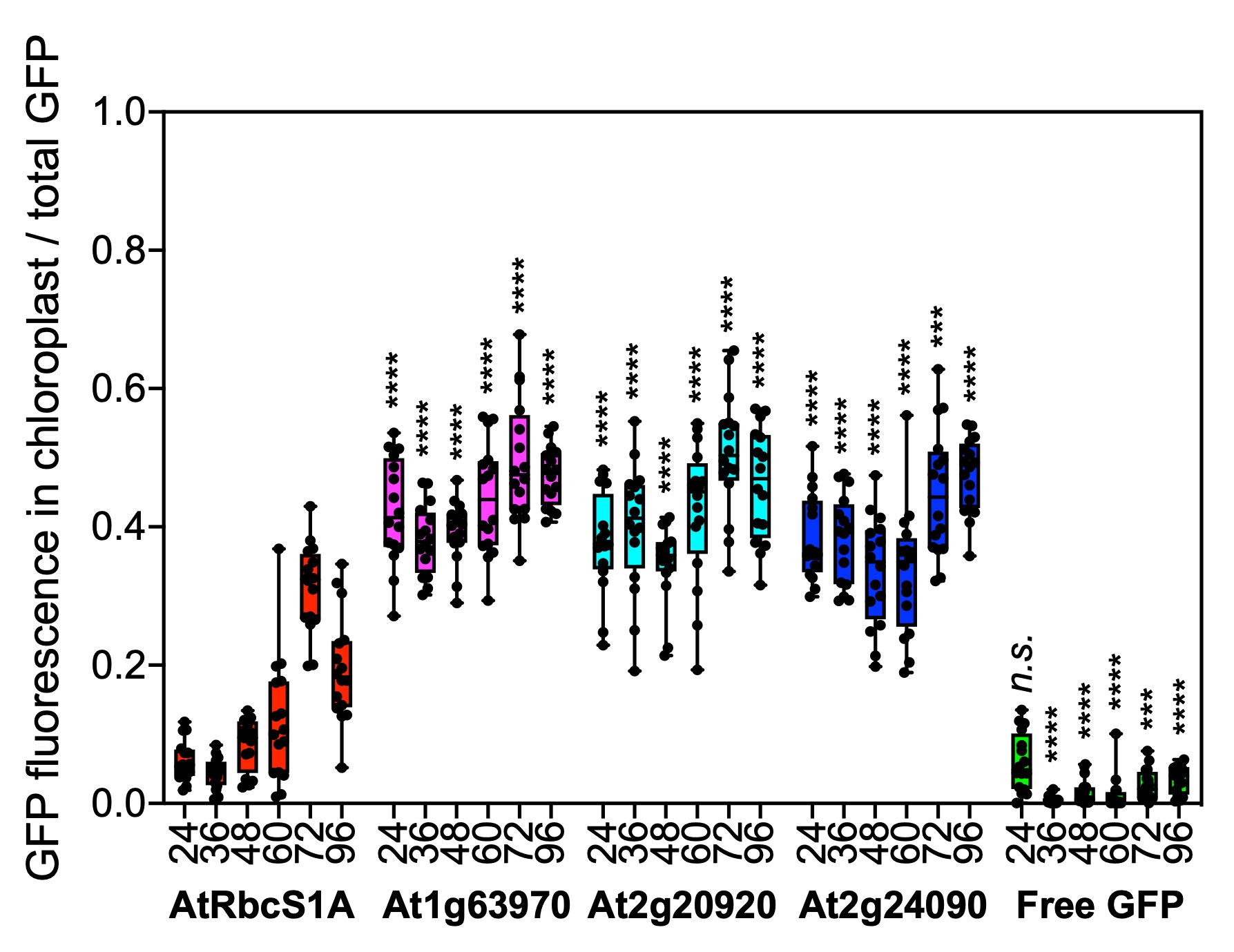

Supplement: S14 Fig — GFP fluorescence in plant cells and within chloroplasts was quantified using Fiji ImageJ in confocal laser scanning microscopy (CLSM) images of transfected tobacco leaf cells expressing different recombinant cTP-GFP constructs. The box plot illustrates the distribution of fluorescence ratios across 16 CLSM images (n = 16, S11 Data) collected from 3 biologically independent leaves at various time points post agroinfiltration. The central bars indicate the median fluorescence ratios at each time point, while the upper and lower bars depict the maximum and minimum values, respectively. Asterisks denote different levels of statistical significance in the mean fluorescence ratios at each time point compared to AtRbcS1A-GFP, used as a control (*; P ≤ 0.05, **; P ≤ 0.01, ***; P ≤ 0.001, and ****; P < 0.0001). “n.s.” indicates no significant difference in mean fluorescence ratio compared to the control. (JPG) [file pbio.3002785.s014.jpg]

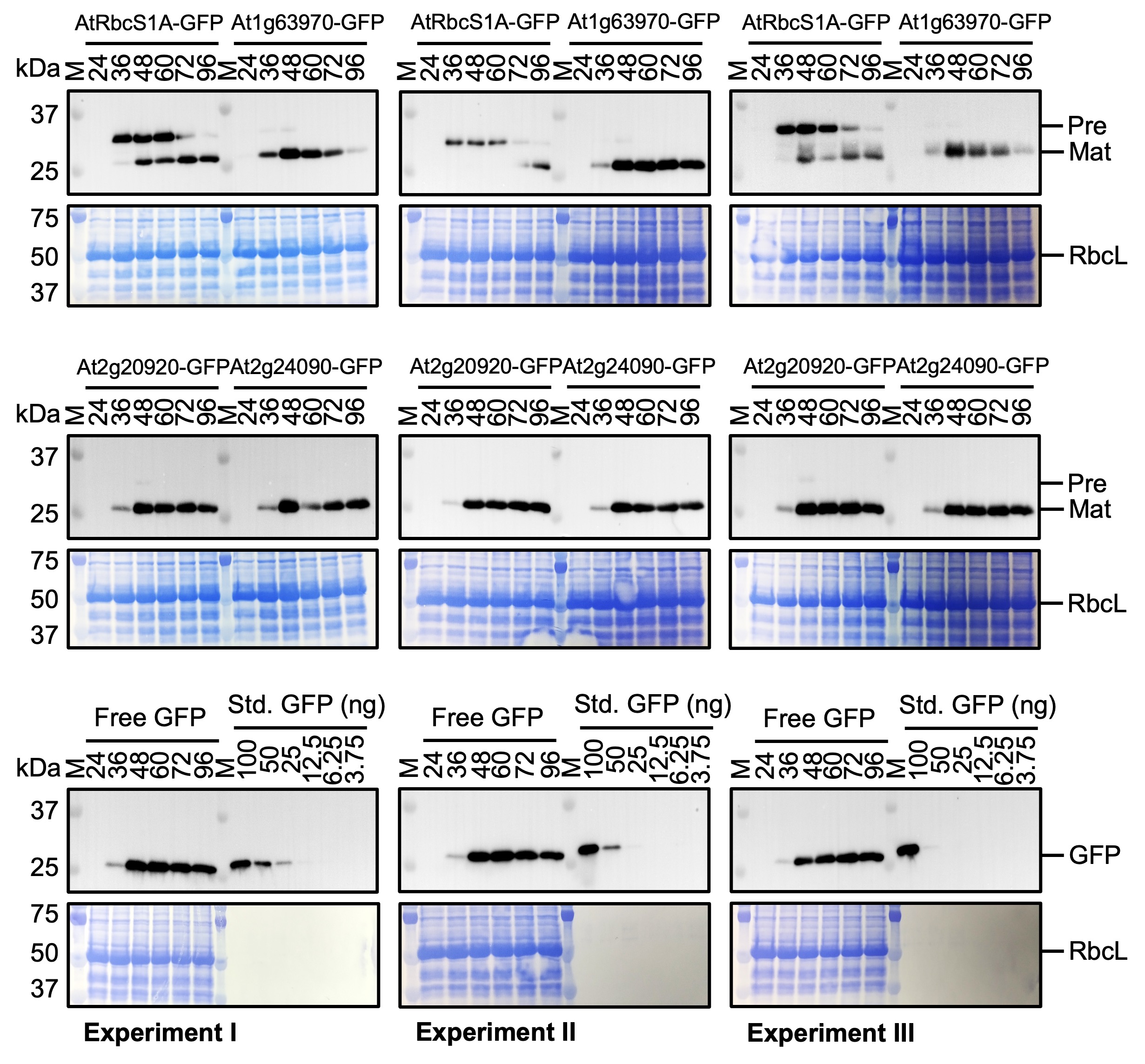

Supplement: S15 Fig — The transport of recombinant cTP-GFP to chloroplasts in transfected tobacco leaf cells was analyzed at 24, 36, 48, 60, 72, and 96 h after agroinfiltration using immunoblotting. The presence of various cTP-GFP precursors (Pre) and their chloroplast-localized cleaved products (Mat) in total leaf proteins was detected using an anti-GFP polyclonal antibody. The intensity of the GFP band in each sample was then compared to a linear regression equation generated using different amounts of standard GFP. nnL indicates the major Rubisco large subunit protein band on the immunoblot membrane stained with CBB. (JPG) [file pbio.3002785.s015.jpg]

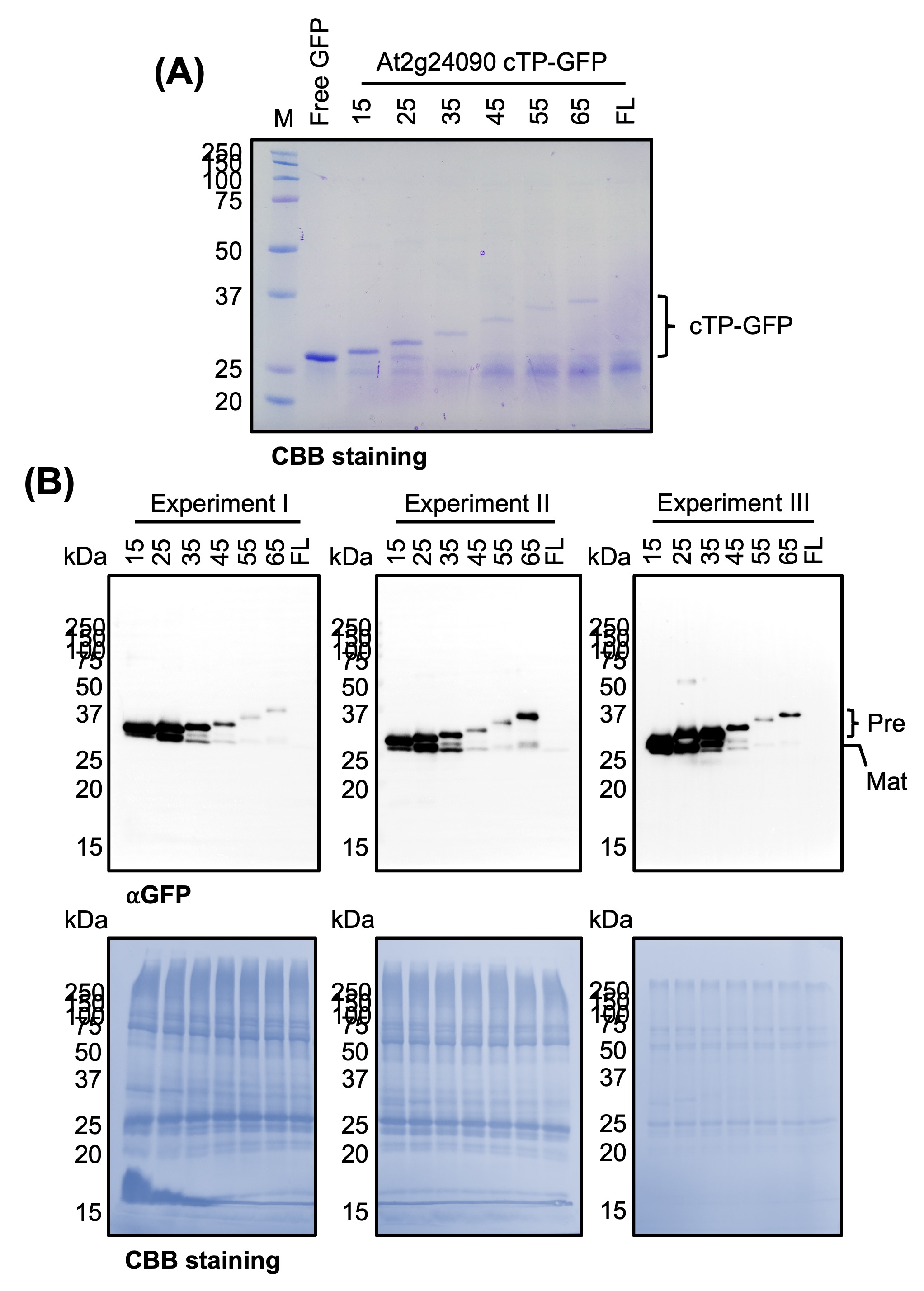

Supplement: S16 Fig — (A) SDS-PAGE of various truncated At2g24090 cTP-GFP fusion proteins. Recombinant cTP-GFPs were produced using the E. coli expression system, and 500 ng processed recombinant protein samples were analyzed using a 14% SDS-PAGE gel. The gel was stained with CBB to visualize the protein bands. (B) In vitro import assays of various truncated At2g24090 cTP-GFP fusion proteins into isolated tobacco chloroplasts. Each 100 μm recombinant protein sample was incubated with isolated chloroplasts for 3 h. Following incubation, the import reaction was halted by adding EDTA to a final concentration of 50 mM. Total proteins in the import reaction were separated using 6 M urea cracking solution, and 2 μg of protein was subjected to immunoblotting using an anti-GFP antibody. After immunoblotting, the membranes were stained with CBB. Three independent analyses were conducted to ensure statistical validity. (JPG) [file pbio.3002785.s016.jpg]

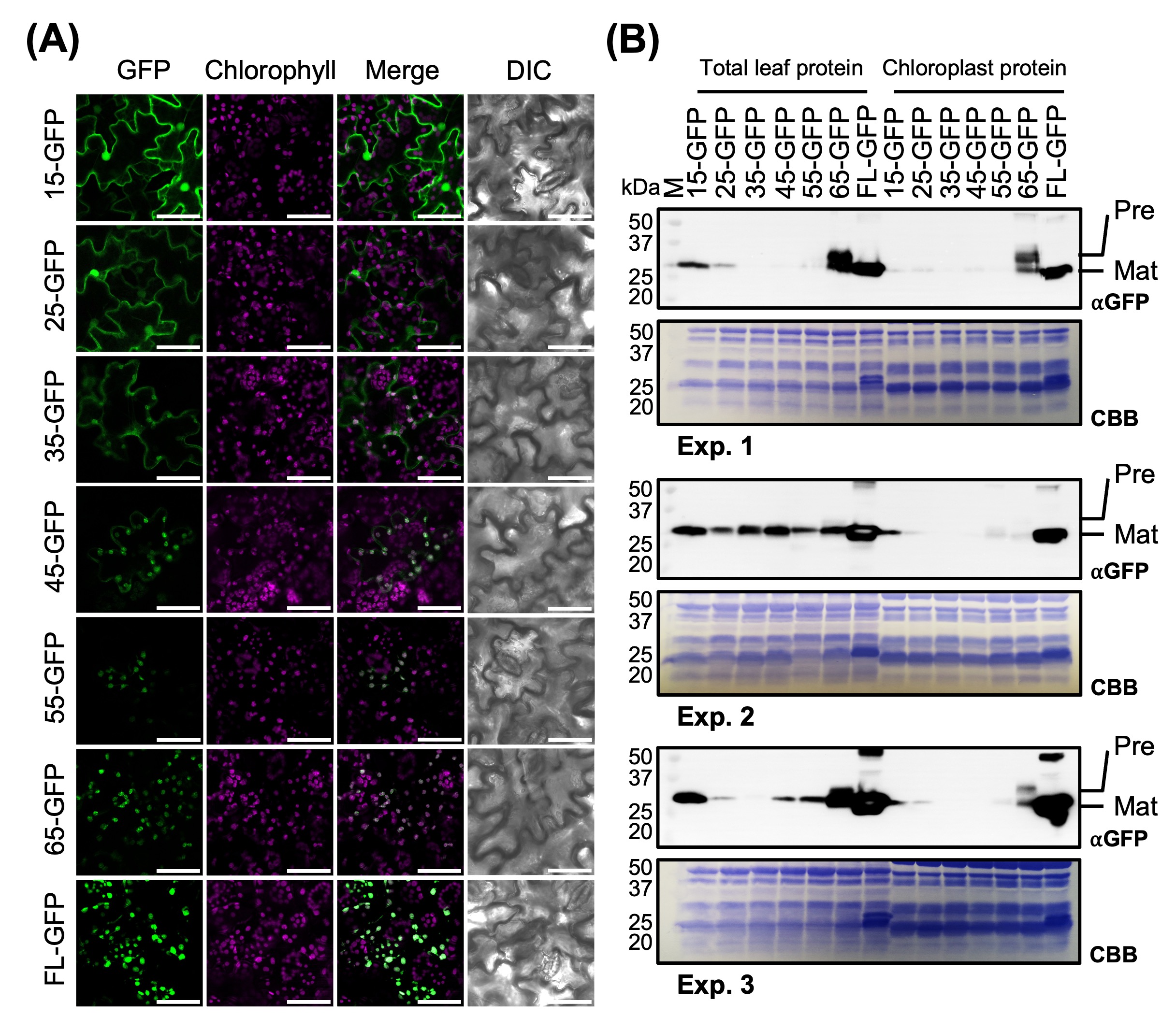

Supplement: S17 Fig — (A) Subcellular localizations of different truncated At2g24090 cTP-GFPs in tobacco leaf cells at 3 DAI. Scale bars = 50 μm. (B) Immunoblot analysis of total leaf proteins and isolated chloroplast proteins from tobacco leaves using anti-GFP antibody. (JPG) [file pbio.3002785.s017.jpg]

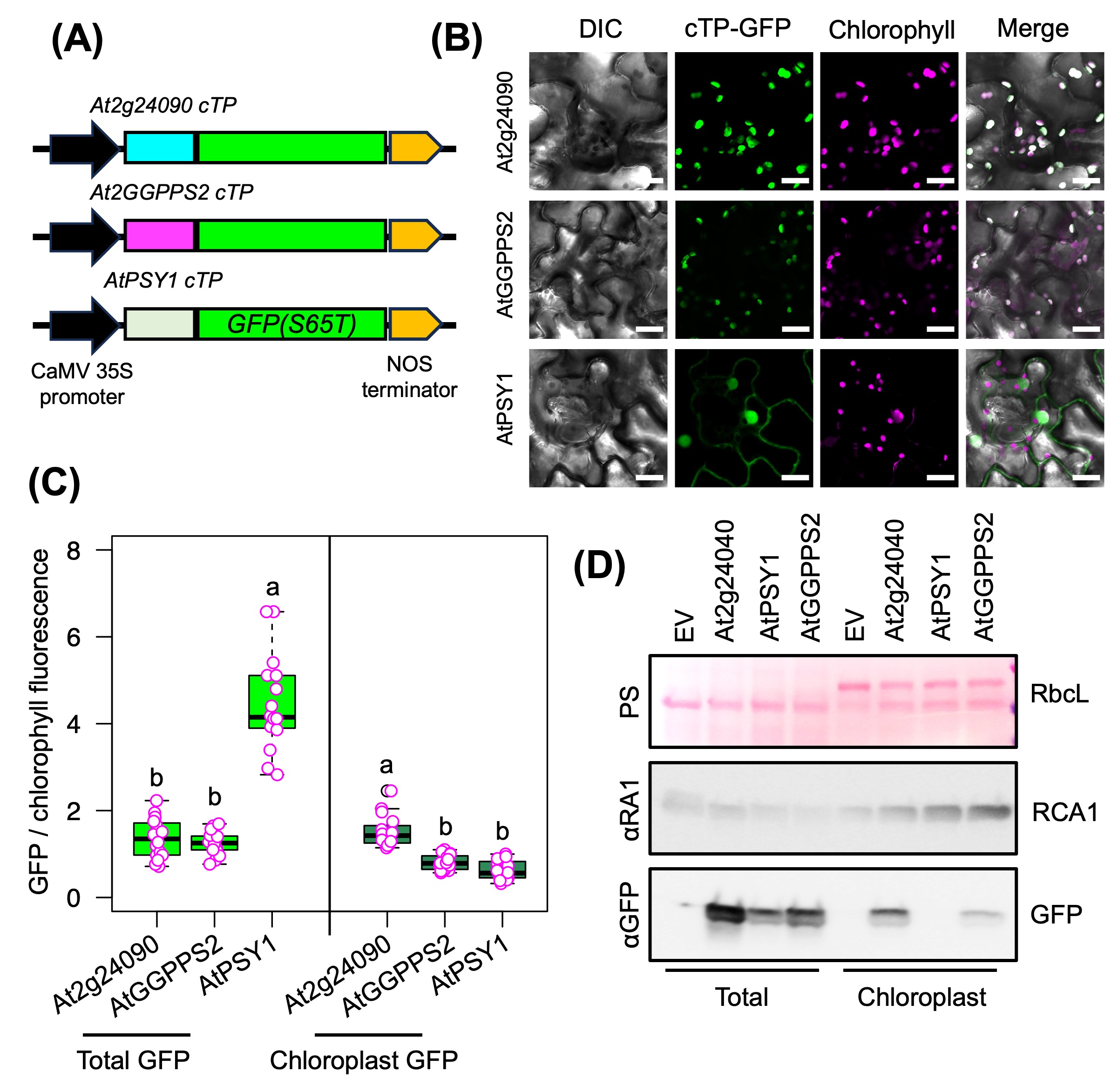

Supplement: S18 Fig — (A) Expression cassettes of recombinant cTP-GFPs in plant cells. The T-DNA vectors harboring these gene expression cassettes were introduced into tobacco leaf cells via Agroinfiltration. (B) Subcellular localization of different cTP-GFPs in tobacco leaf cells at 3 DAI. Scale bars = 20 μm. (C) Comparative analysis of cTP-GFP fluorescence in CLSM images. Distribution of GFP/chlorophyll fluorescence values in 16 ROIs from 4 different leaves is presented as a box plot (see values in S13 Data). Magenta dots represent each data point. Black bars are medians. Different letters indicate significant differences in the means among treatments (one-way ANOVA with Tukey’s HSD test at p = 0.00001). (D) Immunoblot analysis of total leaf proteins and isolated chloroplast proteins from tobacco leaves infiltrated with different cTP-GFP expression vectors. RbcL bands indicate equal loading of proteins on the membrane after Ponceau S staining (PS) before immunoblotting with anti-RA1 and anti-GFP antibodies. EV = proteins from plant leaves infiltrated with Agrobacterium harboring pBI121-empty vector. (JPG) [file pbio.3002785.s018.jpg]

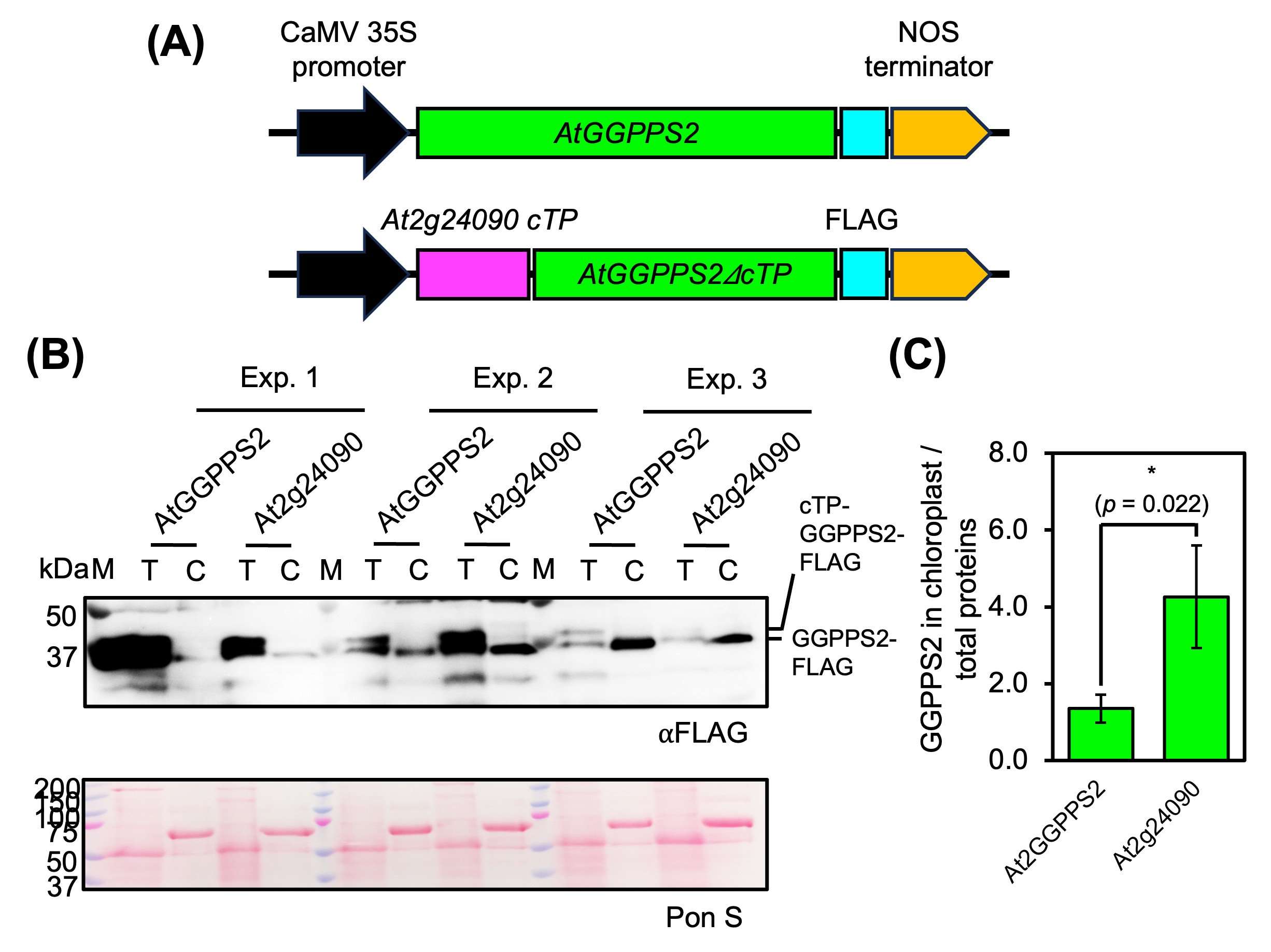

Supplement: S19 Fig — (A) Expression constructs used to overexpress recombinant cTP-modified AtGGPPS2 enzymes in plants. The coding sequence of native AtGGPPS2, including its cTP portion, was fused with a FLAG tag at the C-terminus for immunoblot analysis after expression in tobacco leaf cells via Agroinfiltration. Subsequently, the cTP portion was replaced by At2g24090 cTP to potentially enhance the import efficiency of the recombinant At2g24090 cTP-GGPPS2-FLAG enzyme into the chloroplasts. (B) Immunoblot analysis of total leaf proteins (T) and isolated chloroplast proteins (C) in agroinfiltrated tobacco leaves. Proteins were collected from 3 biologically independent samples and stored at –80°C. These proteins were subjected to immunoblotting using anti-FLAG antibody. The intensity of protein bands corresponding to the expected recombinant enzymes was determined using Fiji ImageJ. The membrane was stained with Ponceau S solution to ensure the equal loading of protein samples onto the membrane. (C) Accumulation of recombinant cTP-engineered GGPPS2-FLAG enzymes in chloroplasts analyzed by immunoblotting. An asterisk indicates a significant difference in the translocation of recombinant enzymes into chloroplasts between AtGGPPS2 cTP and At2g24090 cTP (Student’s t test: p < 0.05, n = 3, see values in S14 Data). (JPG) [file pbio.3002785.s019.jpg]

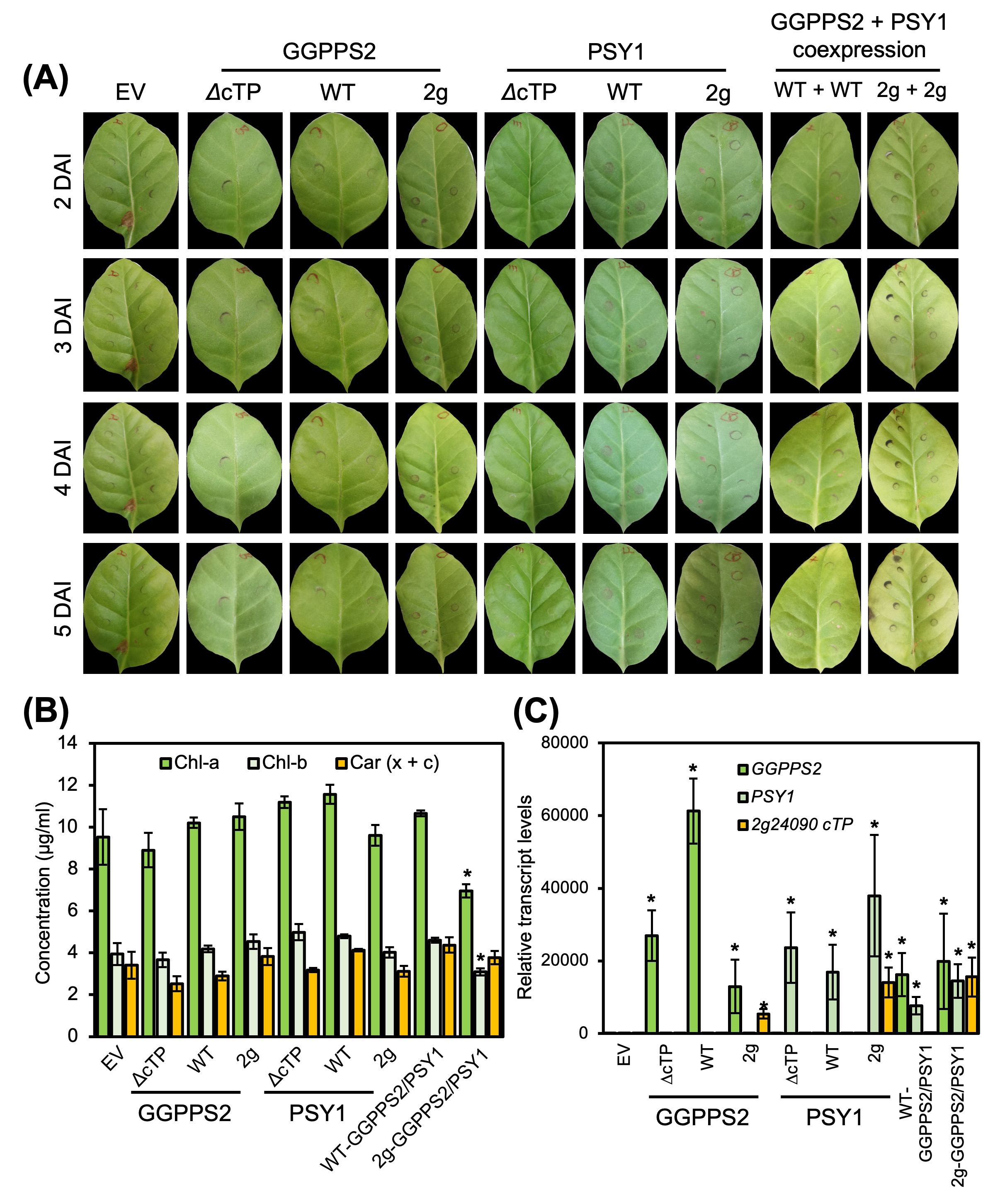

Supplement: S20 Fig — (A) Changes in the color of agroinfiltrated tobacco leaves after incubation. Fully expanded tobacco leaves were infiltrated with Agrobacterium containing different plant expression vectors. (B) Pigment concentrations in agroinfiltrated leaves at 5 DAI. Leaf pigments in the aqueous acetone fractions were analyzed by spectrophotometry. (C) Transcript levels of transgenes in agroinfiltrated tobacco leaves at 5 DAI. EV = empty vector, ΔcTP = cTP-depleted recombinant proteins, WT = native proteins, 2g = At2g24090 cTP-engineered proteins. Coexpression of native- or At2g24090 cTP-engineered enzymes is indicated by WT-GGPPS2/PSY1 or 2g-GGPPS2/PSY1, respectively. Error bars = standard deviations (n = 3). Significant differences in the means compared to EV are indicated by asterisks (Student’s t test at p < 0.05). Numerical values for S20B and S20C Figs can be found in S15 Data. (JPG) [file pbio.3002785.s020.jpg]

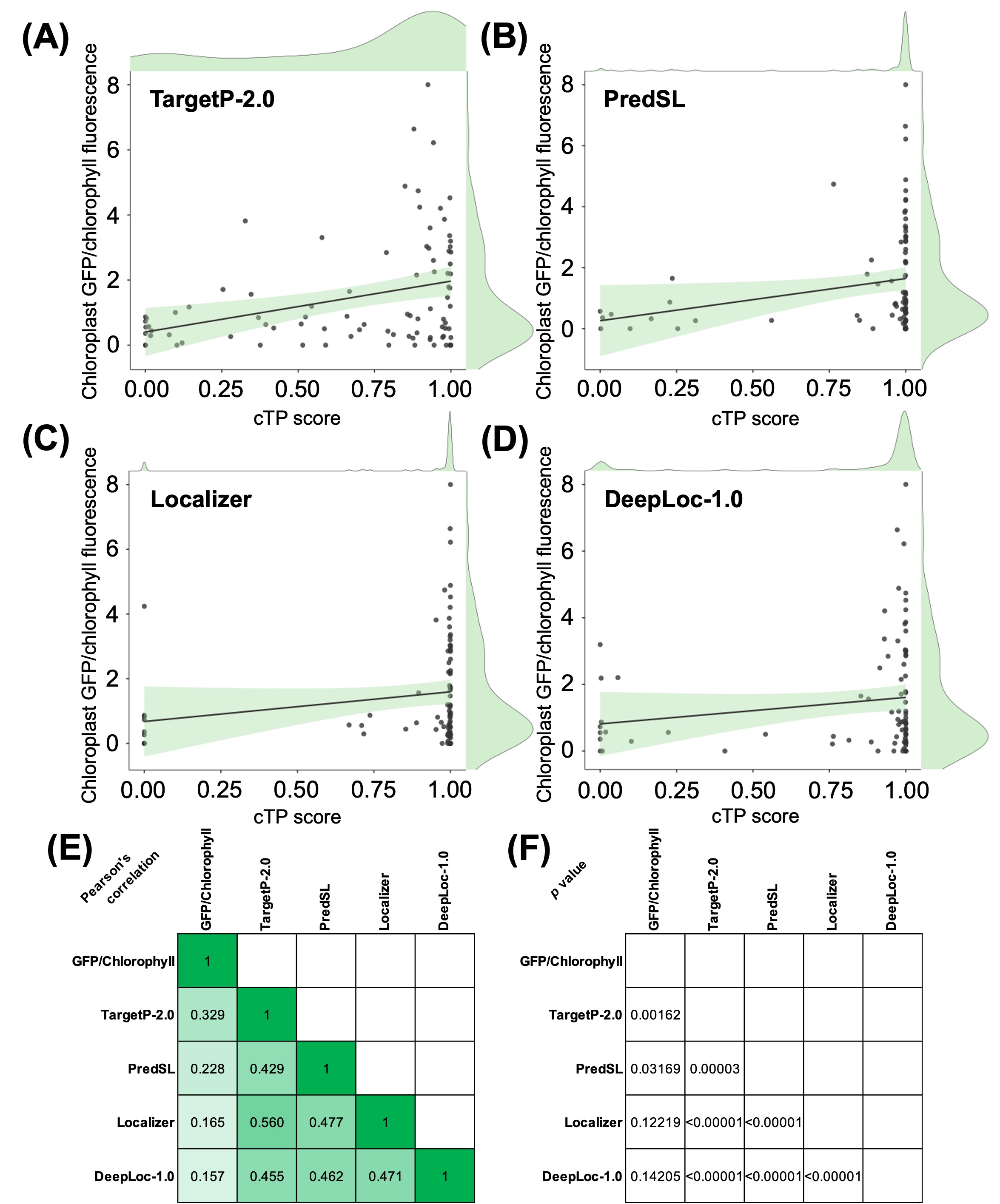

Supplement: S21 Fig — Correlations between cTP scores predicted by TargetP-2.0 (A), PredSL (B), Localizer (C), and DeepLoc-1.0 (D) and chloroplast import efficiencies of 89 transit peptide-GFP proteins (analyzed based on fluorescence measurements in CLSM images of plant cells overexpressing different cTP-GFPs) determined by linear regression. Distributions of chloroplast GFP/chlorophyll fluorescence and cTP scores are shown as density plots in (A–D). Shaded areas in the scatter plots represent standard error. The cTP scores of each preprotein from different predictions are shown in S18 and S19 Data. (E) Pearson’s correlations between cTP scores from each computational predictor and GFP/chlorophyll fluorescence. (F) Statistical significances of Pearson’s correlations in (E). (JPG) [file pbio.3002785.s021.jpg]

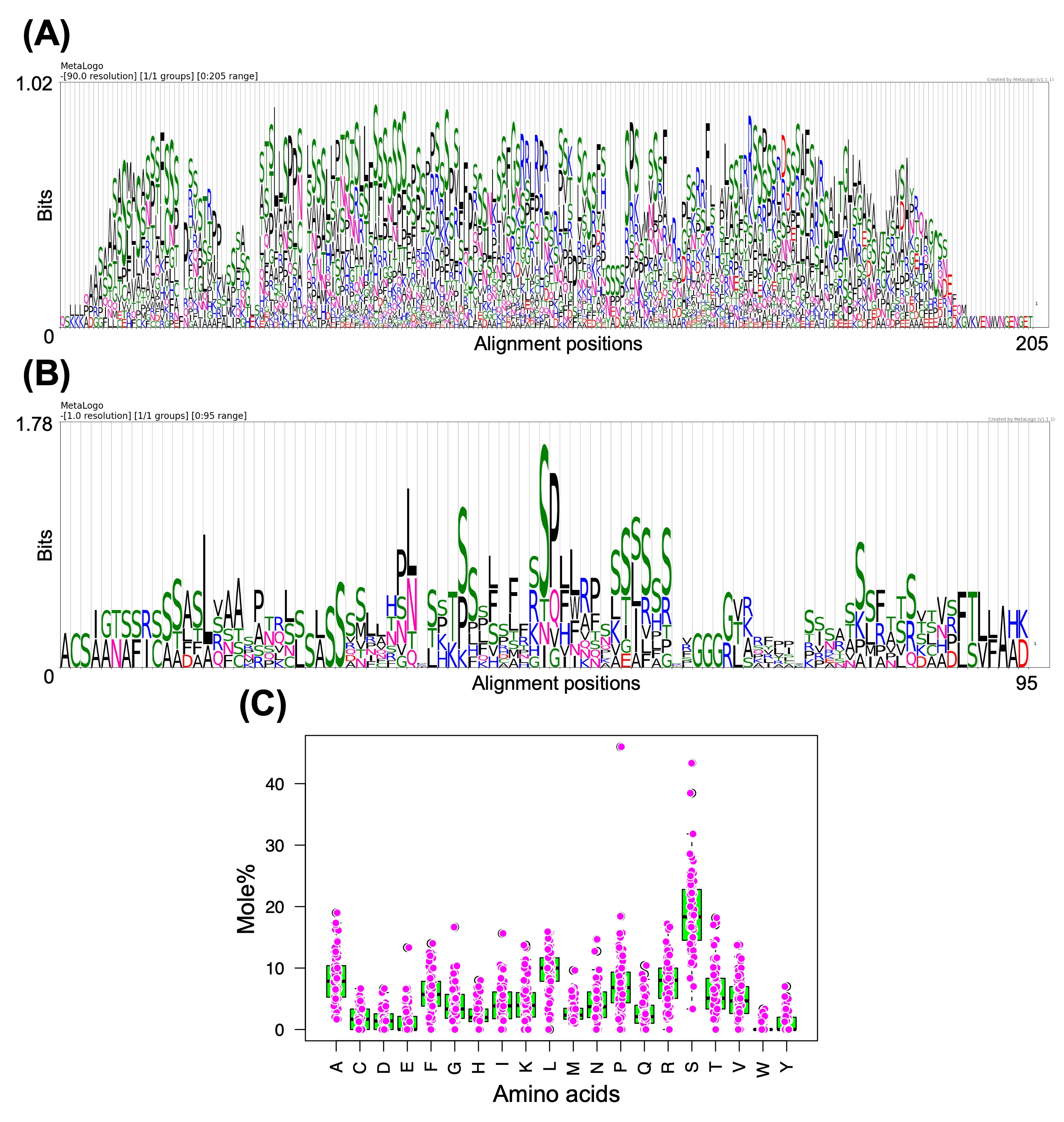

Supplement: S22 Fig — (A) MetaLogo alignment of all 89 amino acid sequences. (B) Multiple sequence alignment of 10 selected cTPs for comparative analysis. (C) Distribution of natural amino acids in all 89 polypeptides shown as a box plot. Black bar indicates the median of the distributed values. Dots represent data points of amino acid composition in each cTP as in S20 Data. (JPG) [file pbio.3002785.s022.jpg]
