# Supplementary material for: Identification of a highly efficient chloroplast-targeting peptide for plastid engineering
Source: PLoS Biol. 2024 Sep 19;22(9):e3002785. doi: 10.1371/journal.pbio.3002785 (PMC11444414; doi:10.1371/journal.pbio.3002785)
Supplement: S1 Raw images — (PDF) [file pbio.3002785.s046.pdf]

Original blot images for S2-8 Figs.

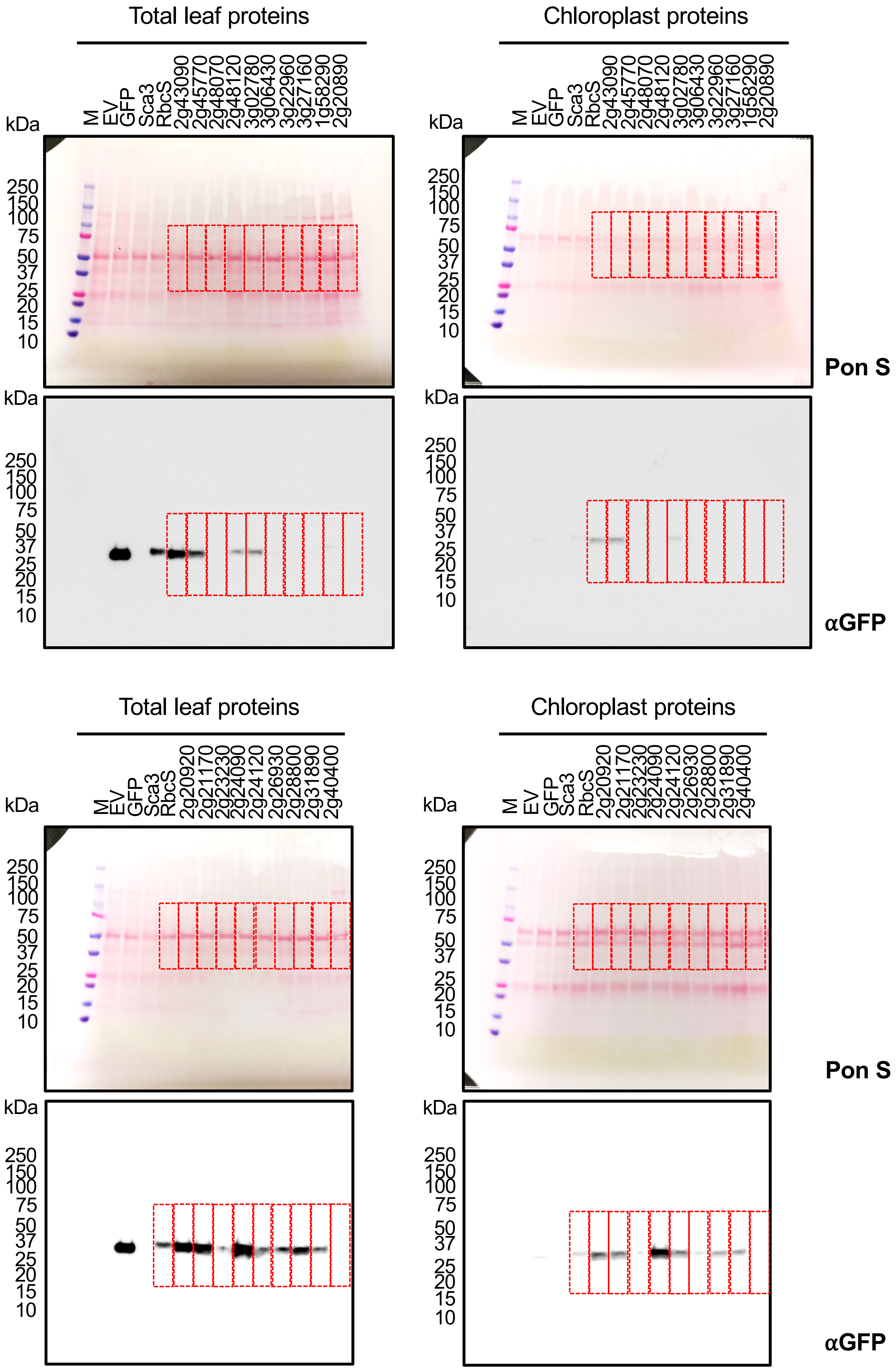

Note ; Red rectangles show cropped images in representative figures.

Original blot images for S2-8 Figs.

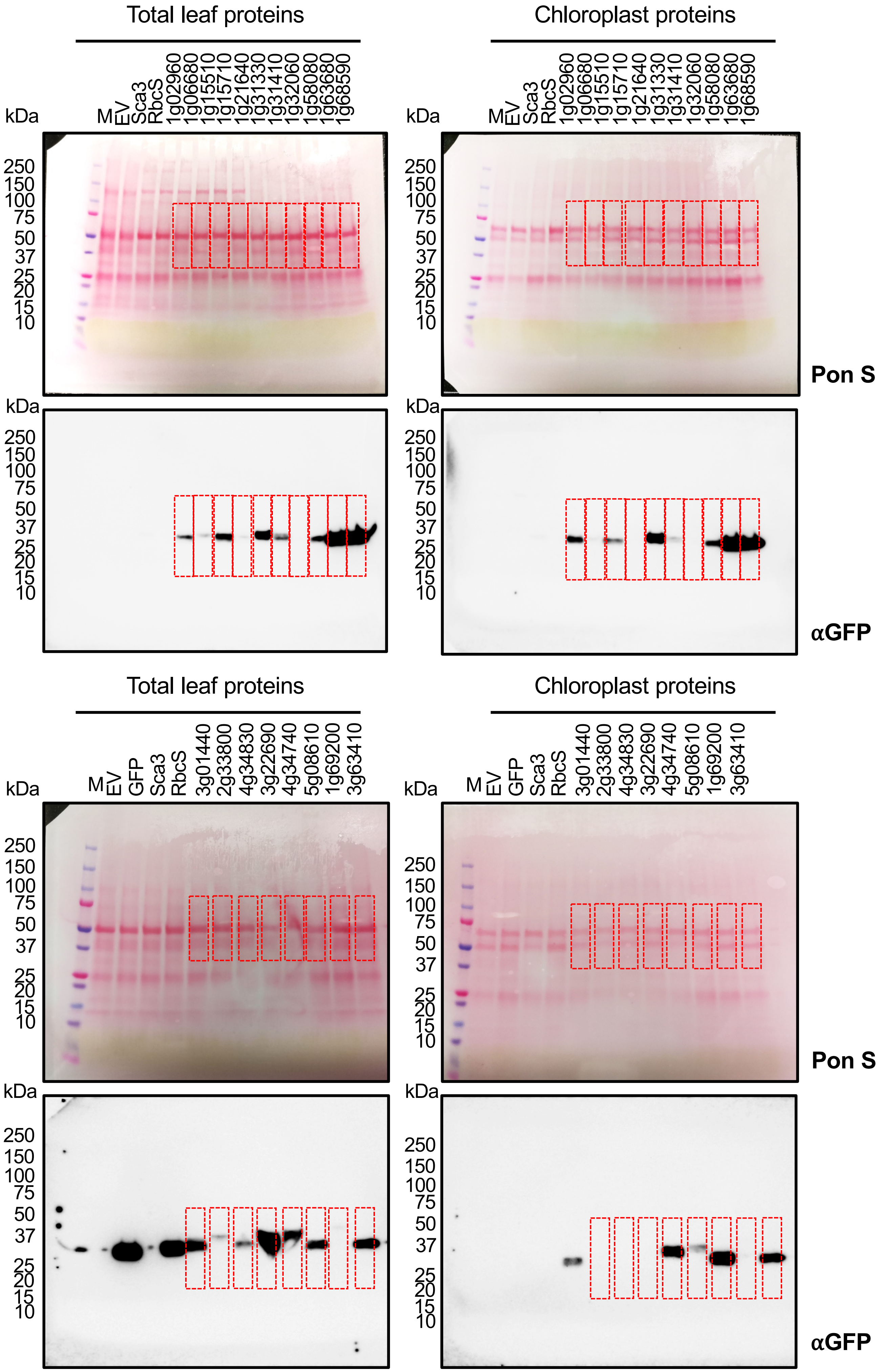

Note ; Red rectangles show cropped images in representative figures.

Original blot images for S2-8 Figs.

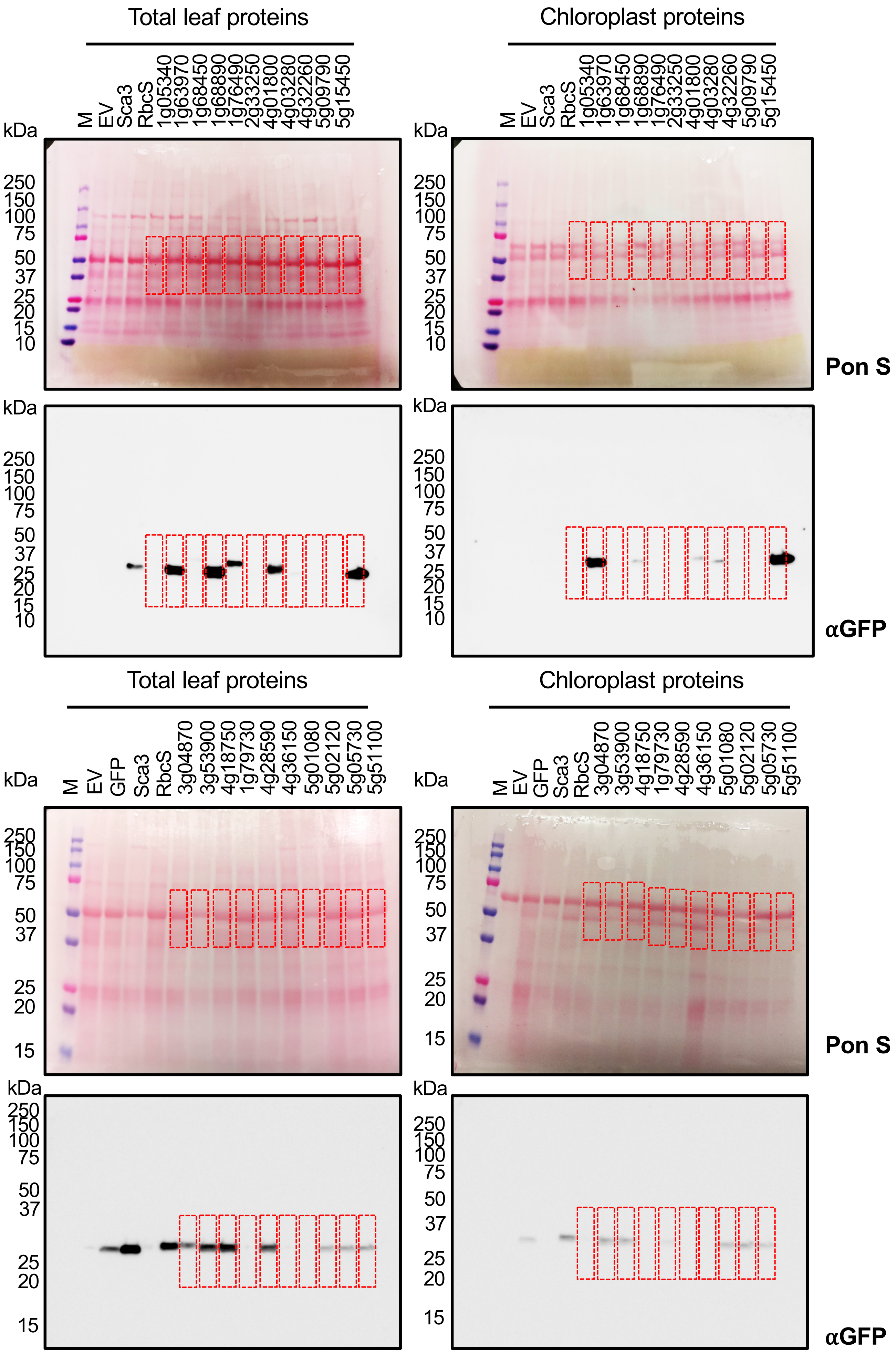

Note ; Red rectangles show cropped images in representative figures.

S2-8 Figs

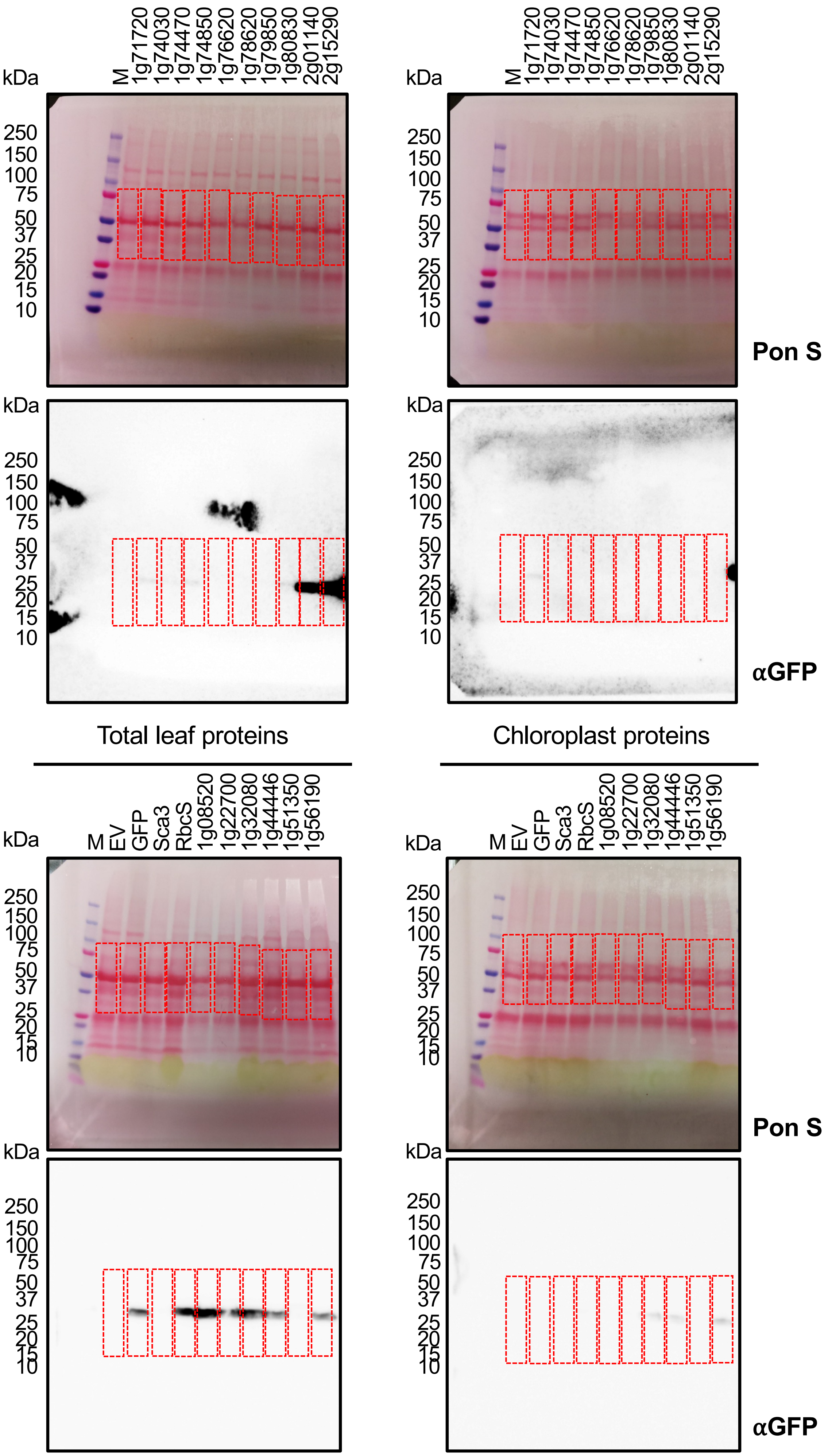

Note ; Red rectangles show cropped images in representative figures.

Original blot images for S2-8 Figs.

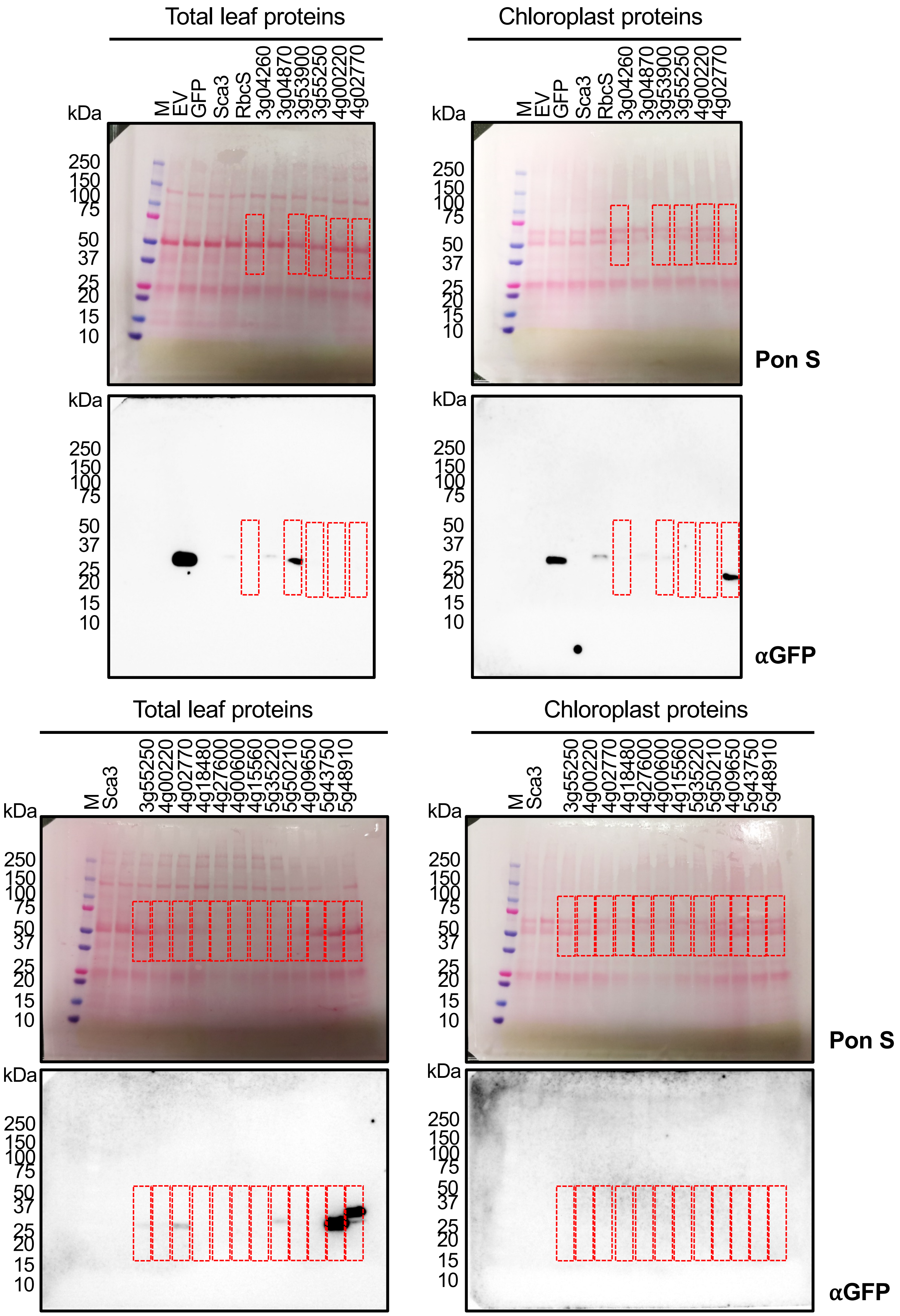

Note ; Red rectangles show cropped images in representative figures.

Immunoblot analysis of cTP-GFPs in total leaf proteins and chloroplast proteins in Fig 2E,F

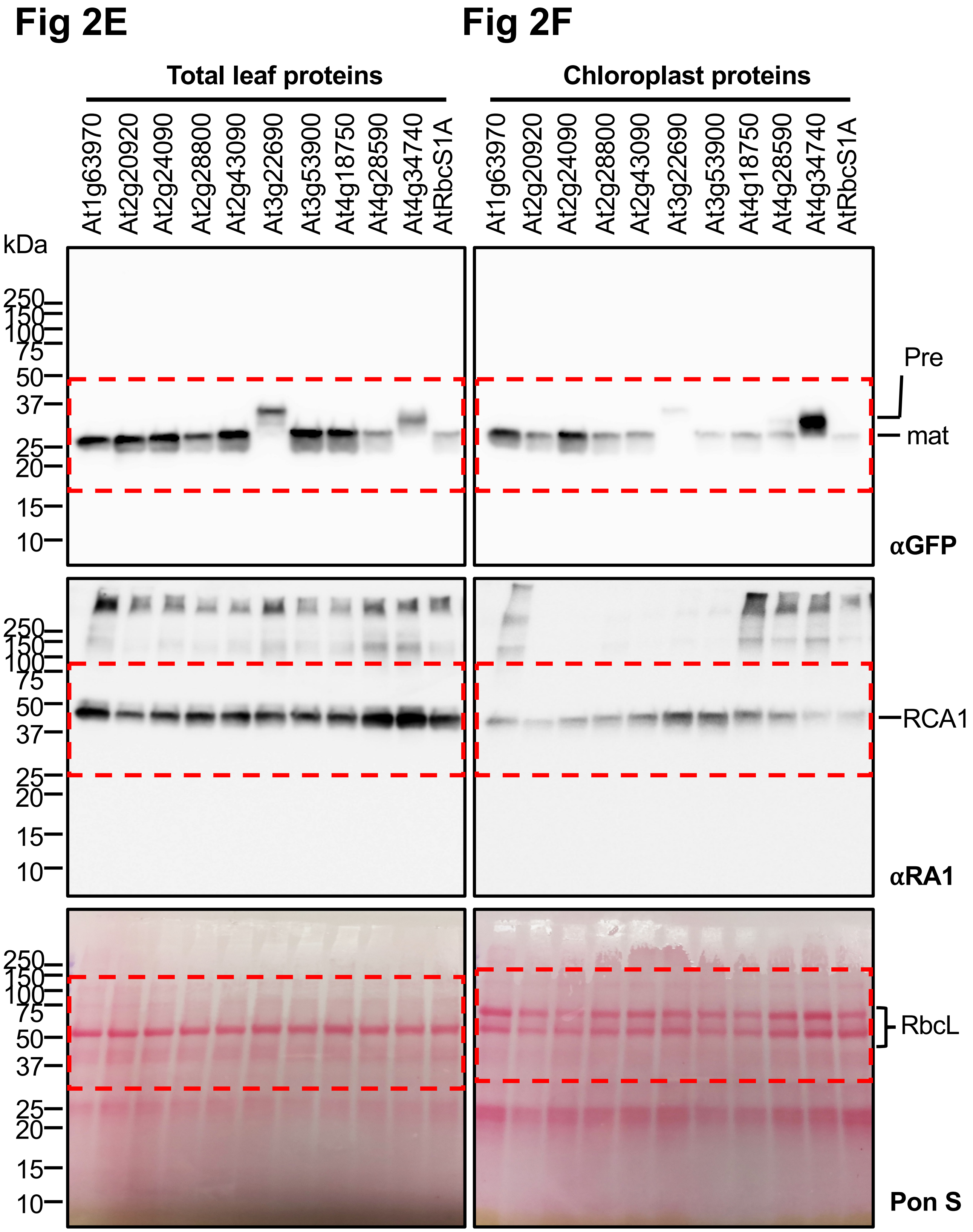

Note ; Red rectangles show cropped images in representative figures.

S12 Fig, Experiment 1

Note ; Red rectangles show cropped images in representative figures.

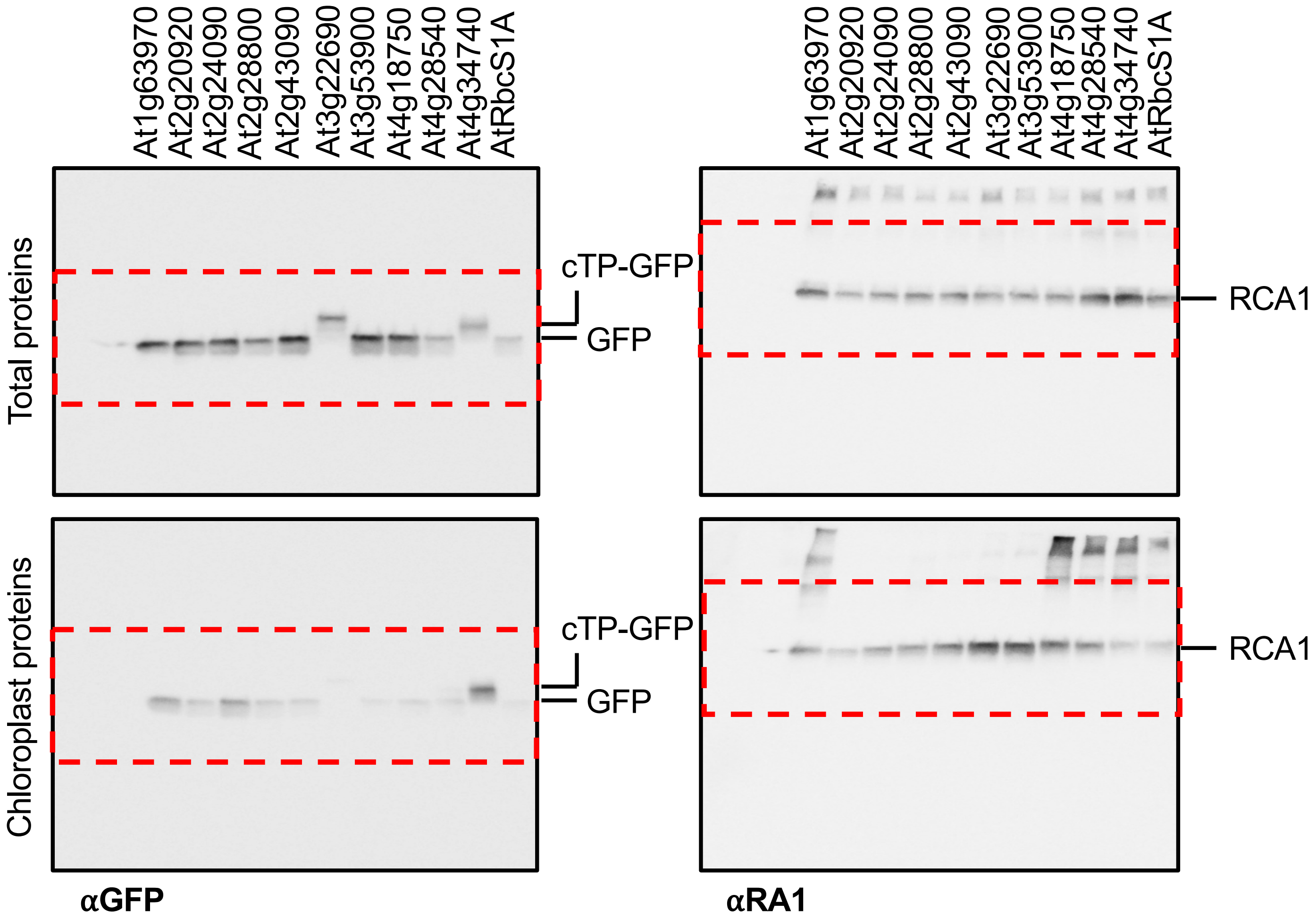

S12 Fig, Experiment 2

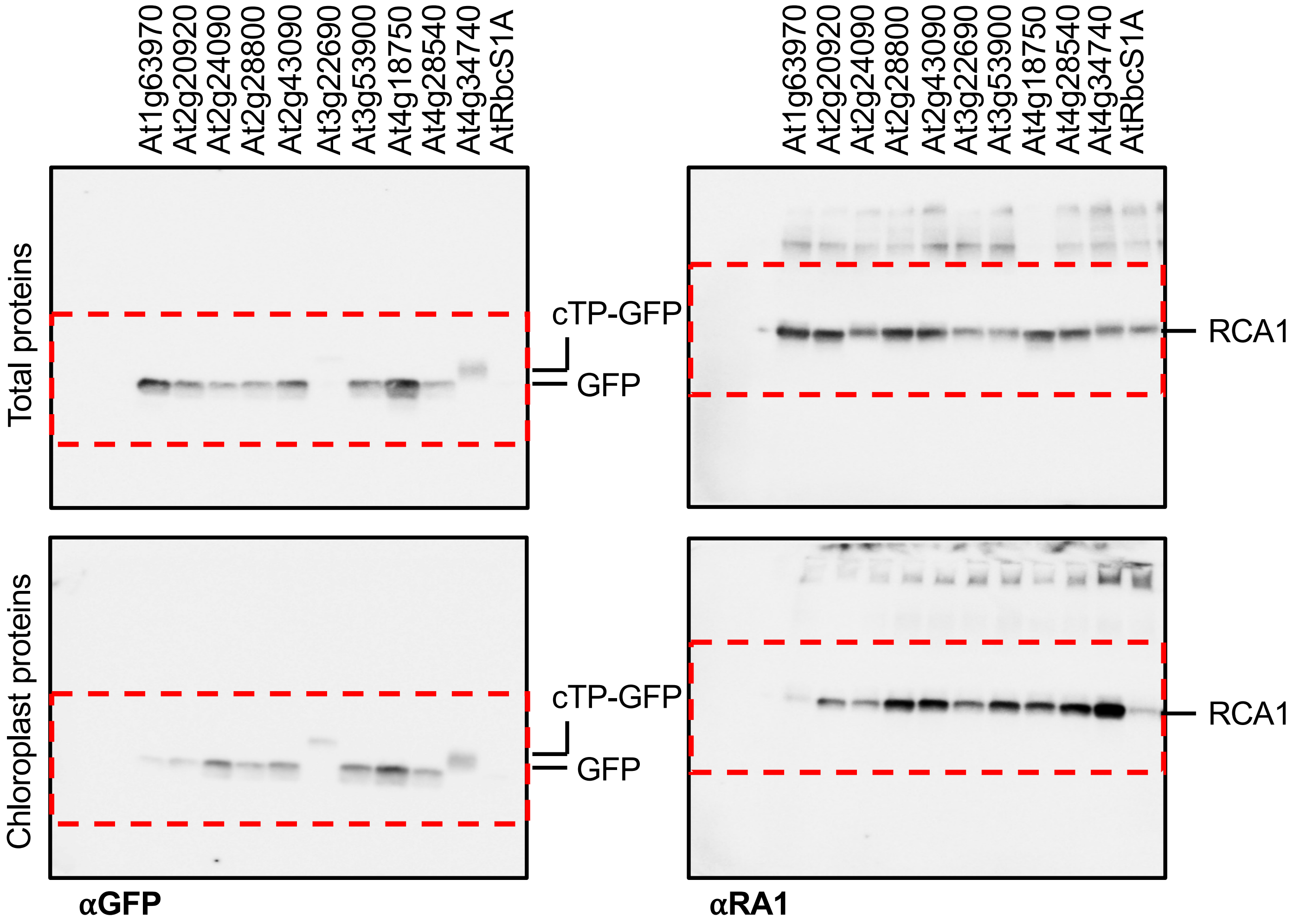

S12 Fig, Experiment 3

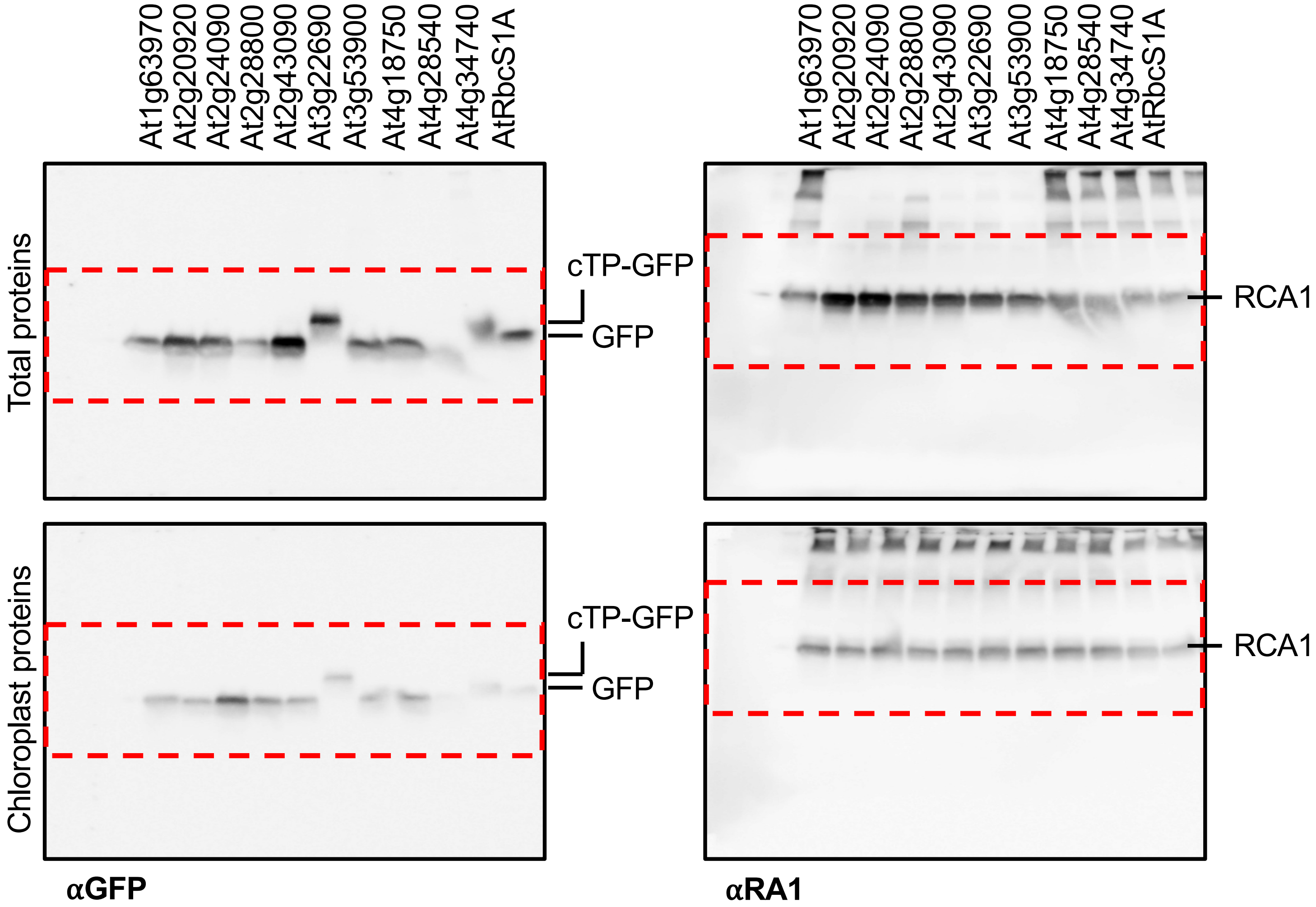

Note ; Red rectangles show cropped images in representative figures.

Analysis of proteins from *in vitro* translation (Fig 3A-B)

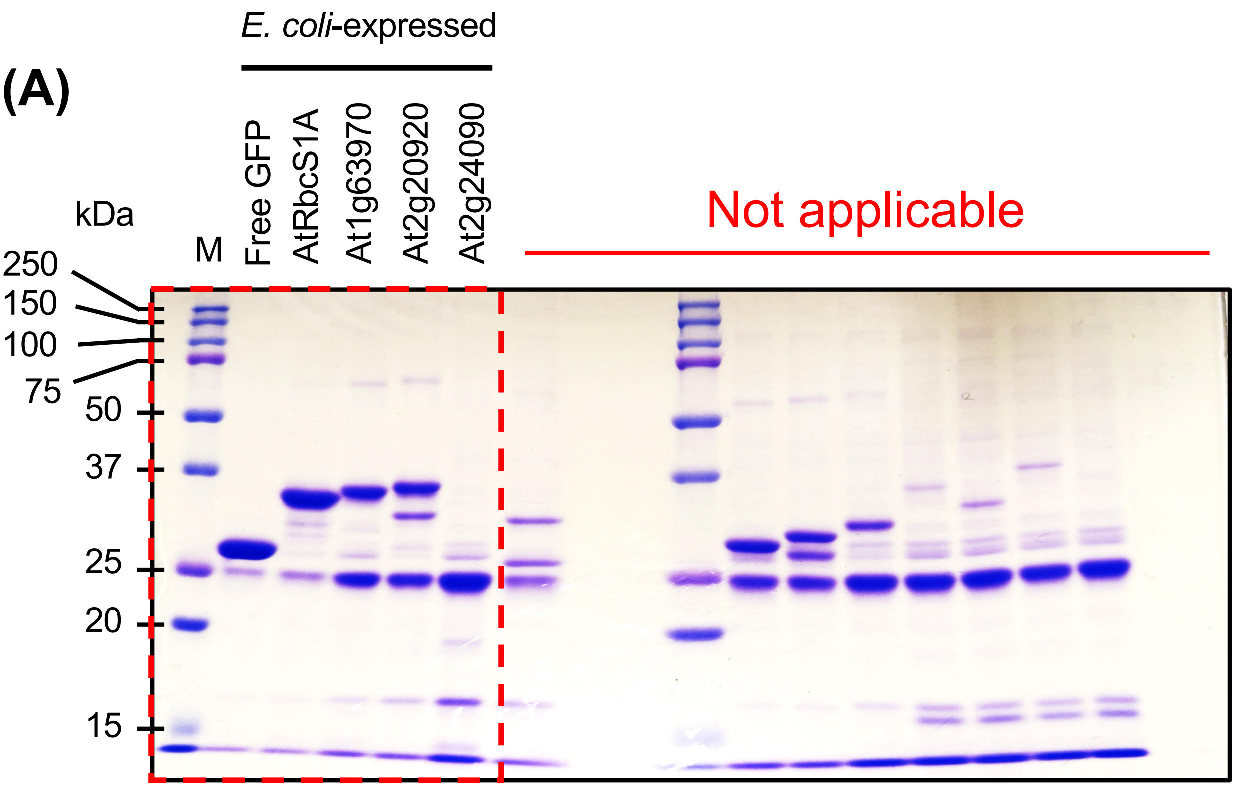

CBB-stained SDS-PAGE

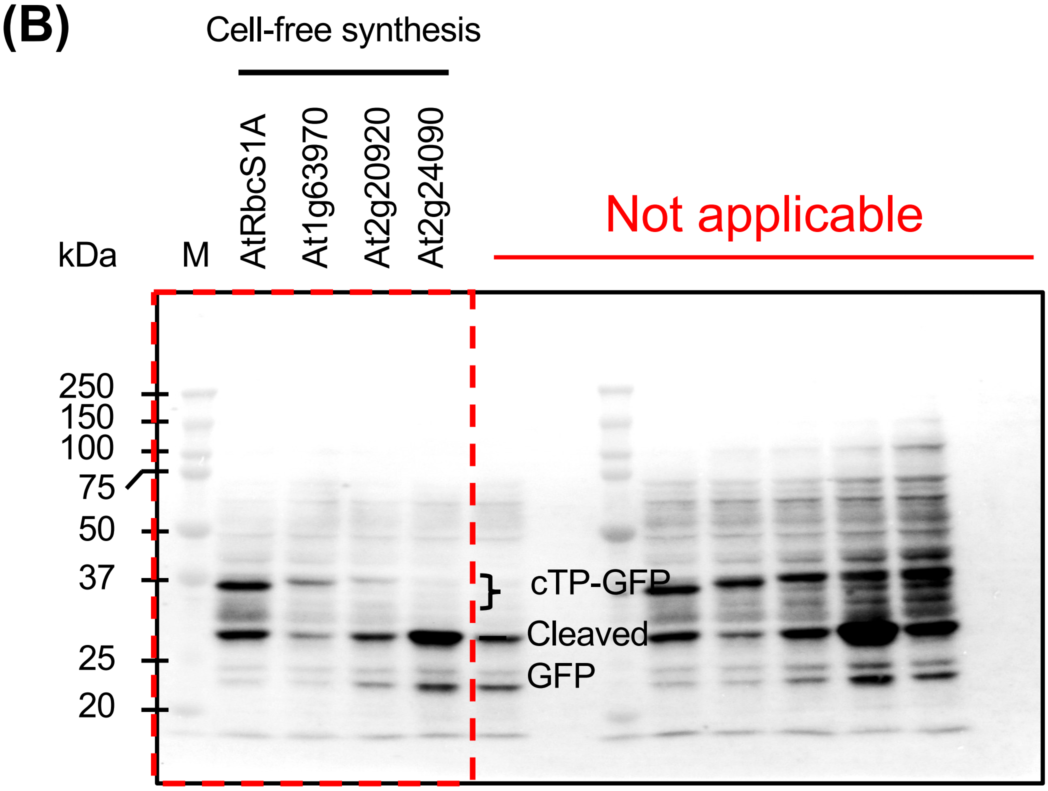

αGFP WB

Note ; Red rectangles show cropped images in representative figures.

Analysis of proteins from *in vitro* translation (Fig 3C-D)

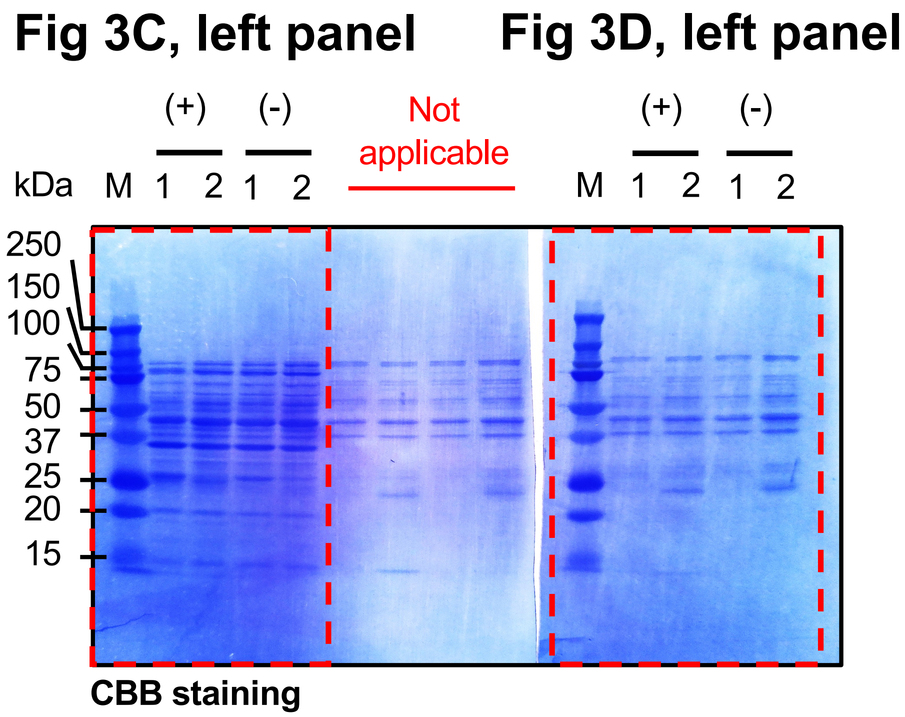

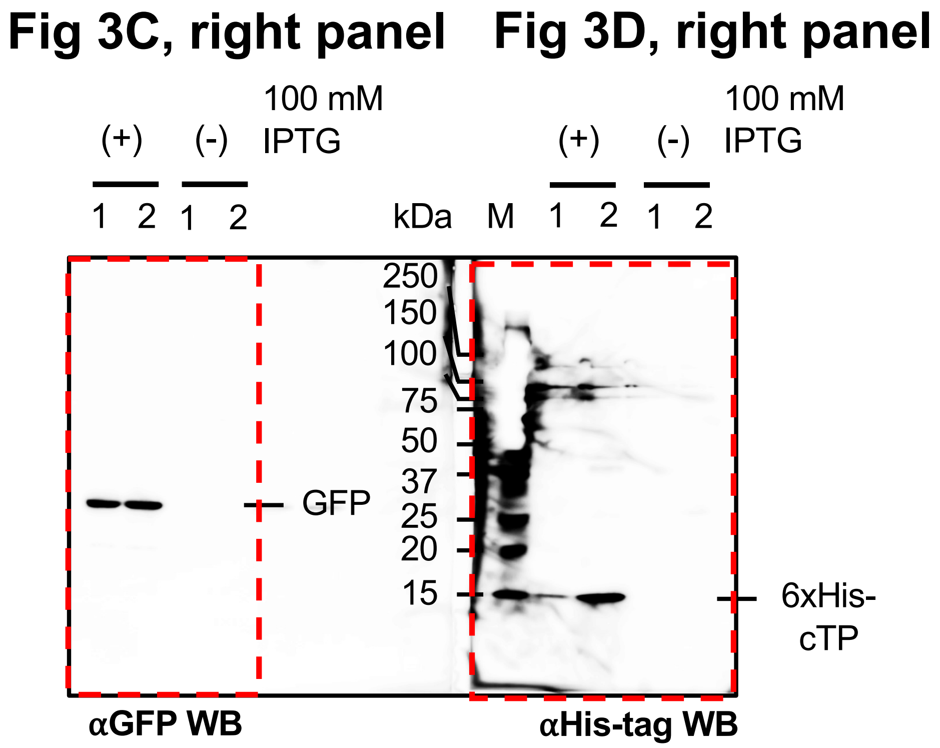

Note ; Red rectangles show cropped images in representative figures.

Fig 3E. *In vitro* import assays of recombinant cTP-GFPs

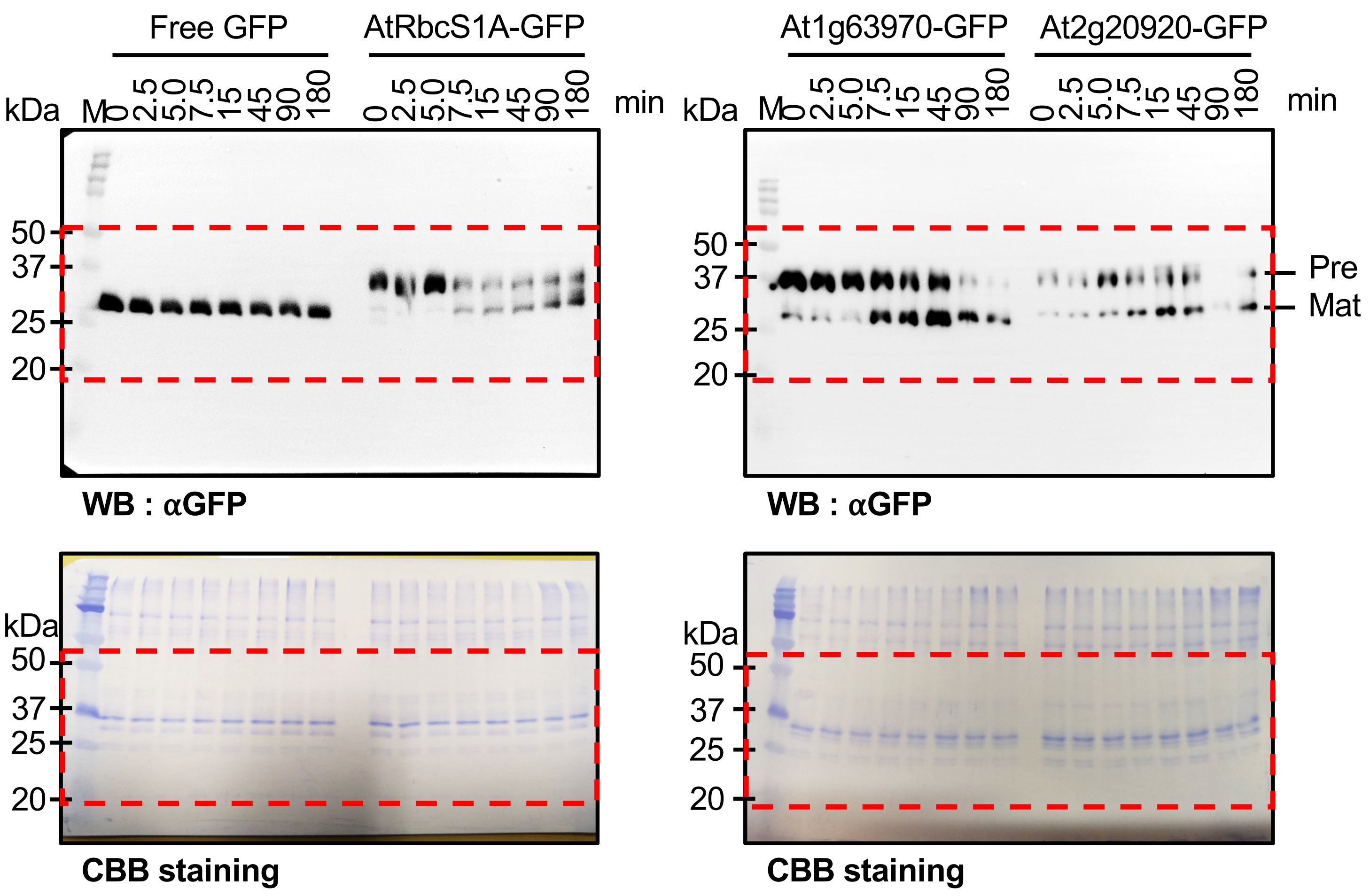

Note ; Red rectangles show cropped images in representative figures.

S13 Fig. *In vitro* import analysis of recombinant cTP-GFP

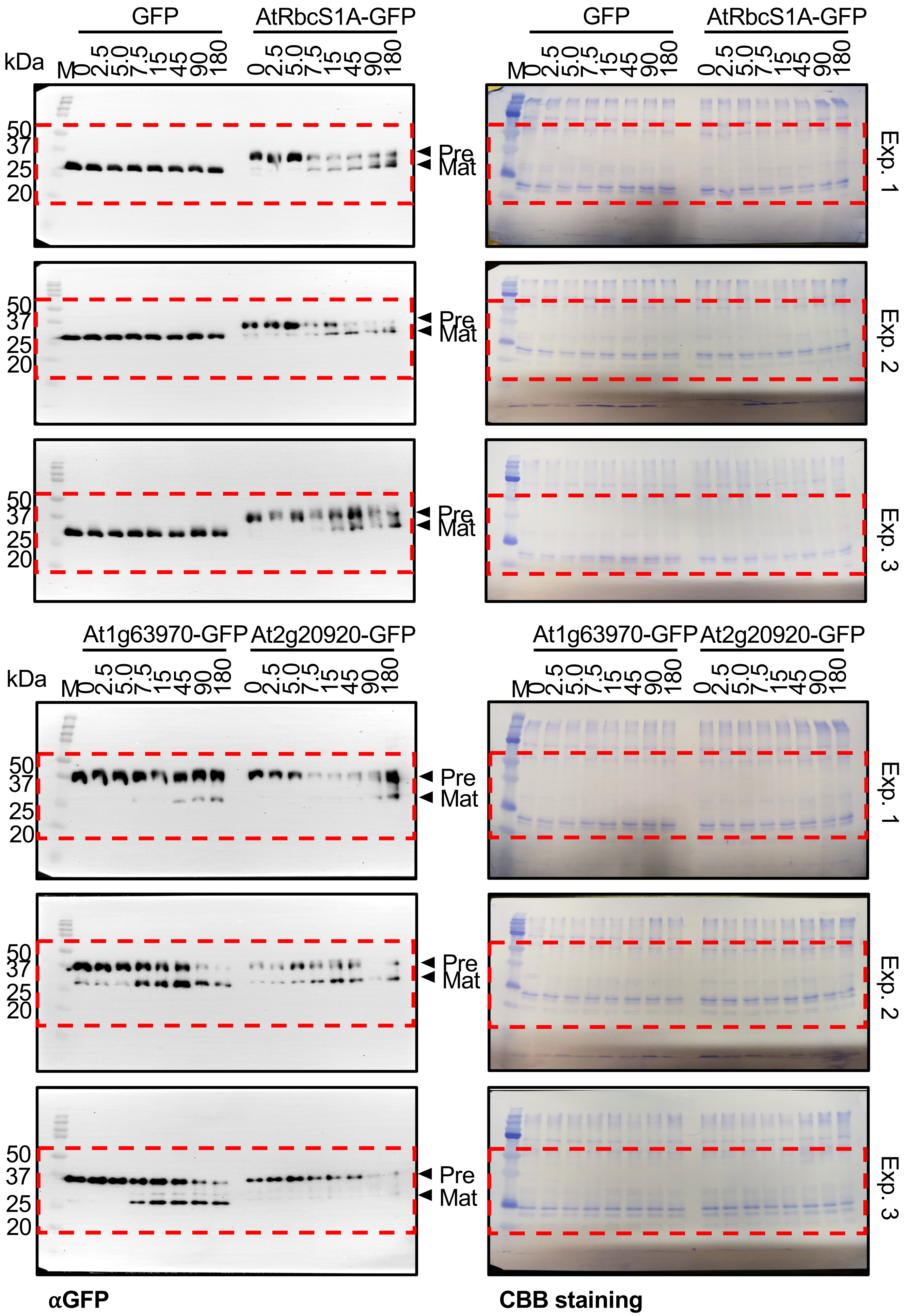

Note ; Red rectangles show cropped images in representative figures.

S13 Fig. *In vitro* import analysis of recombinant cTP-GFP

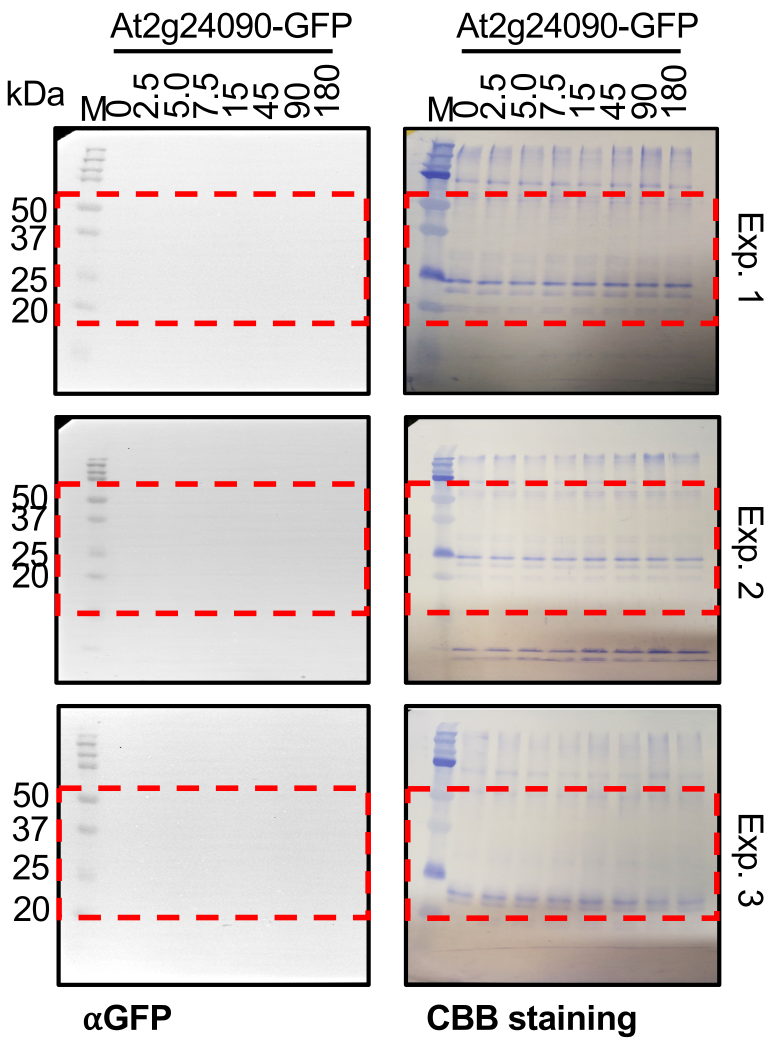

Note ; Red rectangles show cropped images in representative figures.

Fig 4E. Time-course expression analysis of recombinant cTP-GFPs in agroinfiltrated plant leaves

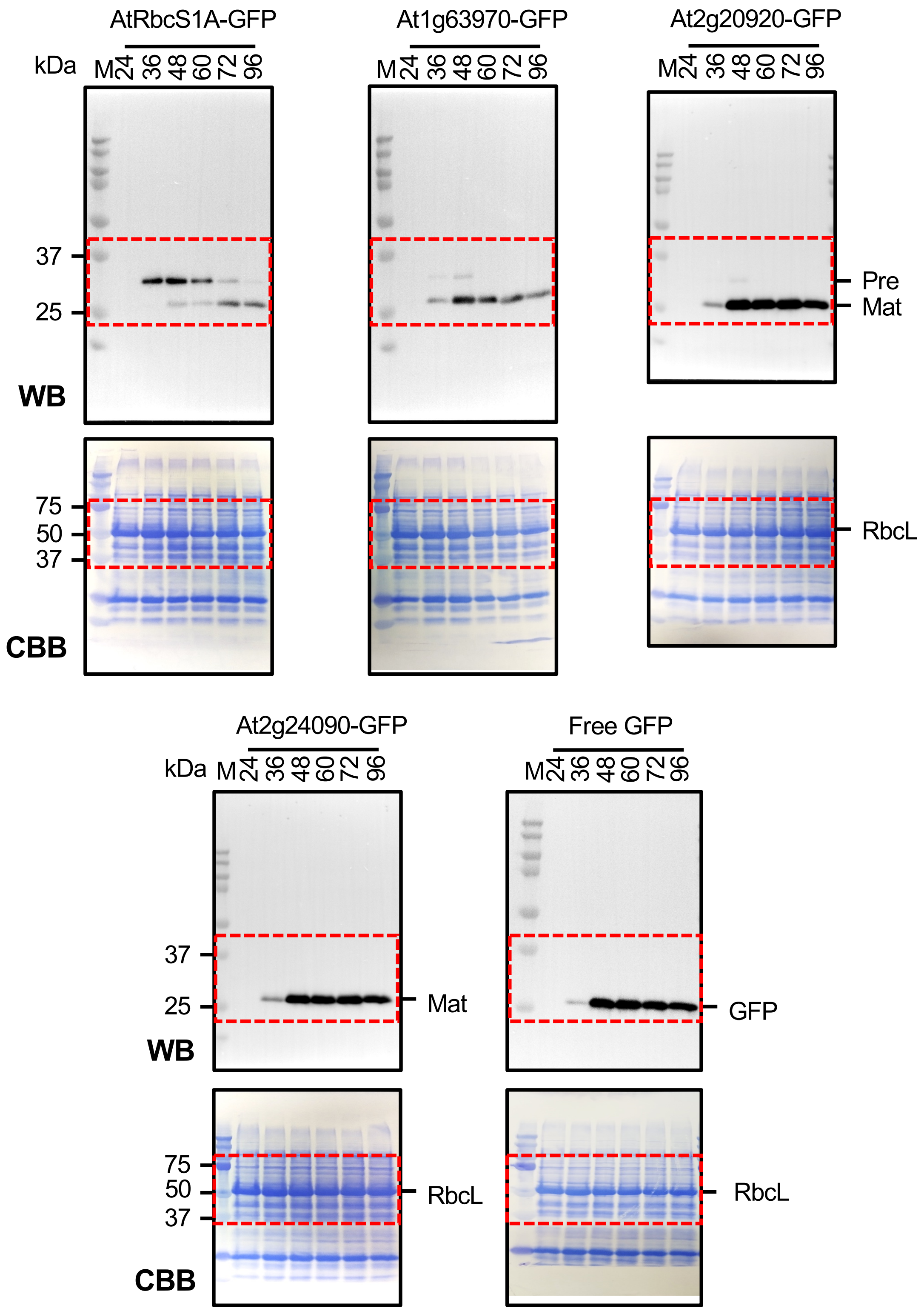

Note ; Red rectangles show cropped images in representative figures.

S15 Fig. *In vivo* transport of recombinant cTP-GFP to chloroplasts

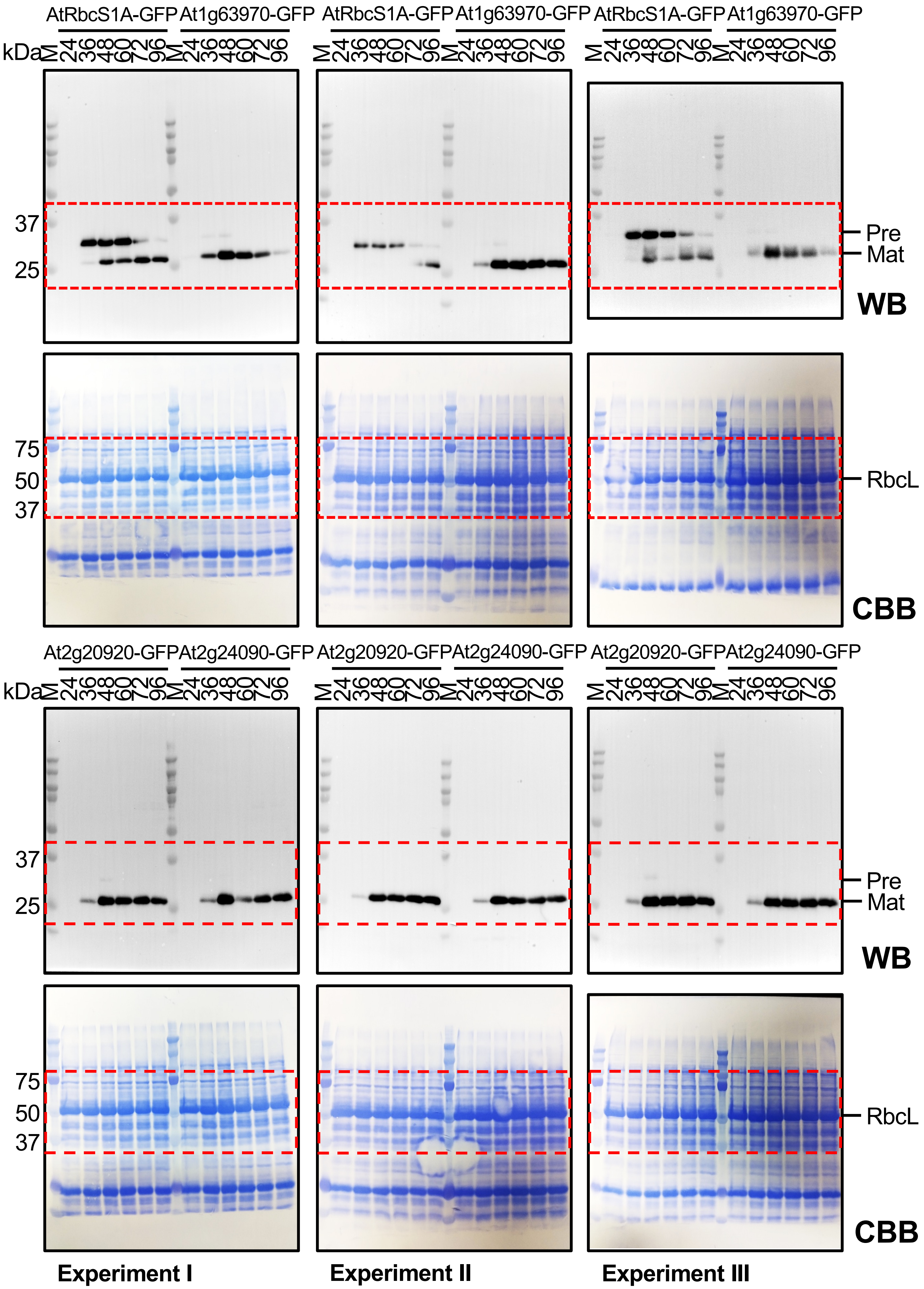

Note ; Red rectangles show cropped images in representative figures.

S15 Fig. *In vivo* transport of recombinant cTP-GFP to chloroplasts

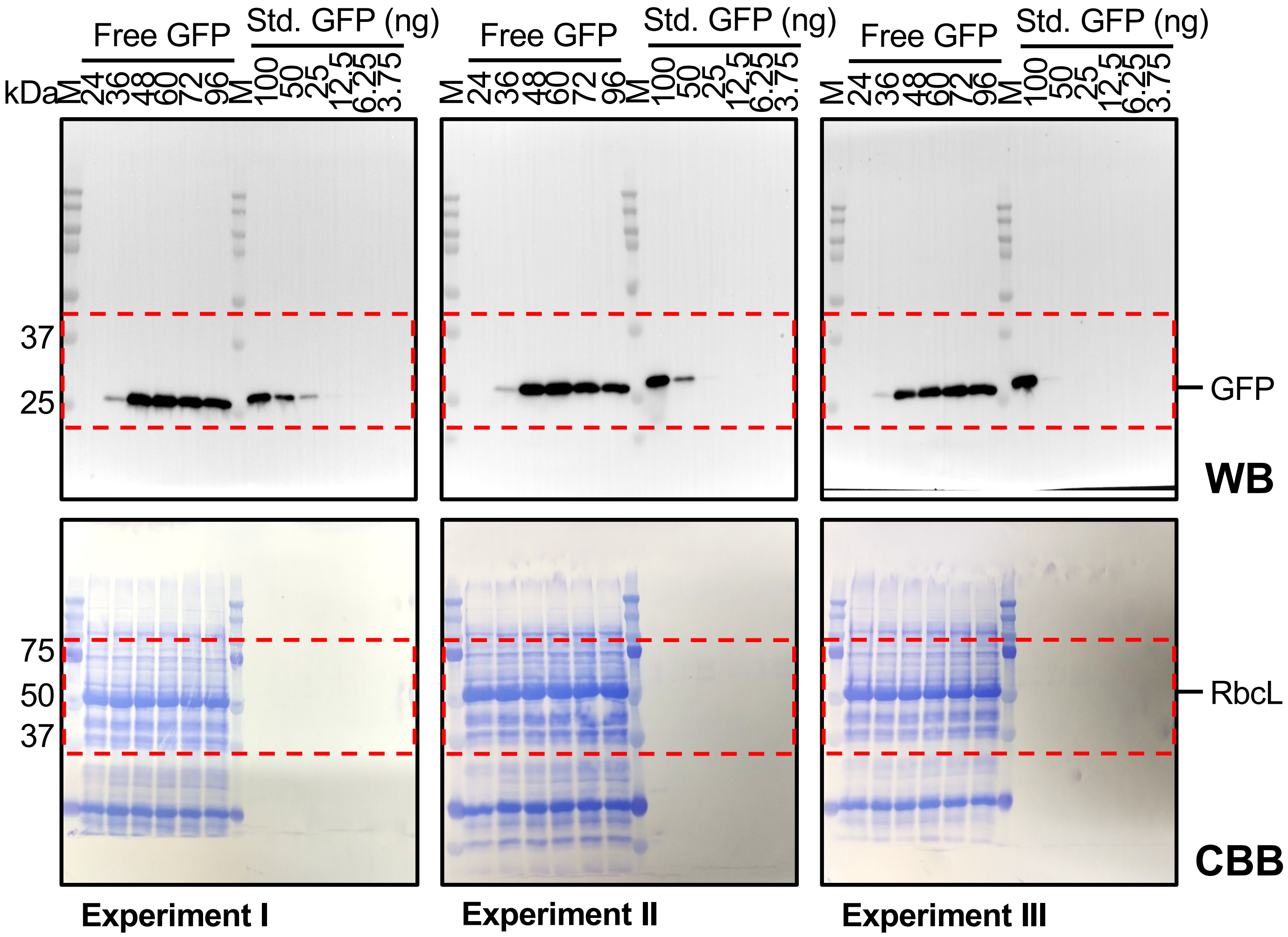

Note ; Red rectangles show cropped images in representative figures.

Fig 5B. *In vitro* import activity of truncated At2g24090-GFPs to isolated chloroplasts

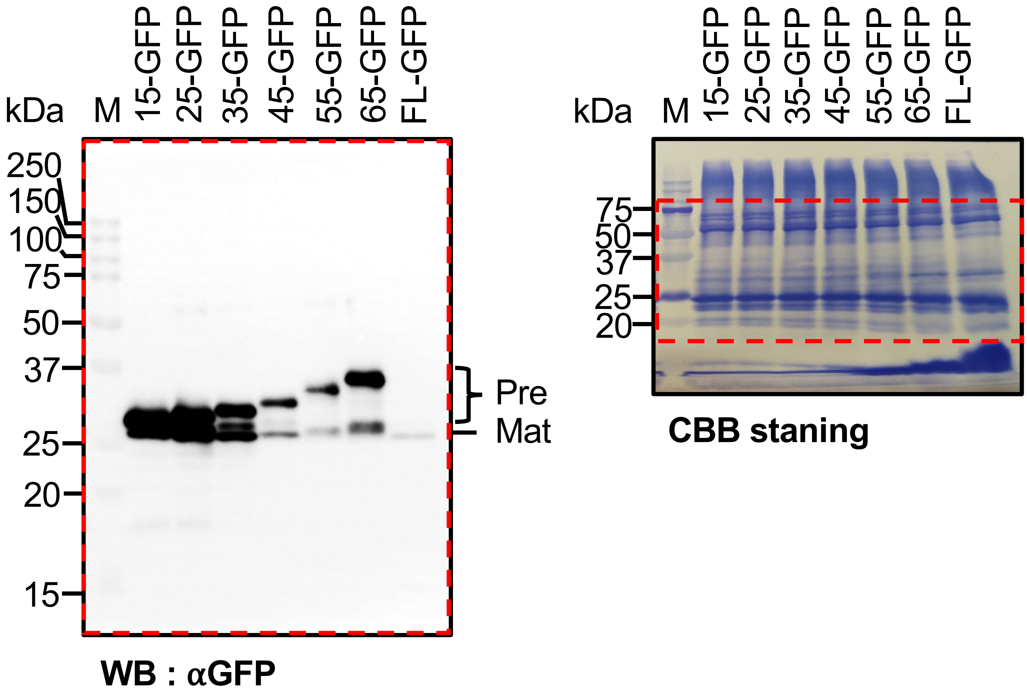

Note ; Red rectangles show cropped images in representative figures.

Fig 5D. Time-course import assays of truncated At2g24090-GFPs to isolated tobacco chloroplasts

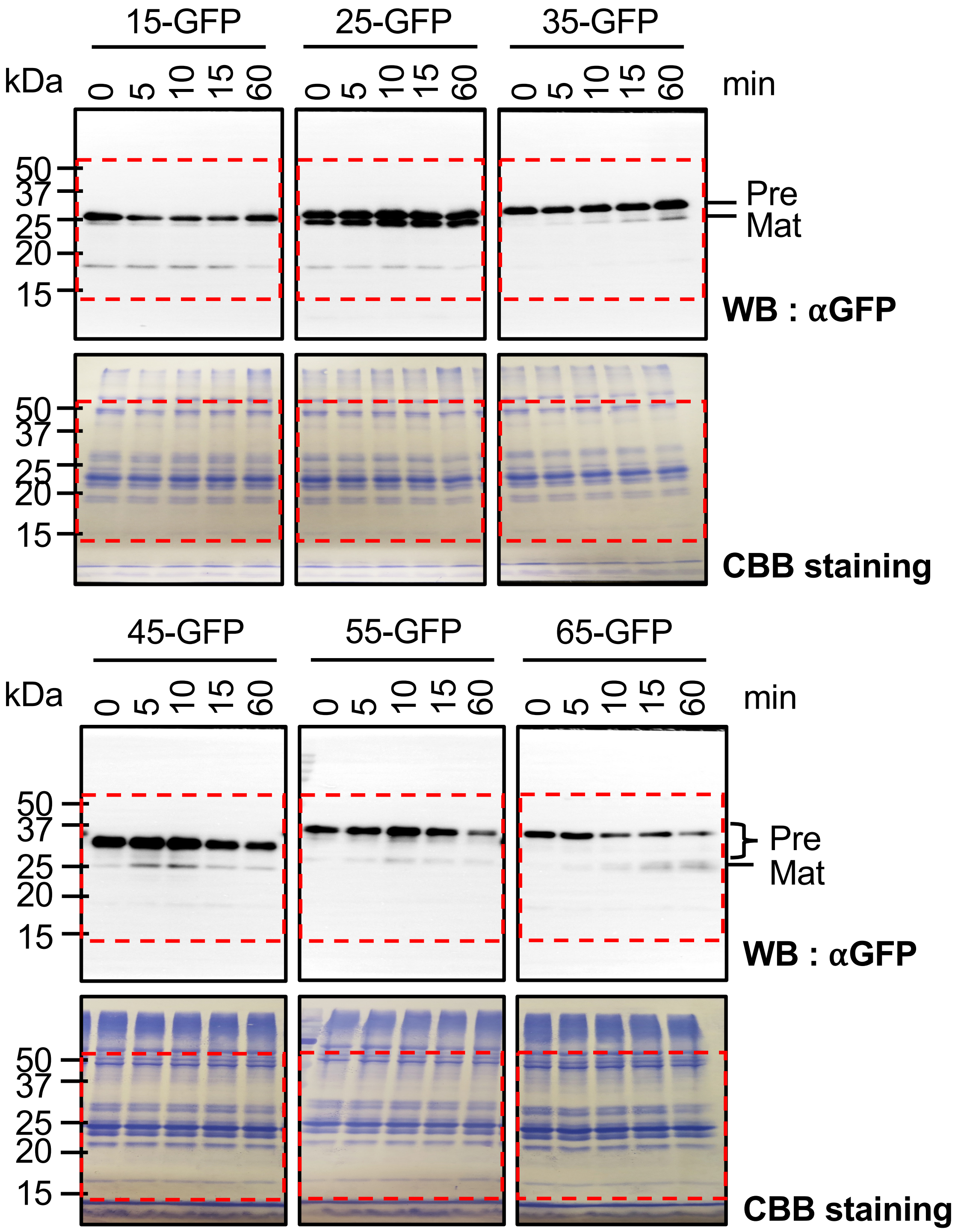

Note ; Red rectangles show cropped images in representative figures.

Fig 5H. Accumulation of truncated At2g24090-GFPs in total leaf proteins and chloroplasts proteins

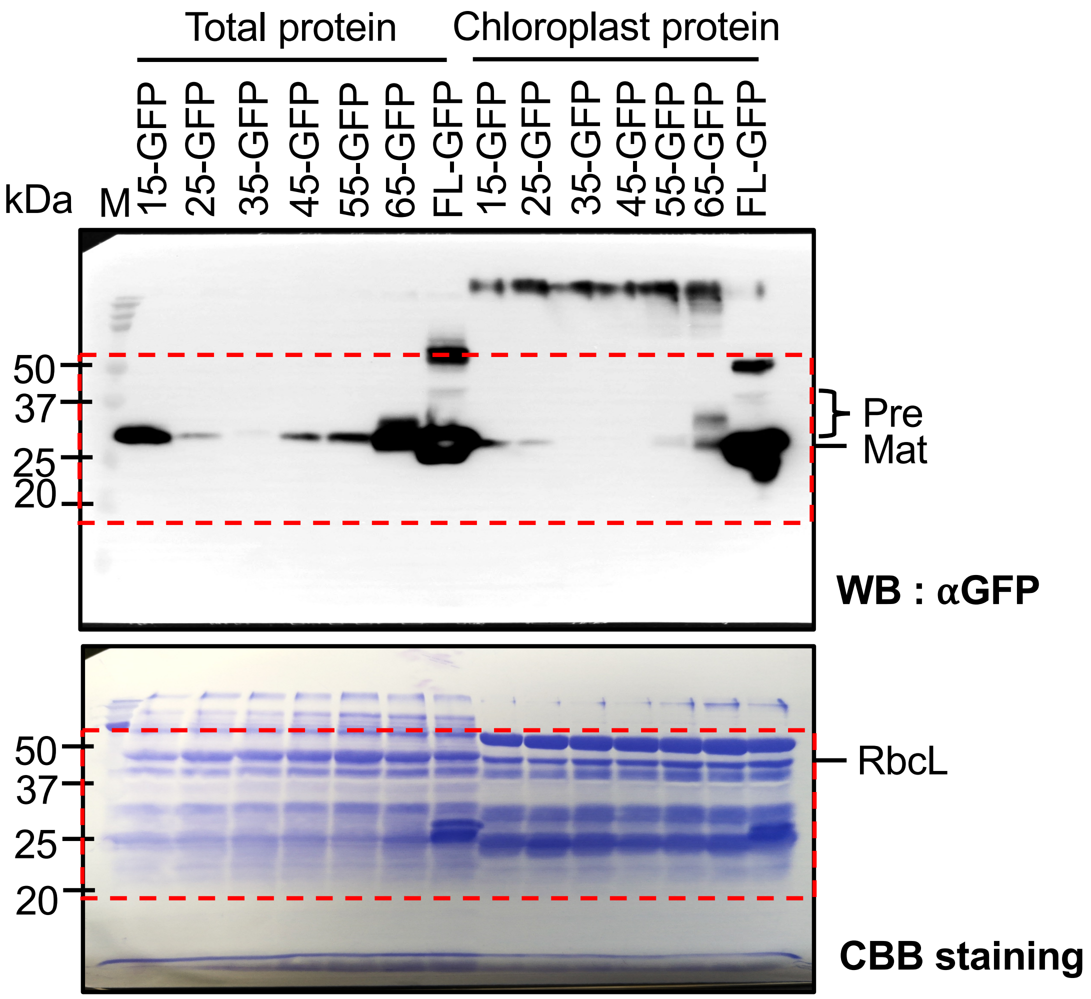

Note ; Red rectangles show cropped images in representative figures.

S17B Fig. Immunoblot analysis of truncated At2g24090-GFPs in total leaf proteins and chloroplasts proteins from agroinfiltrated tobacco leaves

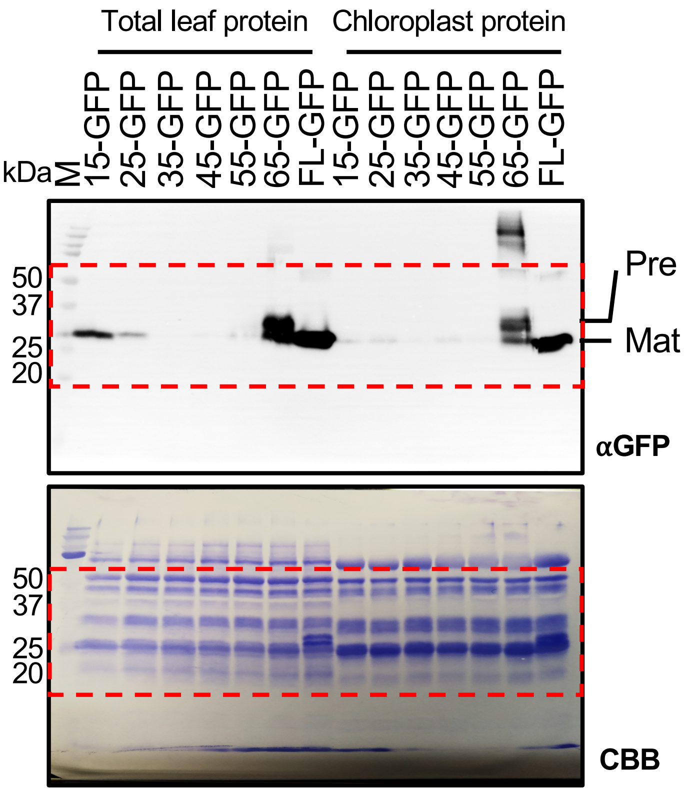

Exp. 1

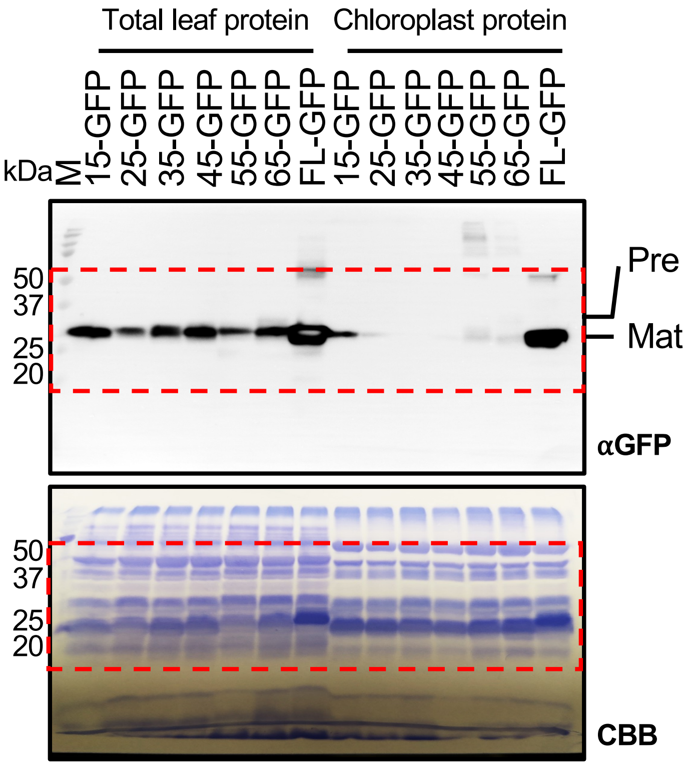

Exp. 2

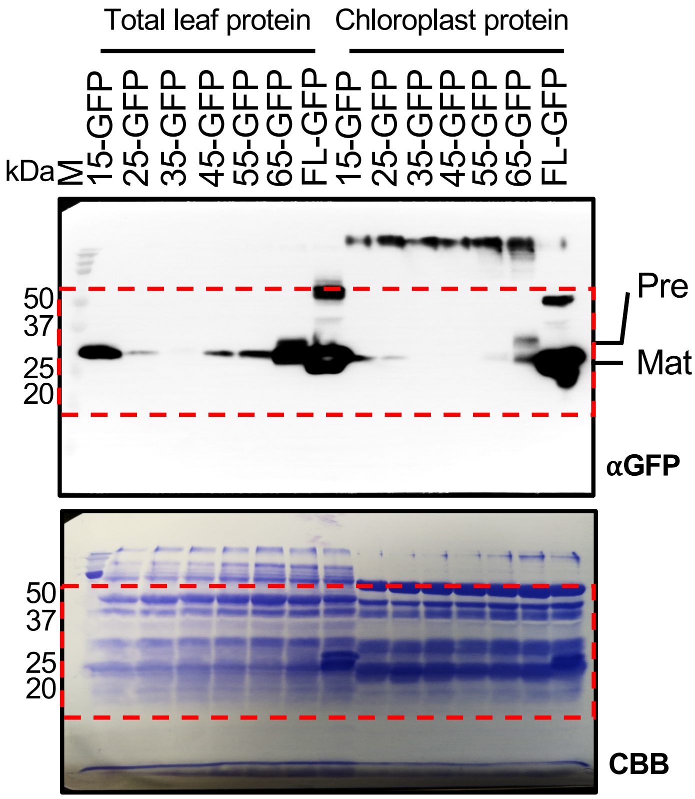

Exp. 3

Note ; Red rectangles show cropped images in representative figures.

S18D Fig. Western blotting of different cTP-GFPs in total leaf proteins and chloroplast proteins isolated from agroinfiltrated tobacco leaves

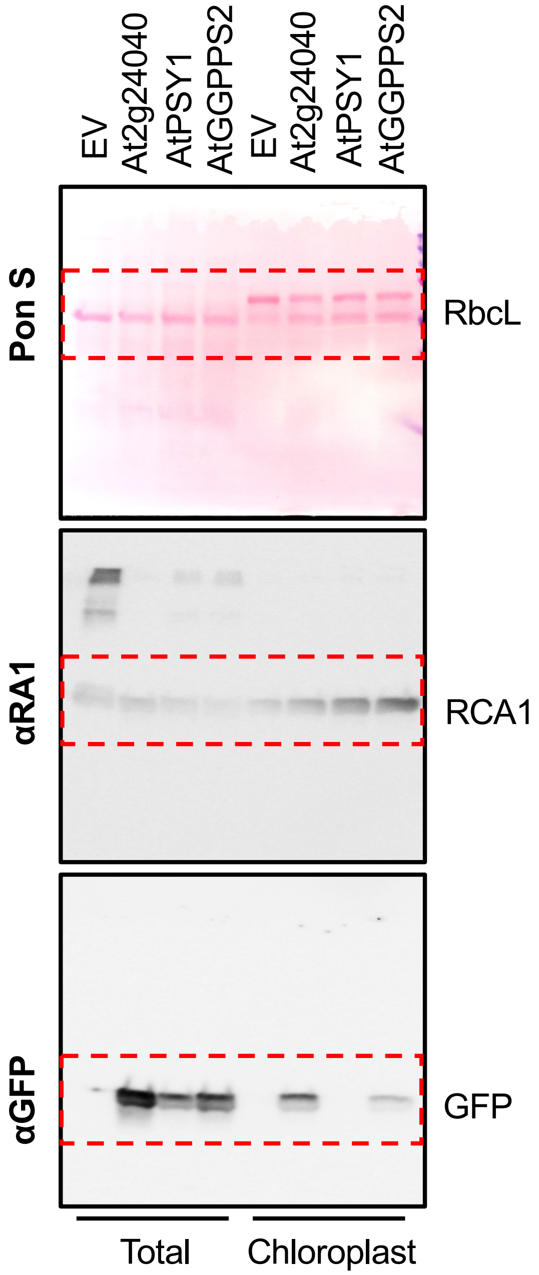

Note ; Red rectangles show cropped images in representative figures.

S19B Fig. Expression of recombinant cTP-GGPPS2-FLAG proteins in tobacco leaf proteins (T) and chloroplast proteins (C)

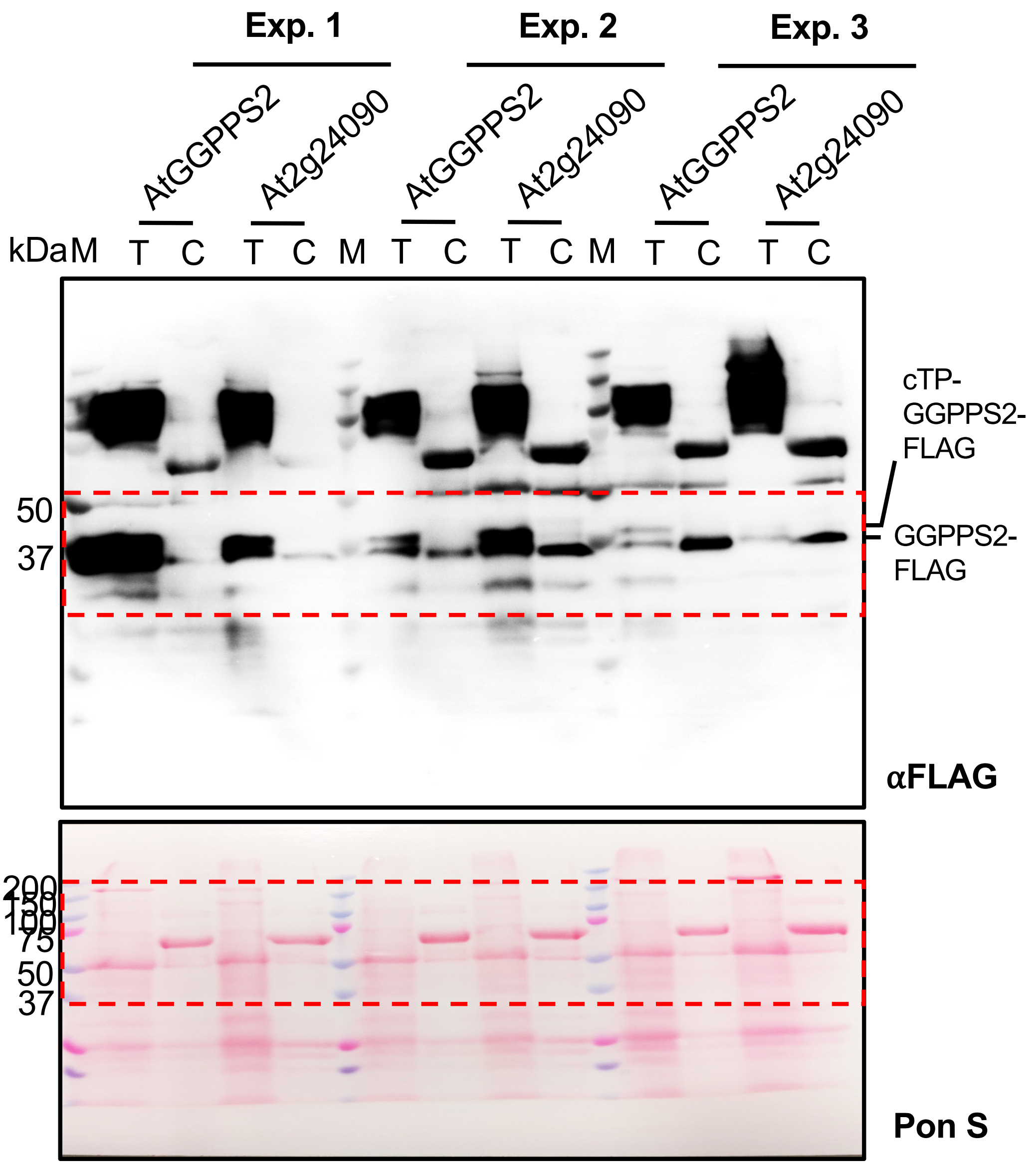

Note ; Red rectangles show cropped images in representative figures.

Fig 7F. Immunoblot analysis of different cTP-GFPs in total leaf proteins and chloroplast proteins of agroinfiltrated tobacco leaves

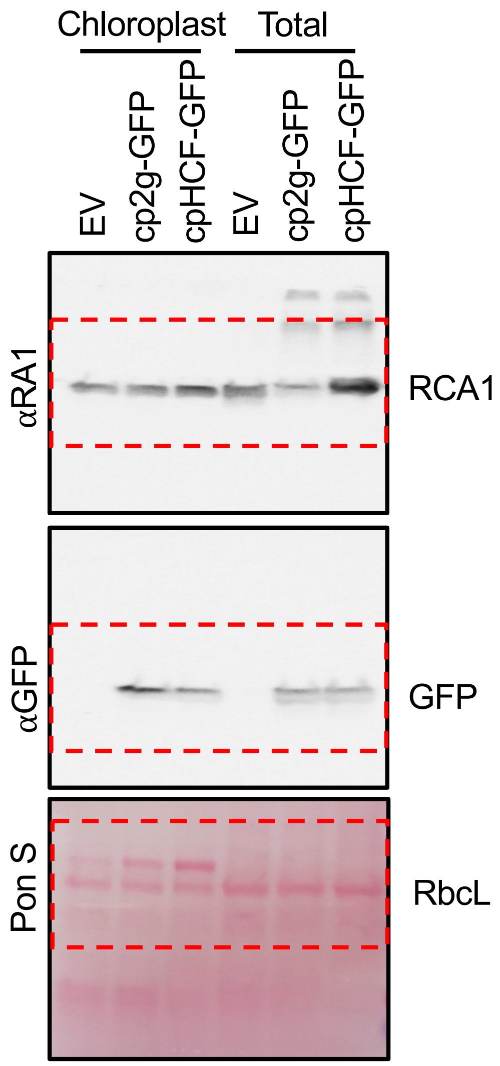

Note ; Red rectangles show cropped images in representative figures.
