# Supplementary material for: Identification of a highly efficient chloroplast-targeting peptide for plastid engineering
Source: PLoS Biol. 2024 Sep 19;22(9):e3002785. doi: 10.1371/journal.pbio.3002785 (PMC11444414; doi:10.1371/journal.pbio.3002785)
Supplement: Attachment — Submitted filename: Response to Reviewers Comments.docx [file pbio.3002785.s047.docx]

**Response to Reviewers’ Comments**

**Reviewer 1**
This manuscript describes the screening of a large number of chloroplast transit peptides (cTPs) for their effectiveness at driving the accumulation of GFP in chloroplasts. One particularly effective cTP (from the chloroplast ribosomal protein L35) was discovered and then further analysed via deletion analysis (unsurprisingly, the full-length cTP was the most effective). Importantly, the authors demonstrated that the effectiveness of the L35 cTP was not limited to GFP fusions by showing that other cargo proteins also accumulated to higher levels within chloroplasts. The findings in this manuscript will be of great practical interest to many plant biologists and biotechnologists, as targeting proteins to chloroplasts is a common requirement of many projects.

**Response**: Thank you very much for your valuable time and effort in helping us review our manuscript and providing critical recommendations and suggestions to improve our work. After revising our manuscript according to the reviewers’ comments, we hope it will appeal to a broader audience who share common interests in plant biology, chloroplast biotechnology, and other scientists working in different fields. In the revision process, we conducted additional experiments to address the critical concerns raised by the reviewers and modified the current version of the manuscript accordingly. Please find our responses to your point-by-point comments below.

>> **Major point**
Much of the text, as written, appears to assume that the higher level of GFP fluorescence in chloroplasts observed when the L35 cTP is used is due to more efficient translocation of the fusion protein into chloroplasts. However, the fluorescence in this end-point assay depends quantitatively on many other factors that may be affected by the sequence of the cTP. These factors include, for example, mRNA stability, translational efficiency, interaction with cytosolic chaperones, protein folding (or unfolding), protein stability, and the rate of GFP fluorophore formation. The only direct measure of the rate of translocation into chloroplasts (Fig 3) failed to provide any evidence for improved import mediated by the L35 cTP. So I think the authors should be much more careful about the interpretation of their results, and rephrase their text to remove any implicit or explicit assumptions about the mechanism by which the L35 cTP acts to boost the accumulation of cargo proteins in
chloroplasts. It could well be that more GFP is found in chloroplasts when expressed as an L35 cTP fusion because more preprotein is synthesized in the first place, and not because it is more efficiently translocated. I emphasize that this is a pervasive issue in the text — there are dozens of places where it is written that the L35 cTP improves translocation, despite the lack of any evidence that it does so.

**Response**: The authors are grateful for the reviewer’s suggestion to provide experimental evidence supporting our conclusion regarding the higher activity of At2g24090 cTP in transporting its conjugated proteins to chloroplasts. Additionally, we aim to clarify the interpretation of our critical results, which support the assumption that the selected cTPs enhance “translocation efficiency,” leading to increased accumulation of GFP and other proteins within chloroplasts.

In response to your comment, we conducted a time-course expression analysis of the selected recombinant cTP-GFPs in tobacco leaves, as detailed in our revised manuscript. For this experiment, plant expression vectors containing different cTP-GFP expression cassettes, previously chosen for *in vitro* import assays, including At1g63970-, At2g20920-, At2g24090-GFP, AtRbcS1A-GFP (as a reference cTP), and GFP without a transit peptide fusion (free GFP), were transiently expressed in tobacco leaves using agroinfiltration.

We analyzed the differential expression of transcripts and recombinant cTP-GFPs using qRT-PCR and fluorometry-based measurements of the fluorescent proteins overexpressed in plant samples collected at 24, 36, 48, 60, 72, and 96 hours after infiltration (HAI), respectively. Our expression analyses revealed that although transcript expression levels of different cTP-GFP genes varied from 24 to 60 HAI (**Fig. 4A**), there was no statistically significant difference in the accumulation of recombinant cTP-GFPs in total leaf proteins (**Fig. 4B**). These findings suggest that transient expressions of different cTP-GFP-coding sequences did not affect the accumulation levels and the formation of GFP fluorephores of the corresponding recombinant cTP-GFPs in plant cells.

**Figure 4. *In vivo* translocation of cTP-GFPs**. (**A**) Transcript expression of *cTP-GFP* genes in agroinfiltrated tobacco leaves at different time points post-agroinfiltration analyzed by qRT-PCR using *GFP*-specific primers. The expression levels of *cTP-GFP* transcripts were compared to that of pBI121-transformed leaves (EV) at 24 hours post-infiltration. Relative transcript expression was determined from three biologically independent agroinfiltrated leaves (*n* = 3). Error bars represent standard deviation (SD). (**B**) Abundance of various cTP-GFP fluorescent proteins in total leaf proteins. GFP fluorescence in total proteins extracted from agroinfiltrated tobacco leaves at different time points was measured, and the amounts of fluorescent protein in three experimentally independent samples (*n* = 3) were calculated from the linear regression equations generated from the fluorescent intensities of standard GFP. Error bars represent SD.

Following this, we investigated the translocation of different recombinant cTP-GFPs to chloroplasts in transformed plant cells. The temporal localization and chloroplast targeting of these recombinant cTP-GFPs within plant cells were detected using confocal microscopy, followed by image analysis. Our fluorescence microscopic analyses revealed distinct localization patterns of recombinant cTP-GFPs in tobacco leaf cells (**Fig. 4C,D**). Interestingly, AtRbcS1A-GFP initially exhibited partial distribution in the cytosol at 24–60 HAI before gradually localizing to the chloroplasts (**Fig. 4C**). Conversely, At1g63970-, At2g20920-, and At2g24090-GFP demonstrated significantly stronger chloroplast-specific GFP fluorescent signals at all time points compared to the control AtRbcS1A-GFP (**Fig. 4C,D**, Fig S14). Our fluorescence microscopy results suggest that the three selected cTPs from the screened cTP library possess superior chloroplast import abilities compared to the AtRbcS1A cTP.

**Figure 4. *In vivo* translocation of cTP-GFPs**. (**C**) Localization of various recombinant cTP-GFPs in transfected tobacco leaf cells at different time points post-agroinfiltration. Scale bars: 20 µm. (**D**) Ratio of GFP fluorescence in chloroplasts to total GFP fluorescence in a CLSM image. CLSM images were captured from transformed plant leaves at different time points. Fluorescent intensities of recombinant cTP-GFPs within chloroplasts and in an ROI of a CSLM image were measured using Fiji ImageJ. Error bars represent SD of sixteen CLSM images (*n* = 16) collected from three independent experiments.

The *in vivo* chloroplast import activities of various cTP-GFPs were further assessed through immunoblotting of total leaf proteins collected at different time points post agroinfiltration. Our immunoblot results, along with GFP band quantification, revealed varying abundances of cTP-GFP precursor proteins and their matured (cleaved) variants in total leaf proteins from 24 to 96 HAI (**Fig. 4E** to **4G**). Notably, AtRbcS1A-GFP exhibited a progressive decrease in its precursor proteins, accompanied by an increase in the accumulation of mature proteins in the samples (**Fig. 4E** to **4G**). Surprisingly, At1g63970- and At2g20920-GFP also displayed partial abundance of their precursor molecules up to 48 HAI (**Fig. 4E**). Additionally, a decrease in matured At1g63970-GFP in total leaf proteins was observed over later post-infiltration periods (**Fig. 4E** and **4G**). In contrast, At2g24090-GFP exhibited undetectable levels of its precursor in total leaf proteins, alongside gradually increasing levels of mature form from 24 to 48 hours and steady levels from 48 to 96 HAI (**Fig. 4E** to **4H**). Furthermore, these recombinant cTP-GFPs (AtRbcS1A, At1g63970, At2g20920, and At2g24090) displayed distinct maturation rates, with At2g20920 and At2g24090 cTP-GFPs showing significantly higher maturation rates than AtRbcS1A and At1g63970 (**Fig. 4I**). Considering its enhanced targeting activity to chloroplasts, as evidenced by the abundance of precursor proteins and chloroplast-localized mature fluorescent proteins in total proteins, as well as its maturation rate, we unequivocally conclude that At2g24090 cTP is the most efficient in transporting biomolecules into chloroplasts.

**Figure 4. *In vivo* translocation of cTP-GFPs**. (**E**) Immunoblot analysis of recombinant cTP-GFPs in total leaf proteins. Total proteins from agroinfiltrated tobacco leaves at different time points post-infiltration were immunoblotted (WB) against anti-GFP polyclonal antibody to detect the recombinant cTP-GFP precursors (Pre) and the chloroplast-localized matured proteins (Mat) in samples. Equal loading of proteins onto the immunoblot membrane was confirmed by Coomassie Brilliant Blue (CBB) staining of the Rubisco large subunit protein (RbcL) band. (**F**,**G**) Accumulation of various cTP-GFP precursors (**F**) and their matured proteins after translocation to chloroplasts (**G**). The content of recombinant proteins in total leaf proteins was determined from three biologically independent immunoblot experiments against the calibration equation of different amounts of standard GFP (*n* = 3). Error bars represent SD. (**H**) Increase in matured GFP contents in total leaf proteins from 24 to 96 HAI. Error bars represent SD of the means of matured protein contents in total leaf proteins from three biologically independent samples (*n* = 3). Asterisks in (**F**) to (**H**) indicate statistically significant differences analyzed by ANOVA with Dunnett’s multiple comparisons against the AtRbcS1A-GFP contents (*; *P* ≤ 0.05, **; *P* ≤ 0.01, ***; *P* ≤ 0.001, and ****; *P* < 0.0001). n.s. represent no significant difference. (**I**) Maturation rate constants of different cTP-GFP. Rate constants were determined from linear regression equations of matured protein contents in total leaf proteins at 24 to 48 HAI. Error bars represent SD of the average of rate constants from three experiments (*n* = 3). Letters in (**I**) indicate different levels of statistically significant difference of means analyzed by one-way ANOVA with Tukey’s HSD test at *p* = 0.05.

In response to your feedback, we have revised the manuscript by incorporating our new *in vivo* expression study and time-course analysis of various recombinant cTP-GFPs in transformed tobacco leaf cells, as depicted in **Fig. 4**. Detailed information regarding this figure has been included in the supporting information as “Supplementary Figures S14 and S15”. Furthermore, we have updated the main text of this revised manuscript to reflect these changes as follows:

**Figure 4. *In vivo* translocation of cTP-GFPs**. (**A**) Transcript expression of *cTP-GFP* genes in agroinfiltrated tobacco leaves at different time points post-agroinfiltration analyzed by qRT-PCR using *GFP*-specific primers. The expression levels of *cTP-GFP* transcripts were compared to that of pBI121-transformed leaves (EV) at 24 hours post-infiltration. Relative transcript expression was determined from three biologically independent agroinfiltrated leaves (*n* = 3). Error bars represent standard deviation (SD). (**B**) Abundance of various cTP-GFP fluorescent proteins in total leaf proteins. GFP fluorescence in total proteins extracted from agroinfiltrated tobacco leaves at different time points was measured, and the amounts of fluorescent protein in three experimentally independent samples (*n* = 3) were calculated from the linear regression equations generated from the fluorescent intensities of standard GFP. Error bars represent SD. (**C**) Localization of various recombinant cTP-GFPs in transfected tobacco leaf cells at different time points post-agroinfiltration. Scale bars: 20 µm. (**D**) Ratio of GFP fluorescence in chloroplasts to total GFP fluorescence in a CLSM image. CLSM images were captured from transformed plant leaves at different time points. Fluorescent intensities of recombinant cTP-GFPs within chloroplasts and in an ROI of a CSLM image were measured using Fiji ImageJ. Error bars represent SD of sixteen CLSM images (*n* = 16) collected from three independent experiments. (**E**) Immunoblot analysis of recombinant cTP-GFPs in total leaf proteins. Total proteins from agroinfiltrated tobacco leaves at different time points post-infiltration were immunoblotted (WB) against anti-GFP polyclonal antibody to detect the recombinant cTP-GFP precursors (Pre) and the chloroplast-localized matured proteins (Mat) in samples. Equal loading of proteins onto the immunoblot membrane was confirmed by Coomassie Brilliant Blue (CBB) staining of the Rubisco large subunit protein (RbcL) band. (**F**,**G**) Accumulation of various cTP-GFP precursors (**F**) and their matured proteins after translocation to chloroplasts (**G**). The content of recombinant proteins in total leaf proteins was determined from three biologically independent immunoblot experiments against the calibration equation of different amounts of standard GFP (*n* = 3). Error bars represent SD. (**H**) Increase in matured GFP contents in total leaf proteins from 24 to 96 HAI. Error bars represent SD of the means of matured protein contents in total leaf proteins from three biologically independent samples (*n* = 3). Asterisks in (**F**) to (**H**) indicate statistically significant differences analyzed by ANOVA with Dunnett’s multiple comparisons against the AtRbcS1A-GFP contents (*; *P* ≤ 0.05, **; *P* ≤ 0.01, ***; *P* ≤ 0.001, and ****; *P* < 0.0001). n.s. represent no significant difference. (**I**) Maturation rate constants of different cTP-GFP. Rate constants were determined from linear regression equations of matured protein contents in total leaf proteins at 24 to 48 HAI. Error bars represent SD of the average of rate constants from three experiments (*n* = 3). Letters in (**I**) indicate different levels of statistically significant difference of means analyzed by one-way ANOVA with Tukey’s HSD test at *p* = 0.05.

**Figure S14.** Ratio of GFP fluorescence in chloroplasts to total GFP in tobacco leaf cells after agroinfiltration

GFP fluorescence in plant cells and within chloroplasts was quantified using Fiji ImageJ in confocal laser scanning microscopy (CLSM) images of transfected tobacco leaf cells expressing different recombinant cTP-GFP constructs. The box plot illustrates the distribution of fluorescence ratios across sixteen CLSM images (*n* = 16) collected from three biologically independent leaves at various time points post-agroinfiltration. The central bars indicate the median fluorescence ratios at each time point, while the upper and lower bars depict the maximum and minimum values, respectively. Asterisks denote different levels of statistical significance in the mean fluorescence ratios at each time point compared to AtRbcS1A-GFP, used as a control (*; *P* ≤ 0.05, **; *P* ≤ 0.01, ***; *P* ≤ 0.001, and ****; *P* < 0.0001). “n.s.” indicates no significant difference in mean fluorescence ratio compared to the control.

**Figure S15.** *In vivo* transport of recombinant cTP-GFP to chloroplasts

The transport of recombinant cTP-GFP to chloroplasts in transfected tobacco leaf cells was analyzed at 24, 36, 48, 60, 72, and 96 hours after agroinfiltration using immunoblotting. The presence of various cTP-GFP precursors (Pre) and their chloroplast-localized cleaved products (Mat) in total leaf proteins was detected using an anti-GFP polyclonal antibody. The intensity of the GFP band in each sample was then compared to a linear regression equation generated using different amounts of standard GFP. RbcL indicates the major Rubisco large subunit protein band on the immunoblot membrane stained with Coomassie Brilliant Blue.

**Throughout this revised manuscript,** we have changed the term “translocation efficiency (efficiencies)” to “import efficiency (efficiencies)” to avoid confusing readers.

**Page 10 – 13, line 227 – 290**; we added the explanation of experimental data and our statement regarding *in vivo* import activities of different cTP-GFPs into chloroplasts to the “**RESULTS**” section.

“***In vivo* import efficiencies of the selected cTPs**

Targeting a chloroplast transit peptide (cTP)-fused fluorescent protein serves as a pivotal marker, with its abundance within the designated organelle being regulated through distinct mechanisms, including mRNA expression, protein translation, stability, and proper folding and formation of the fluorophores, both within the broader cellular context and specifically within chloroplasts. These mechanisms collectively influence the efficiency with which various recombinant cTP-GFPs are imported into chloroplasts. To explore the dynamics of chloroplast import, we conducted a time-course expression analysis focusing on specific cTPs (At1g63970, At2g20920, and At2g24090), using AtRbcS1A as a reference cTP. This involved the agroinfiltration of expression cassettes containing the selected cTP-GFPs, alongside controls such as free GFP for fluorescence comparison and a negative control vector (pBI121-EV), into tobacco leaves. Subsequent leaf sampling at various time points post-agroinfiltration (24, 36, 48, 60, 72, and 96 hours) enabled comprehensive expression profiling and *in vivo* import assessments.

Quantitative reverse-transcription PCR (qRT–PCR) and fluorometric measurements were employed to evaluate transcript expression and accumulation of recombinant cTP-GFPs in transformed plant leaves, respectively. Our transcript expression analysis unveiled peak expression levels of all *cTP-GFP* transcripts in agroinfiltrated plant leaves at 36 hours after infiltration (HAI), followed by a gradual decline from 48 to 96 HAI (**Fig. 4A**). Interestingly, significant variations in transcript levels were observed among samples transformed with different cTP-GFP expression vectors at these time points (**Fig. 4A**). Fluorescence measurement revealed higher accumulation of fluorescent proteins in total leaf proteins extracted from leaves of plants transformed with cTP-GFPs and GFP-expression vectors compared to the EV control at 36 to 96 HAI (**Fig. 4B**). However, no statistically significant differences in accumulation of fluorescent proteins were detected among the leaves of plants transformed with different cTP-GFP constructs (**Fig. 4B**). Unexpectedly, despite constitutive expression of various *cTP-GFP* genes, higher transcript levels did not translate into significantly greater accumulation of recombinant cTP-GFP in transformed plant leaves (**Fig. 4A,B**).

The localization of recombinant cTP-GFPs within chloroplasts was investigated using CLSM imaging, followed by quantification of GFP fluorescence both in plant cells and within chloroplasts. Surprisingly, AtRbcS1A-GFP was found to partially localize in the cytosol from 24 to 60 HAI, gradually transitioning to chloroplast targeting from 72 to 96 HAI (**Fig. 4C,D**, Fig S14). In contrast, recombinant At1g63970-, At2g20920-, and At2g24090-GFP specifically localized within chloroplasts from 24 to 96 HAI (**Fig. 4C,D**, Fig S14). Remarkably, the fluorescent intensities of chloroplast-localized At1g63970-, At2g20920-, and At2g24090-GFPs were higher than those of AtRbcS1A-GFP at all time points post-agroinfiltration (**Fig. 4D**, Fig S14).

Furthermore, the chloroplast import activities of different recombinant cTP-GFPs were investigated using immunoblot analysis. Total leaf proteins were extracted from transformed leaf samples collected at 24 to 96 HAI and immunoblotted against an anti-GFP antibody. The immunoblotting results revealed differential accumulations of precursor cTP-GFPs and their mature chloroplast-localized forms in total leaf proteins (**Fig. 4E**, Fig S15). A gradual decrease in AtRbcS1A-GFP precursors and an increase in its mature proteins were detected over 24 to 96 HAI in transformed leaves (**Fig. 4E–G**, Fig S15). Although cTP-GFP precursors of At1g63970- and At2g20920-GFPs were also detected up to 48 HAI, their contents in total leaf proteins were significantly lower than that of AtRbcS1A-GFP at the same time points (**Fig. 4E**, Fig S15). However, no detectable At2g24090-GFP precursor was observed on the immunoblot membrane (**Fig. 4E**, Fig S15). Additionally, quantitative immunoblot analysis revealed significantly higher accumulations of mature At1g63970-, At2g20920-, and At2g24090-GFPs in total leaf proteins compared to mature AtRbcS1A-GFP (**Fig. 4G**). The accumulations of these three mature cTP-GFPs in total leaf proteins progressively increased up to 48 HAI, with a significantly higher maturation rate than AtRbcS1A-GFP (**Fig. 4H,I**). However, unlike mature At2g20920- and At2g24090-GFPs, which showed no degradation after 48 HAI, mature At1g63970-GFP gradually degraded over the subsequent period post-infiltration (**Fig. 4E** and **G**, Fig S15). Our CLSM imaging and immunoblot analysis suggest that, despite differential degradation of mature variants, the three selected cTPs have higher import efficiencies in transporting GFP into chloroplasts in transformed plant cells compared to the reference AtRbcS1A cTP.”

**Page 13, line 295 – 301**; In “**RESULTS**” section, the statement regarding the selection of a suitable cTP (At2g24090) for further utilization in biotechnological approaches has been modified.

“The current findings underscore the exceptional import activities of the selected cTPs including At1g63970, At2g20920, and At2g24090 in delivering conjugated GFP to chloroplasts in tobacco leaf cells, surpassing that of other scanned cTPs. The complimentary amino acid sequence, typically associated with the proximal length of the cTP, interacts with the chloroplast import machinery such as proteins responsible for scaffold formation and quality control in the cytosol.”

**Page 20, line 476 – 485**; we modified the “**Discussion**” section by adding a statement describing the difference of our results *in vitro* and *in vivo* chloroplast import activities of various cTPs and our prospectives to further investigation as follow;

“However, a meticulous inspection of the time-course expression of recombinant cTP-GFPs in agroinfiltrated plant cells highlights markedly superior abilities of the selected cTPs (At1g63970, At2g20920, and At2g24090) over the reference cTP AtRbcS1A in facilitating protein import into chloroplasts. This implies that supplementary factors or regulatory mechanisms could influence their import efficiencies *in vivo*, including scaffold formation with protein partners and conformational changes necessary for domain recognition during protein import into plastids. Indeed, distinguishing the translocation mechanisms of an ideal cTP that demonstrates the highest chloroplast import function may necessitate further experimental approaches.”

**Page 28, line 659 – 670**; The following explanation regarding fluorescence-based measurement of recombinant cTP-GFPs in total leaf proteins has been added to the “**Materials and Methods**” section.

“**Fluorescence measurement**

For the measurement of recombinant fluorescent protein content, total leaf proteins were extracted from agroinfiltrated tobacco leaves collected at different hours post infiltration using 100 mM Tris-HCl (pH 7.4) + 150 mM NaCl + 0.1% (v/v) Triton X-100 at 20380 x g for 15 minutes. One-hundred microliters of the sample were applied to a well of a FluoTrac 96-well microplate (Greiner Bio-One, Tokyo, Japan). Various amounts of standard GFP purified from *E. coli* cells were utilized to generate a linear regression equation for calculating the fluorescent protein content in the samples. Fluorescence in total proteins was determined using the fluorescence mode in a Spark 10M microplate reader (Tecan, Männedorf, Switzerland). The concentration of total leaf proteins was measured using Bradford reagent with bovine serum albumin (BSA) as the standard (Bio-Rad).”

**Minor points**

>> **p3, lines 60-61**: 'N-terminal cTPs are usually located 20 to >100 amino acids upstream of preproteins'. The cTP is part of the pre-protein, and thus is not 'upstream' of it. This should be re-phrased, e.g. 'N-terminal cTPs generally encompass the first 20 to 100 amino acids of the preproteins'.

**Response**: Thank you very much for your suggestion. We revised this sentence as the reviewer recommended.

**Page 3, line 60 – 61**;

“N-terminal cTPs typically encompass the first 20 to 100 amino acids of the preproteins”

>> **p4, lines 86-87**: 'However, to date, a comparison of the translocation efficiencies of cTPs has not been performed'. This statement is simply not true. Many studies comparing different cTPs have been performed by different groups. They may not have included as many CTPs as were tested here, or the focus of the study may have been different, but it would be unfair not to mention some here. As a couple of examples:
>> Chiung-Chih Chu, Krishna Swamy, Hsou-min Li, Tissue-Specific Regulation of Plastid Protein Import via Transit-Peptide Motifs, The Plant Cell, Volume 32, Issue 4, April 2020, Pages 1204-1217, [https://doi.org/10.1105/tpc.19.00702](https://doi.org/10.1105/tpc.19.00702)
>> Eseverri Álvaro, Baysal Can, Medina Vicente, Capell Teresa, Christou Paul, Rubio Luis M., Caro Elena. Transit Peptides From Photosynthesis-Related Proteins Mediate Import of a Marker Protein Into Different Plastid Types and Within Different Species. Frontiers in Plant Science 11 (2020) DOI=10.3389/fpls.2020.560701

**Response**: The authors appreciate your kind recommendation to mention about some of comparative studies of import efficiency of different cTPs and included these examples in our manuscript. We have gone thoroughly into these articles and found that our current statement regarding this issue is totally not equitable. We apologize for our unawareness and would like to revise our statement as you recommended. Regarding this revision, we modified the following statement and included the citations according to your suggestion.

**Page 4, line 86 – 89**;

“Until now, the comparison of import efficiencies among numerous cTPs has been experimentally performed using various techniques, including *in vitro* import assays, *in vivo* protoplast transfection, and expression studies in transgenic plant cells [27,28].”

>> **p6, lines 131-132**: 'we did not observe sequence similarity in cleavage sites among the predicted cTPs and tTPs'. As Fig S1 shows, there is clear conservation of amino acids around cleavage sites, as one would expect. So I do not understand why the authors say the contrary in the main text. Incidentally, in Fig S1, the authors should indicate exactly where the cleavage sites are, in panels B and C.

**Response**: Thank you very much for your advice. We have extensively repeated the computational predictions and performed additional multiple peptide sequence alignments using sequence logos. Our analysis focused on the five predicted amino acids likely responsible for cleavage, as well as the four adjacent amino acids on both the N- and C-termini. This revealed a more reliable sequence similarity in the cleavage sites of the selected 89 peptides. The results in Fig S1B and S1C showed that the conserved cleavage sites of the predicted 81 cTPs and 8 tTPs (position -3 to 1) generally consist of an alanine at position -1, which is adjacent to the cleaved amino acid (position 0) that remains in the cTP sequences after cleavage (Fig S1B and S1C).

Furthermore, we analyzed the conservation of cleavage sequences in cTPs that direct GFP to different subcellular localizations in transformed plant cells. Our multiple sequence alignment of cTPs revealed that the cleavage sites of cTPs, which exhibit chloroplast-targeting activity (Chl) and dual import functions to chloroplast and mitochondria or chloroplast and cytosol (Chl/Mt and Chl/Cy), comprise a conserved cleavage sequence (Fig S10). This conserved cleavage sequence generally comprises VR(A/C)(A/S)X, where X is any amino acid, with the two alanines or their interchangeable cysteine and serine at the decisive residues (positions -1 and 0) being highly conserved (Fig S10). This conserved cleavage site is entirely different from that of the other predicted cTPs that mistarget GFP to the cytosol (Fig S10). This new result suggests that the cleavage sites on the chloroplast-specific cTPs are conserved and the presence of the two (A/C)(A/S) residues at the cleavable sequence in the cleavage site may govern the import activity of cTPs.

To revise this manuscript according to thie new information and in corporation with the reviewer’s comments, we have modified our manuscript as follows:

**Figure S1B** and **S1C** have been modified and their figure captions were re-phrased.

**Figure S1**. Computational prediction of signal peptides and their cleavage sites in 89 polypeptide sequences

(**A**) Distribution of mTP scores in 89 polypeptides predicted by TargetP-2.0. The mTP score of each transit peptide is shown as a box plot. Magenta dots represent the mTP scores of each peptide predicted to target the chloroplast (cTP), thylakoid lumen (tTP), and other organelles in plant cells (other). Significant differences are indicated by different letters in the boxplot (one-way ANOVA with Tukey’s HSD test at *p* = 0.05). (**B**) Amino acid distribution in 13 amino acids, which encompassed the predicted cleavage sites of each transit peptides (cTPs). (**C**) Alignment of the predicted cleavage sites of eight tTPs. Multiple sequence alignments were performed using WebLogo. Five amino acids positioned within the magenta box at -3 to 1 represent the predicted cleavage site of the cTPs or tTPs with an extension of 4 amino acids applied to both its N- (-7 to -4) and C-termini (2 to 5). Amino acid at position 0 is the cleaved amino acid, which is the last amino acid attached to each transit peptide after cleavage.

**Page 6, line 132 – 135**; a statement regarding seqeunce similarity of the cleavage sites has been revised.

“Notably, we observed sequence similarity in the cleavage sites among the predicted cTPs and tTPs, both of which usually contain at least a conserved alanine adjacent to the cleaved amino acids (Fig S1B,C, Table S2).”

**Figure S10**, which shows the conservation of the amino acid sequence at the cleavage sites of cTPs, has been added to the “**SUPPLEMENTARY INFORMATION**” section.

**Figure S10**. Distribution of amino acids in the 13 residues surrounding the predicted cleavage sites of each transit peptide (cTPs). The alignment encompasses the cleavage sites of cTPs demonstrating chloroplast-specific targeting of GFP (Chl), dual-targeting cTPs that transport GFP to both chloroplasts and mitochondria (Chl/Mt) or to the cytosol (Chl/Cy), and the predicted cTPs that mislocalize GFP to the cytosol instead of the chloroplast (Cy). Multiple sequence alignments were conducted using WebLogo. The five amino acids within the magenta box at positions -3 to 1 represent the predicted cleavage site of the cTPs or tTPs, along with an additional four amino acids on both the N-terminus (-7 to -4) and C-terminus (2 to 5). The amino acid at position 0 corresponds to the cleaved residue, which constitutes the final amino acid attached to each transit peptide after cleavage. The number in the brackets denotes the count of cleavage sequences of the predicted cTPs in each group.

**Page 7, line 154 – 158**; we added an explanation regarding our new result of conserved cleavage sites in the chroloplast-specific cTPs in the “**RESULTS**” section.

“Furthermore, multiple sequence alignment unveiled a conserved cleavage sequence, VR(A/C)(A/S)X, where X represents any amino acid, within 48 cTPs that specifically target GFP transport to chloroplasts (Chl), along with the predicted cTPs demonstrating dual targeting activities (Chl/Mt and Chl/Cy) (Fig S10)”

**Page 21 – 22, line 510 – 514**; the “**DISCUSSION**” section has been modified by adding the following statement to postulate our strategy to improve the stability of At2g24090 cTP against cellular peptidase and potentially increase import efficiency of this cTP.

“Switching the obstructive cleavage site of the At2g24090 transit peptide with the conserved sequence that we have currently identified holds promise for enhancing the stability of recombinant proteins against cellular peptidases. This modification could lead to heightened chloroplast import activity of the engineered cTPs, thus potentially enhancing their functional efficiency.”

>> **p7, lines 148-150**: 'Despite their alternative localizations in mitochondria and cytosol, cTP-GFPs targeted to chloroplasts exhibited higher GFP fluorescence than their cytosolic counterparts'. The GFP signal has been normalised with respect to the chlorophyll autofluorescence signal. This seems reasonable for GFP signals within chloroplasts, as the two signals should be largely co-located, but not for GFP signals in other cellular compartments, where there is no reason to expect a strong correlation with chlorophyll autofluorescence. Indeed they may even be anti-correlated — a focal plane through a part of the cell cytoplasm with many chloroplasts may have relatively little cytosol as the two compartments are mutually exclusive. I don't think the authors can meaningfully compare GFP fluorescence signals between different compartments using this method. Comparing signals on western blots would probably be better.

**Response**: Thank you for your insightful feedback. We understand your concerns about the validity of comparing GFP signals in different cellular compartments. In our library screening, we tracked the activities of predicted cTPs in transporting GFP to chloroplasts or other plant compartments primarily using CLSM imaging. Initially, we anticipated that all predicted cTPs would exhibit chloroplast-specific translocation of GFP, with the resulting GFP signals perfectly aligned with chlorophyll autofluorescence. We anticipated to compare their chloroplast import efficiencies using the correlation coefficient between GFP and chlorophyll autofluorescence. However, we unexpectedly found that some predicted cTPs displayed dual-targeting activities to mitochondria, while others partially targeted GFP to the cytoplasm. Consequently, we continued our analysis, understanding that comparing the efficiencies of different cTPs using this correlation was inappropriate.

In this manuscript, we presented CLSM image analysis data, separating total GFP/chlorophyll fluorescence from chloroplast-specific GFP/chlorophyll fluorescence, to show the proportional functions of different cTPs in importing GFP to chloroplasts or other plant compartments (**Fig. 1G**). The significantly higher chloroplast GFP signals in cTP-GFPs specifically targeted to chloroplasts, compared to those mistargeted to the cytosol, led us to describe that “cTP-GFPs targeted to chloroplasts exhibited stronger GFP fluorescence than their cytosolic counterparts” (**Fig. 1G**). Additionally, comparing signals on Western blot membranes was not appropriate due to the limited detectable levels of some cTP-GFPs, sometimes below the sensitivity of detection by Western blotting.

To address the reviewer's concern, we prepared a new figure (**Fig. 1H**) to show the proportion of GFP fluorescence in chloroplasts relative to the total GFP fluorescence produced by four different groups of cTP-GFPs targeted to various compartments. This figure provides clearer information, demonstrating the significantly higher efficiencies of chloroplast-specific cTP-GFPs (Chl) compared to other groups of cTP-GFPs that exhibited dual-targeting or mislocalization (Chl/Mt, Chl/Cy, and Cy). This proportional illustration also eliminates the effects of chlorophyll autofluorescence on the determination of cTP-GFP import abilities.

**Figure 1. Screening of high-efficiency chloroplast-targeting peptides. G**) Distribution of GFP/chlorophyll fluorescence of recombinant cTP-GFP in plant cells (total GFP) and chloroplasts (chloroplast GFP). (**H**) Ratios of chloroplast GFP to total GFP in fluorescence images of the four groups of cTP-GFPs exhibiting different subcellular compartmentalization. Different letters in (**G**) and (**H**) indicate significant differences in the means among the groups of cTP-GFPs, analyzed by one-way ANOVA with Tukey’s HSD test at *p* = 0.05.

Based on this new data, we have revised the main text of our manuscript as follows:

**Page 7, line 151 – 154**; the statement abouth the difference in GFP fluorescence within chloroplasts localized by different groups of cTP-GFPs has been revised.

“Fluorescence measurements in the CLSM images demonstrated that the group of chloroplast-specific cTP-GFPs (Chl) exhibited significantly stronger GFP signals within chloroplasts compared to other groups, particularly those with GFP localized to the cytoplasm (**Fig. 1G,H**, Fig S9, Table S3).”

**Fig. 1H** has been added to **Fig. 1**. The legend of **Fig. 1** was also modified.

**Figure 1. Screening of high-efficiency chloroplast-targeting peptides.** (**A**) Workflow outlining sequence filtering and collection of plastidial protein sequences from the Chloroplast Mutant Database for computational analysis and identification of putative transit peptide sequences from plastidial proteins. (**B,C**) Computational prediction of chloroplast-targeting peptides (cTPs), thylakoid-targeting peptides (tTPs), and peptides potentially targeting other organelles in plant cells (referred to as “other”) using the N-termini of the 89 plastidial proteins selected for this study. The cTP score and tTP score indicate the likelihood of the predicted peptides being targeted to the chloroplast matrix and thylakoid, respectively. (**D**) Predicted lengths of cTPs, including their possible cleavage sites. (**E**) Subcellular localizations of different cTP-GFPs in tobacco leaf cells. Arrows show chloroplasts. Scale bars = 20 μm. (**F**) Euler diagram of the differential subcellular localizations of 89 cTP-GFPs in plant cells. Chl = Chloroplast, Cy = Cytosol, Mt = Mitochondria (**G**) Distribution of GFP/chlorophyll fluorescence of recombinant cTP-GFP in plant cells (total GFP) and chloroplasts (chloroplast GFP). (**H**) Ratios of chloroplast GFP to total GFP in fluorescence images of the four groups of cTP-GFPs exhibiting different subcellular compartmentalization. Different letters in (**G**) and (**H**) indicate significant differences in the means among the groups of cTP-GFPs, analyzed by one-way ANOVA with Tukey’s HSD test at *p* = 0.05. (**I**) Classification of the chloroplast-targeting activities of the cTPs. The 89 cTP-GFPs were categorized into four groups based on relative fluorescence intensities in plant cells (Total GFP) vs. inside the chloroplast (Chloroplast GFP) using the *K*-means clustering algorithm. The number of predicted cTPs for each gene locus is listed in Table S3.

>> **p10, lines 223-224**: 'Chloroplast-targeting peptides (cTPs) are evolutionarily designed to interact with the chloroplast import machinery'. As I'm sure the authors are aware, evolution does not design anything, so this is inappropriate phrasing. I don't think the sentence is necessary, I recommend deleting it.

**Response**: Thank you very much for your suggestion. We re-phrased our statement withing the same section as follow:

**Page 13, line 295 – 301**;

“The current findings underscore the exceptional import activities of the selected cTPs including At1g63970, At2g20920, and At2g24090 in delivering conjugated GFP to chloroplasts in tobacco leaf cells, surpassing that of other scanned cTPs. The complimentary amino acid sequence, typically associated with the proximal length of the cTP, interacts with the chloroplast import machinery such as proteins responsible for scaffold formation and quality control in the cytosol.”

>> **Figure 5**: In these experiments, there is the theoretical possibility of inducing silencing of the endogenous L35 gene, leading to potential effects on chloroplast biogenesis and metabolism. Can the authors rule out this possibility? I would like to see some discussion of this, and ideally some evidence showing that this is not an issue.

**Response**: The authors appreciate the reviewer’s critical concern about the potential gene silencing effect that may occur during the exogenous expression of L35 cTP (At2g24090 cTP)-fused proteins in heterologous host plants. Transgene-induced silencing in plants is a form of gene expression suppression caused by the formation of double-stranded RNA (dsRNA) molecules between endogenous single-stranded RNA (ssRNA) and the heterologenously expressed complementary ssRNA molecules, which are overexpressed in plant cells by an exogenous expression cassette. These dsRNA molecules are susceptible to cleavage by the RNA-induced silencing complex (RISC), leading to dsRNA decay and suppressed gene expression in transformed plant cells.

To investigate the potential for *Arabidopsis*-derived *At2g24090 cTP* gene overexpression to induce silencing of *L35* gene expression in tobacco (*Nicotiana tabacum*) and tomato (*Solanum lycopersicum*), we conducted *in silico* multiple sequence alignment of the *L35* genes from these three species. Our computational analysis, as illustrated in *Appendix Figure 1*, compared the sequences and revealed no discernible similarity between the *At2g24090 cTP* gene and the N-terminal portions of endogenous L35-coding sequence of tobacco and tomato. This compelling evidence suggests that the likelihood of dsRNA-induced transgene silencing to suppress the expression of endogenous *L35* genes following the heterologous expression of At2g24090 cTP-fused proteins in these two plant species is minimal, both at the transcriptional and post-transcriptional levels. Furthermore, our study employed an *Agrobacterium*-based transient expression system to overexpress the recombinant cTP-fused proteins, ensuring robust and controlled gene expression. This approach effectively circumvents potential issues related to gene silencing by position effect and chromosome reorganization, which typically arise from the insertion of gene expression cassettes into plant chromosomes. Therefore, we are confident that our experimental setup provides a reliable framework for investigating the functional properties of the At2g24090 cTP in heterologous plant systems.

However, we would like to emphasize the possibilities of transgene silencing and potential limitations following transient expression of exogenous proteins in plant cells, as raised by the reviewer. We also provide our perspective on addressing these occurrences through further research in this manuscript. Regarding this, we included a statement confering this issue in the “**Discussion**” section as follow:

**Page 23, line 539 – 545**;

“Expanding the application of cTP-conjugated biomolecule delivery to plastids necessitates careful development due to potential variations in import activity among different target plant cells. Moreover, comprehensive assessment of potential side effects such as silencing of endogenous gene expression, cryptic protein formation, and cytotoxicity of highly abundant recombinant proteins in transfected cells is essential following the introduction of cTP-engineered cargo molecules into native plant systems.”

***Appredix Figure 1***. *Multiple sequence alignment of ribosomal protein L35*

*Arabidopsis thaliana* ribosomal protein L35 (At2g24090; NC_003071) sequence was used as a quary to search for similar proteins in tomato (*S. lycopersicum*) and tobacco (*N. tabacum*) proteins databases (available in https://solgenomics.net) using blastp algorithm. Coding sequences (CDS) of three tobacco L35 proteins (mRNA26195, mRNA26194, and mRNA129014) and tomato L35 protein (Solyc07g006000) were archieved from plant gonome databases. Multiple CDS alignments was performed using CLUSTALW (<https://www.genome.jp/tools-bin/clustalw>). Arrow indicated a nucletide sequence corresponding to At2g24090 cTP (including its cleavage site).

**Reviewer 2**:
The paper titled "Identification of a Highly Efficient Chloroplast-Targeting Peptide for Plastid Engineering" authored by Thagun et al. aims to identify an optimal chloroplast targeting peptide (CTP) for biotechnological applications. The study involves the evaluation of 89 CTP candidates selected from Arabidopsis chloroplast proteins, wherein these candidates were fused with GFP in a transient expression system using tobacco plants. Among these candidates, the CTP from At2g24090 exhibited the best performance. Subsequently, this top-performing CTP was further assessed by fusion with enzymes to demonstrate its enhanced accumulation and efficient chloroplast localization for the selected enzymes.

While the proposed CTPs hold promise for advancing chloroplast biotechnology, there are several major concerns that require attention for a more thorough and clear explanation. Please consider the following suggestions for revision.

**Response**: We sincerely appreciate the reviewer’s valuable time and effort in helping us improve our manuscript. Your detailed comments and suggestions were thoroughly considered. Please see our point-by-point responses to your comments below.

>> **Major comments**
**1**) The term "translocation efficiency" and its associated explanations should be carefully reviewed and revised throughout the manuscript. The authors primarily assessed the accumulation of fused proteins in chloroplasts but did not directly measure "translocation efficiency." Translocation efficiency typically involves determining the ratio of protein content within chloroplasts to the total protein content or examining the speed of protein translocation from the cytosol to the chloroplast. It is suggested that the observed effective accumulation of proteins using the CTP from At2g20490 may be attributed to the efficiency of transcription and translation processes rather than the effects of "translocation efficiency." Therefore, the manuscript should provide a more accurate and clear representation of the experimental results and their implications.
**Response**: Thank you very much for your valuable comment regarding the terminology “translocation efficiency” used to describe the varying import activities of various cTPs in our manuscript. In the current version of our manuscript, we only assessed the “translocation efficiencies” of the scanned cTPs at the endpoint (generally at day 3 post-infiltration). Although we attempted to validate the differential activities of the top 3 cTPs (At1g63970, At2g20920, and At2g24090) compared to the reference AtRbcS1A cTP in transporting recombinant GFP into isolated chloroplasts using *in vitro* import assays at various time points (**Fig. 3**), this experimental approach could not fully evaluate the differences in import efficiency among the tested cTPs. We anticipate that the variance in efficient translocation (import) of chloroplast proteins mediated by cTPs may involve other mechanisms, such as cytosolic scaffold proteins and plant-specific chaperones, which regulate the stability of recombinant cTP-GFPs in plant cells upon transport to chloroplasts. Furthermore, as the reviewer mentioned, the translocation activities of different cTP-GFPs may be influenced by differential mRNA expression and protein translation.

To show a clearer experimental result confering the difference of “translocation (import) efficiency” among the chosen cTPs (At1g63970, At2g20920, At2g24090, and AtRbcS1A), we performed additional *in vivo* expression study of the recombinant cTP-GFPs in agroinfiltrated tobacco leaf cells (**Fig. 4**).

**Figure 4. *In vivo* translocation of cTP-GFPs**. (**A**) Transcript expression of *cTP-GFP* genes in agroinfiltrated tobacco leaves at different time points post-agroinfiltration analyzed by qRT-PCR using *GFP*-specific primers. The expression levels of *cTP-GFP* transcripts were compared to that of pBI121-transformed leaves (EV) at 24 hours post-infiltration. Relative transcript expression was determined from three biologically independent agroinfiltrated leaves (*n* = 3). Error bars represent standard deviation (SD). (**B**) Abundance of various cTP-GFP fluorescent proteins in total leaf proteins. GFP fluorescence in total proteins extracted from agroinfiltrated tobacco leaves at different time points was measured, and the amounts of fluorescent protein in three experimentally independent samples (*n* = 3) were calculated from the linear regression equations generated from the fluorescent intensities of standard GFP. Error bars represent SD. (**C**) Localization of various recombinant cTP-GFPs in transfected tobacco leaf cells at different time points post-agroinfiltration. Scale bars: 20 µm. (**D**) Ratio of GFP fluorescence in chloroplasts to total GFP fluorescence in a CLSM image. CLSM images were captured from transformed plant leaves at different time points. Fluorescent intensities of recombinant cTP-GFPs within chloroplasts and in an ROI of a CSLM image were measured using Fiji ImageJ. Error bars represent SD of sixteen CLSM images (*n* = 16) collected from three independent experiments. (**E**) Immunoblot analysis of recombinant cTP-GFPs in total leaf proteins. Total proteins from agroinfiltrated tobacco leaves at different time points post-infiltration were immunoblotted (WB) against anti-GFP polyclonal antibody to detect the recombinant cTP-GFP precursors (Pre) and the chloroplast-localized matured proteins (Mat) in samples. Equal loading of proteins onto the immunoblot membrane was confirmed by Coomassie Brilliant Blue (CBB) staining of the Rubisco large subunit protein (RbcL) band. (**F**,**G**) Accumulation of various cTP-GFP precursors (**F**) and their matured proteins after translocation to chloroplasts (**G**). The content of recombinant proteins in total leaf proteins was determined from three biologically independent immunoblot experiments against the calibration equation of different amounts of standard GFP (*n* = 3). Error bars represent SD. (**H**) Increase in matured GFP contents in total leaf proteins from 24 to 96 HAI. Error bars represent SD of the means of matured protein contents in total leaf proteins from three biologically independent samples (*n* = 3). Asterisks in (**F**) to (**H**) indicate statistically significant differences analyzed by ANOVA with Dunnett’s multiple comparisons against the AtRbcS1A-GFP contents (*; *P* ≤ 0.05, **; *P* ≤ 0.01, ***; *P* ≤ 0.001, and ****; *P* < 0.0001). n.s. represent no significant difference. (**I**) Maturation rate constants of different cTP-GFP. Rate constants were determined from linear regression equations of matured protein contents in total leaf proteins at 24 to 48 HAI. Error bars represent SD of the average of rate constants from three experiments (*n* = 3). Letters in (**I**) indicate different levels of statistically significant difference of means analyzed by one-way ANOVA with Tukey’s HSD test at *p* = 0.05.

In this experiment, we introduced various genetic constructs into tobacco leaves through agroinfiltration. These constructs contained genes encoding different fusion proteins: At1g63970-, At2g20920-, and At2g24090-GFP, along with AtRbcS1A-GFP (used as a reference cTP) and GFP without a transit peptide fusion (free GFP). We subsequently examined the mRNA expression and the accumulation of the corresponding proteins in the plant cells at 24 to 96 hours after infiltration (HAI). Our analysis, conducted using qRT-PCR and fluorometry-based measurements, showed no statistically significant difference in the accumulation of recombinant cTP-GFPs in total leaf proteins, despite variations in transcript expression levels between 24 and 60 hours post-infiltration (**Fig. 4A**,**B**).

Following the expression studies, we investigated the import of these recombinant cTP-GFPs to chloroplasts in transformed plant cells. By using confocal microscopy and image analysis, we observed distinct localization patterns of these proteins within tobacco leaf cells (**Fig. 4C**, Fig. S14). Notably, while AtRbcS1A-GFP initially distributed partially in the cytosol at 24 to 60 HAI before gradually localizing to the chloroplasts at later time points, At1g63970-, At2g20920-, and At2g24090-GFP exhibited stronger chloroplast-specific GFP signals at all time points compared to the AtRbcS1A-GFP control (**Fig. 4C**,**D**, Fig S14).

To further assess chloroplast import activities, we conducted immunoblotting of total leaf proteins collected at different time points post agroinfiltration (**Fig. 4E**, Fig S15). Our results, along with GFP band quantification (**Fig. 4E**-**G**), revealed varying abundances of cTP-GFP precursor proteins and their matured variants. Particularly, At2g24090-GFP displayed undetectable precursor levels, with gradually increasing mature protein levels over time, indicating a high maturation rate (**Fig. 4E**-**G**). Additionally, the maturation rates of the recombinant cTP-GFPs varied, with At2g20920 and At2g24090 cTP-GFPs showing significantly higher rates than AtRbcS1A and At1g63970 (**Fig. 4H**,**I**).

Considering the enhanced targeting activity to chloroplasts, as indicated by precursor and mature protein abundances, and the maturation rate, we conclude that At2g24090 cTP is the most efficient in transporting biomolecules into chloroplasts.

**Figure S14.** Ratio of GFP fluorescence in chloroplasts to total GFP in tobacco leaf cells after agroinfiltration

GFP fluorescence in plant cells and within chloroplasts was quantified using Fiji ImageJ in confocal laser scanning microscopy (CLSM) images of transfected tobacco leaf cells expressing different recombinant cTP-GFP constructs. The box plot illustrates the distribution of fluorescence ratios across sixteen CLSM images (*n* = 16) collected from three biologically independent leaves at various time points post-agroinfiltration. The central bars indicate the median fluorescence ratios at each time point, while the upper and lower bars depict the maximum and minimum values, respectively. Asterisks denote different levels of statistical significance in the mean fluorescence ratios at each time point compared to AtRbcS1A-GFP, used as a control (*; *P* ≤ 0.05, **; *P* ≤ 0.01, ***; *P* ≤ 0.001, and ****; *P* < 0.0001). “n.s.” indicates no significant difference in mean fluorescence ratio compared to the control.

**Figure S15.** *In vivo* transport of recombinant cTP-GFP to chloroplasts

The transport of recombinant cTP-GFP to chloroplasts in transfected tobacco leaf cells was analyzed at 24, 36, 48, 60, 72, and 96 hours after agroinfiltration using immunoblotting. The presence of various cTP-GFP precursors (Pre) and their chloroplast-localized cleaved products (Mat) in total leaf proteins was detected using an anti-GFP polyclonal antibody. The intensity of the GFP band in each sample was then compared to a linear regression equation generated using different amounts of standard GFP. RbcL indicates the major Rubisco large subunit protein band on the immunoblot membrane stained with Coomassie Brilliant Blue.

In response to your comment, we have made revisions to the manuscript by incorporating our new *in vivo* expression study and time-course analysis of various recombinant cTP-GFPs in transformed tobacco leaf cells, as illustrated in **Fig. 4**. Detailed information regarding this figure has been included in the supporting information (Fig S14 and S15)”. Additionally, we have updated the main text of this revised manuscript to reflect these changes as follows:

**Throughout this revised manuscript**, we have replaced the term “translocation efficiency (efficiencies)” with “import efficiency (efficiencies)” to ensure clarity for readers.

**Page 10 – 13, line 227 – 290**; we have added an explanation of the experimental data and our statement regarding the *in vivo* import activities of different cTP-GFPs into chloroplasts to the “**Results**” section.

“***In vivo* import efficiencies of the selected cTPs**

Targeting a chloroplast transit peptide (cTP)-fused fluorescent protein serves as a pivotal marker, with its abundance within the designated organelle being regulated through distinct mechanisms, including mRNA expression, protein translation, stability, and proper folding and formation of the fluorophores, both within the broader cellular context and specifically within chloroplasts. These mechanisms collectively influence the efficiency with which various recombinant cTP-GFPs are imported into chloroplasts. To explore the dynamics of chloroplast import, we conducted a time-course expression analysis focusing on specific cTPs (At1g63970, At2g20920, and At2g24090), using AtRbcS1A as a reference cTP. This involved the agroinfiltration of expression cassettes containing the selected cTP-GFPs, alongside controls such as free GFP for fluorescence comparison and a negative control vector (pBI121-EV), into tobacco leaves. Subsequent leaf sampling at various time points post-agroinfiltration (24, 36, 48, 60, 72, and 96 hours) enabled comprehensive expression profiling and *in vivo* import assessments.

Quantitative reverse-transcription PCR (qRT–PCR) and fluorometric measurements were employed to evaluate transcript expression and accumulation of recombinant cTP-GFPs in transformed plant leaves, respectively. Our transcript expression analysis unveiled peak expression levels of all *cTP-GFP* transcripts in agroinfiltrated plant leaves at 36 hours after infiltration (HAI), followed by a gradual decline from 48 to 96 HAI (**Fig. 4A**). Interestingly, significant variations in transcript levels were observed among samples transformed with different cTP-GFP expression vectors at these time points (**Fig. 4A**). Fluorescence measurement revealed higher accumulation of fluorescent proteins in total leaf proteins extracted from leaves of plants transformed with cTP-GFPs and GFP-expression vectors compared to the EV control at 36 to 96 HAI (**Fig. 4B**). However, no statistically significant differences in accumulation of fluorescent proteins were detected among the leaves of plants transformed with different cTP-GFP constructs (**Fig. 4B**). Unexpectedly, despite constitutive expression of various *cTP-GFP* genes, higher transcript levels did not translate into significantly greater accumulation of recombinant cTP-GFP in transformed plant leaves (**Fig. 4A,B**).

The localization of recombinant cTP-GFPs within chloroplasts was investigated using CLSM imaging, followed by quantification of GFP fluorescence both in plant cells and within chloroplasts. Surprisingly, AtRbcS1A-GFP was found to partially localize in the cytosol from 24 to 60 HAI, gradually transitioning to chloroplast targeting from 72 to 96 HAI (**Fig. 4C,D**, Fig S14). In contrast, recombinant At1g63970-, At2g20920-, and At2g24090-GFP specifically localized within chloroplasts from 24 to 96 HAI (**Fig. 4C,D**, Fig S14). Remarkably, the fluorescent intensities of chloroplast-localized At1g63970-, At2g20920-, and At2g24090-GFPs were higher than those of AtRbcS1A-GFP at all time points post-agroinfiltration (**Fig. 4D**, Fig S14).

Furthermore, the chloroplast import activities of different recombinant cTP-GFPs were investigated using immunoblot analysis. Total leaf proteins were extracted from transformed leaf samples collected at 24 to 96 HAI and immunoblotted against an anti-GFP antibody. The immunoblotting results revealed differential accumulations of precursor cTP-GFPs and their mature chloroplast-localized forms in total leaf proteins (**Fig. 4E**, Fig S15). A gradual decrease in AtRbcS1A-GFP precursors and an increase in its mature proteins were detected over 24 to 96 HAI in transformed leaves (**Fig. 4E–G**, Fig. S15). Although cTP-GFP precursors of At1g63970- and At2g20920-GFPs were also detected up to 48 HAI, their contents in total leaf proteins were significantly lower than that of AtRbcS1A-GFP at the same time points (**Fig. 4E**, Fig S15). However, no detectable At2g24090-GFP precursor was observed on the immunoblot membrane (**Fig. 4E**, Fig S15). Additionally, quantitative immunoblot analysis revealed significantly higher accumulations of mature At1g63970-, At2g20920-, and At2g24090-GFPs in total leaf proteins compared to mature AtRbcS1A-GFP (**Fig. 4G**). The accumulations of these three mature cTP-GFPs in total leaf proteins progressively increased up to 48 HAI, with a significantly higher maturation rate than AtRbcS1A-GFP (**Fig. 4H,I**). However, unlike mature At2g20920- and At2g24090-GFPs, which showed no degradation after 48 HAI, mature At1g63970-GFP gradually degraded over the subsequent period post-infiltration (**Fig. 4E** and **G**, Fig S15). Our CLSM imaging and immunoblot analysis suggest that, despite differential degradation of mature variants, the three selected cTPs have higher import efficiencies in transporting GFP into chloroplasts in transformed plant cells compared to the reference AtRbcS1A cTP.”

**Page 13, line 295 – 301**; In the “**RESULTS**” section, we have updated the description of the strategy used for selecting a suitable cTP (At2g24090) for subsequent biotechnological applications.

“The current findings underscore the exceptional import activities of the selected cTPs including At1g63970, At2g20920, and At2g24090 in delivering conjugated GFP to chloroplasts in tobacco leaf cells, surpassing that of other scanned cTPs. The complimentary amino acid sequence, typically associated with the proximal length of the cTP, interacts with the chloroplast import machinery such as proteins responsible for scaffold formation and quality control in the cytosol.”

**Page 20, line 476 – 485**; We revised the “**DISCUSSION**” section by adding a statement that describes the differences between our *in vitro* and *in vivo* chloroplast import activities of various cTPs, along with our future research prospects, as follow:

“However, a meticulous inspection of the time-course expression of recombinant cTP-GFPs in agroinfiltrated plant cells highlights markedly superior abilities of the selected cTPs (At1g63970, At2g20920, and At2g24090) over the reference cTP AtRbcS1A in facilitating protein import into chloroplasts. This implies that supplementary factors or regulatory mechanisms could influence their import efficiencies *in vivo*, including scaffold formation with protein partners and conformational changes necessary for domain recognition during protein import into plastids. Indeed, distinguishing the translocation mechanisms of an ideal cTP that demonstrates the highest chloroplast import function may necessitate further experimental approaches.”

**Page 28, line 659 – 670**; An explanation about the fluorescence-based measurement of recombinant cTP-GFPs in total leaf proteins has been added to the “**MATERIALS AND METHODS**” section.

“**Fluorescence measurement**

For the measurement of recombinant fluorescent protein content, total leaf proteins were extracted from agroinfiltrated tobacco leaves collected at different hours post infiltration using 100 mM Tris-HCl (pH 7.4) + 150 mM NaCl + 0.1% (v/v) Triton X-100 at 20380 x g for 15 minutes. One-hundred microliters of the sample were applied to a well of a FluoTrac 96-well microplate (Greiner Bio-One, Tokyo, Japan). Various amounts of standard GFP purified from *E. coli* cells were utilized to generate a linear regression equation for calculating the fluorescent protein content in the samples. Fluorescence in total proteins was determined using the fluorescence mode in a Spark 10M microplate reader (Tecan, Männedorf, Switzerland). The concentration of total leaf proteins was measured using Bradford reagent with bovine serum albumin (BSA) as the standard (Bio-Rad).”

**2**) The selection of Arabidopsis CTPs for promoting protein accumulation in chloroplasts was conducted using a transient heterologous system in tobacco. While this approach may be suitable for initial screening, it may not necessarily reflect the effectiveness of these CTPs when applied within the native plant context. The manuscript should acknowledge the limitations of this method and clearly state that further validation or testing within the intended plant species is necessary.

In practical bioengineering applications, the key objective often revolves around achieving higher protein accumulation within chloroplasts, and this should be emphasized and explained more comprehensively in the manuscript. Revisions according to these two major points would enhance the clarity and suitability of the paper for publication.

**Response**: We appreciate reviewer’s kind considerations regarding the limitation of transient expression system and the effectiveness of the representative cTPs in other plant species. We have previously discussed about the strategical validation and improvement of a highy effective cTP (At2g24090) as in the following statement:

**Page 22, line 519 – 523**;

“Additionally, identifying motifs capable of facilitating the import mechanism of At2g24090 cTP could enhance its targeting activity by introducing mutations or performing domain swapping [13,50,51]. Moreover, conducting comparative studies involving At2g24090 cTP homologs from other plant species would expedite the construction of an optimal cTP, thereby maximizing biomolecule targeting to plastids.”

Regarding your concern about the limitation of using transient expression and biomolecule (protein) delivery to plastids in native plant cells, we humbly agree to modify the contents in the “**DISCUSSION**” section of the revised manuscript as follows:

**Page 22 – 23, line 534 – 537**; we added a discussion about the application of transient expression system to facilitate non-genetically modified chloroplast biotechnology and plastome engineering.

“This finding supports pivotal chloroplast engineering and plastome modification mediated by cTP-engineered proteins in the premeditated crop species through non-transgenic approach using transient expression systems [52,53].”

**Page 23, line 539 – 545**; the following statement has been included to the “**DISCUSSION**” section to express our awareness and describe potential drawbacks that may occur following transient expression or artificial introduction of cTP-conjugated biomolecules to plastids in the selected plant species.

“Expanding the application of cTP-conjugated biomolecule delivery to plastids necessitates careful development due to potential variations in import activity among different target plant cells. Moreover, comprehensive assessment of potential side effects such as silencing of endogenous gene expression, cryptic protein formation, and cytotoxicity of highly abundant recombinant proteins in transfected cells is essential following the introduction of cTP-engineered cargo molecules into native plant systems.”

>> **Minor comments**
>> **Line 149**, "higher GFP fluorescence than their cytosol counterparts" would be "Higher GFP fluorescence than their other counterparts in the cytoplasm"

**Response**: Thank you very much for overlooking through this grammatically error in our manuscript. However, in accordance to the recommendation by another reviewer, we have revised this statement according to your suggestion as follow:

**Page 7, line 151 – 154**;

“Fluorescence measurements in the CLSM images demonstrated that the group of chloroplast-specific cTP-GFPs (Chl) exhibited significantly stronger GFP signals within chloroplasts compared to other groups, particularly those with GFP localized to the cytosol (**Fig. 1G,H**, Fig S9, Table S3).”
